# RNA-binding protein RBM5 plays an essential role in acute myeloid leukemia by activating the oncogenic protein HOXA9

**DOI:** 10.1186/s13059-023-03149-8

**Published:** 2024-01-12

**Authors:** Mengli Zhang, Judith Hyle, Xiaowen Chen, Ye Xin, Yingcai Jin, Jianxiang Zhang, Xue Yang, Xinfeng Chen, Shaela Wright, Zhenling Liu, Wojciech Rosikiewicz, Beisi Xu, Liusheng He, Hong Liu, Nana Ping, Depei Wu, Feiqiu Wen, Chunliang Li, Peng Xu

**Affiliations:** 1 Cyrus Tang Medical Institute, National Clinical Research Center for Hematologic Diseases, State Key Laboratory of Radiation Medicine and Protection, Collaborative Innovation Center of Hematology, Soochow University, Suzhou, 215123 Jiangsu China; 2 Department of Tumor Cell Biology, St. Jude Children’s Research Hospital, 262 Danny Thomas Place, Memphis, TN 38105 USA; 3 Division of Hematology and Oncology, Shenzhen Children’s Hospital, Shenzhen Institute of Pediatrics, 7019 Yi Tian Road, Shenzhen, 518038 China; 4 Center for Applied Bioinformatics, St. Jude Children’s Research Hospital, 262 Danny Thomas Place, Memphis, TN 38105 USA; 5 Core Facility of Flow Cytometry, St. Jude Children’s Research Hospital, 262 Danny Thomas Place, Memphis, TN 38105 USA; 6 National Clinical Research Center for Hematologic Diseases, Jiangsu Institute of Hematology, The First Affiliated Hospital of Soochow University, Suzhou, 215123 Jiangsu China

**Keywords:** CRISPR screen, Genome editing, RBM5, HOXA9, Acute myeloid leukemia

## Abstract

**Background:**

The oncogenic protein HOXA9 plays a critical role in leukemia transformation and maintenance, and its aberrant expression is a hallmark of most aggressive acute leukemia. Although inhibiting the upstream regulators of HOXA9 has been proven as a significant therapeutic intervention, the comprehensive regulation network controlling HOXA9 expression in leukemia has not been systematically investigated.

**Results:**

Here, we perform genome-wide CRISPR/Cas9 screening in the HOXA9-driven reporter acute leukemia cells. We identify a poorly characterized RNA-binding protein, RBM5, as the top candidate gene required to maintain leukemia cell fitness. RBM5 is highly overexpressed in acute myeloid leukemia (AML) patients compared to healthy individuals. RBM5 loss triggered by CRISPR knockout and shRNA knockdown significantly impairs leukemia maintenance in vitro and in vivo. Through domain CRISPR screening, we reveal that RBM5 functions through a noncanonical transcriptional regulation circuitry rather than RNA splicing, such an effect depending on DNA-binding domains. By integrative analysis and functional assays, we identify HOXA9 as the downstream target of RBM5. Ectopic expression of HOXA9 rescues impaired leukemia cell proliferation upon RBM5 loss. Importantly, acute protein degradation of RBM5 through auxin-inducible degron system immediately reduces HOXA9 transcription.

**Conclusions:**

We identify RBM5 as a new upstream regulator of HOXA9 and reveal its essential role in controlling the survival of AML. These functional and molecular mechanisms further support RBM5 as a promising therapeutic target for myeloid leukemia treatment.

**Supplementary Information:**

The online version contains supplementary material available at 10.1186/s13059-023-03149-8.

## Background

Acute myeloid leukemia (AML) represents a type of malignant hematological disease that indicates poor outcomes in children and adults [1, 2]. HOXA9 overexpression is observed in most human AML patients and positively correlates with poor patient outcomes [3, 4]. Leukemia subtypes with hallmark overexpression of HOXA9 include but are not limited to those carrying KMT2A gene rearrangements (KMT2A-r), NPM1c mutations, NUP98-translocations, and EZH2 mutation [5]. Accumulating genetic evidence also indicates that HOXA9 dysregulation is sufficient and necessary for leukemic transformation, and loss of function of HOXA9 and downstream pathways consequently impaired leukemia maintenance [6, 7].

Although HOXA9 is not an ideal direct candidate for therapeutic interventions, uncovering the novel regulators of *HOXA9* might elucidate the *HOXA9* regulation mechanism that could be exploited to identify novel therapeutic targets in *HOXA9*-driven leukemias [8]. Several studies showed that therapeutic inhibition of *HOXA9* could be achieved by disrupting upstream regulators, as exemplified by inhibitors targeting DOT1L or MENIN, known as HOXA9’s critical regulators [9–11]. We have previously established a state-of-art cellular tool by inserting the mCherry reporter cassette into endogenous *HOXA9* locus in KMT2A-r OCIAML2 and SEM leukemia cell lines, allowing for unbiased genome-wide screening to reveal HOXA9’s regulators [12]. To this end, by utilizing these valuable reporter lines and a focused CRISPR library against ~ 1600 human transcription factors, we have recently identified a novel positive regulator of HOXA9, USF2, along with other known regulators, including KAT7, ZFP64, and DOT1L [12]. However, despite the functional significance of HOXA9 in leukemia, its functional regulators have not been systematically characterized at a genome-wide scale.

RNA-binding proteins (RBPs) control RNA stability, RNA subcellular localization, translational efficiency, and metabolism by binding mRNAs and noncoding RNAs, thereby controlling the expression and stability of the encoded proteins [13]. Multiple lines of evidence indicated a few RBPs are essential for AML survival and differentiation [14–19]. For instance, the carbonic anhydrase inhibitor, indisulam, induces a ternary protein complex between RBM39 and the E3 ubiquitin complex, resulting in rapid proteasomal degradation of RBM39, aberrant RNA splicing, and cell death of AML [20, 21]. Besides these progresses, the functional role of most RBPs in AML and its therapeutical innovation is still largely unexplored [22–25].

To this end, we have successfully conducted *HOXA9*-reporter-based genome-wide CRISPR/Cas9 screens and unbiasedly identified that candidate genes are significantly enriched in RNA-binding and RNA-splicing regulation. A poorly characterized RBP protein, RBM5, was among the top essential genes. Previously, RBM5 was reported as a tumor suppressor gene in lung cancer [26]. In contrast, a separate study validated that RBM5 is essential for solid tumor cell proliferation [27]. Moreover, several previous literature mainly revealed the role of the RBM5 locus antisense transcribed noncoding RNA, RBM5-AS1, in promoting tumorigenesis [28, 29]. Thus far, the function of the encoding protein RBM5 in blood cancer remains unknown. Here, we have conducted sophisticated functional validation and rescue experiments in human AML cell lines and primary AML cells. We confirmed that RBM5 loss triggered by CRISPR knockout, and shRNA knockdown, significantly impaired AML survival in vitro and in vivo. Ectopic expression of sgRNA-resistant RBM5 cDNA ultimately rescued the cellular phenotype upon RBM5 loss. Moreover, the domain dropout CRISPR screen revealed that the DNA-binding domain C2H2 is required for the gene function. The integrative analysis further suggested that the HOXA9/FLT3 axis promises the dominant downstream target of RBM5, as demonstrated by functional rescue assay and transcriptional regulation in leukemia cells. Notably, RBM5 protein degradation acutely decreased HOXA9 transcription.

In summary, our innovative research reported that a new RNA-binding protein, RBM5, is required for the survival of AML through noncanonical transcriptional regulation via a complex regulatory mechanism. These data support RBM5 as a promising AML therapeutic target in future studies.

## Results

### Genome-wide CRISPR/Cas9 screening identified RNA splicing factor RBM5 as a novel regulator for HOXA9 expression in acute leukemia

**Fig. 1:**
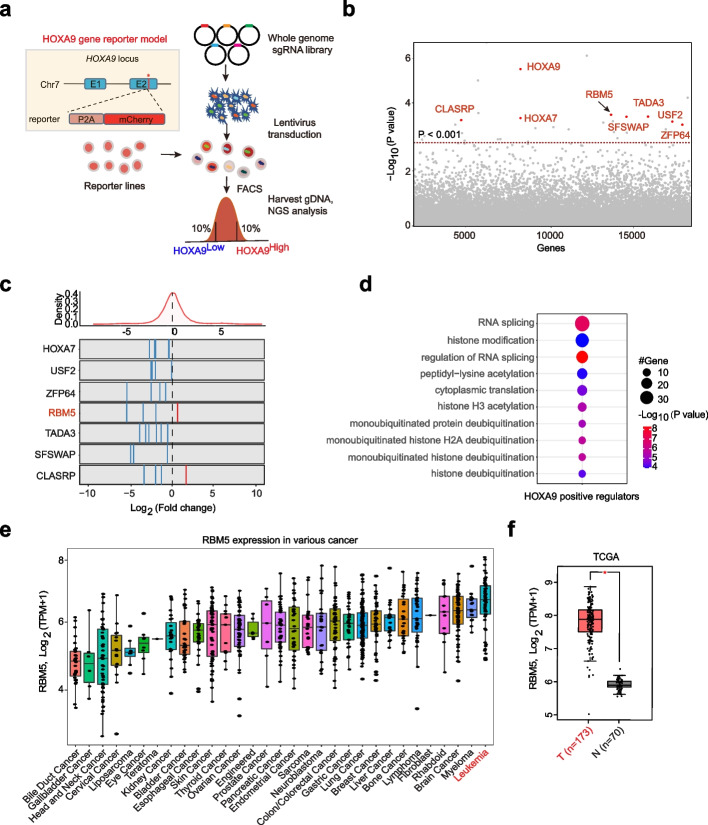
Genome-wide CRISPR/Cas9 screening identifies RNA splicing factor RBM5 as a novel regulator for HOXA9 expression in acute leukemia. **a** Schematic diagram of a working flow of loss-of-function CRISPR screening targeting whole genome gene. **b** The whole-genome-wide CRISPR/Cas9 screen results. Bubble plots show the 19,063 genes identified, genes are known to regulate HOXA9 (HOXA9, HOXA7, ZFP64, USF2), and the novel hits RBM5, TADA3, SFSWAP, and CLASRP are highlighted in red. The *P*-values were calculated using the RSA algorithm. **c** The count ratio for all sgRNAs targeting the HOXA7, USF2, ZFP64, RBM5, TADA3, SFSWAP, and CLASRP is shown between HOXA9^High^ and HOXA9^Low^ sorted populations. The DEseq2 score of each gene was calculated by Log_2_[fold change (HOXA9^High^/HOXA9^Low^)]. **d** Gene Ontology (GO) analysis was performed on the significant genes that positively regulate HOXA9 expression from the screening results. **e** RBM5 mRNA expression level in various cancer cell types from CCLE (Cancer Cell Line Encyclopedia). TPM, transcripts per million. T, tumor/cancer. **f** Box plot comparing the RBM5 mRNA expression level between AML samples (from TCGA dataset) and matched normal samples (from TCGA and GTEx projects). The plot is drawn by using the GEPIA online server. TPM, transcripts per million. T, tumor/cancer. N, normal bone marrow. *P < 0.05, unpaired Student’s *t*-test

Previously, we had successfully established two KMT2A-r leukemia cell lines with the P2A-mCherry cassette insertion into endogenous *HOXA9* locus and fully recapitulated the endogenous gene expression [12]. To systematically and unbiasedly elucidate the *HOXA9*’s regulators and potential therapeutic targets, we performed genome-wide loss-of-function CRISPR/Cas9 screens on the two independent HOXA9-P2A-mCherry reporter lines (SEM and OCIAML2) (Fig. 1a). Two paralleled screens were conducted on these cells constitutively expressing Cas9. The lentiviral sgRNA library-infected cells were further selected and fractionated by flow cytometric sorting for the top 10% (HOXA9^High^) and bottom 10% (HOXA9^Low^) mCherry populations, followed by the quantification of differentially represented sgRNAs against corresponding genes (Fig. 1a). The positive control genes, such as HOXA9, HOXA7, USF2, and ZFP64, were reidentified among the top hits in regulating HOXA9 expression (Fig. 1b, c). We further performed Gene Ontology (GO) enrichment analysis for these positive regulators with a cutoff of *P* < 0.05 and log_2_(fold change) <  − 1 and identified the top significant term associated with RNA splicing (Fig. 1d). These results suggest RNA splicing complex members are critical for HOXA9 regulation. Although previous studies have demonstrated that RNA-binding proteins (RBPs) were frequently mutated in AML patients and essential for leukemia growth [30, 31], how RBPs genes regulate HOXA9 expression and AML survival remains uncharacterized. When we compared the positive regulator hits of HOXA9 from our screen and the RBP genes from DepMap dropout screen results, four genes, RBM5, DHX15, DHX36, and PABPC1, were overlapped (Additional file 1: Fig. S1a; Additional file 2: Table S1). As the role of RBM5 in tumorigenesis was not fully understood from previous reports [26, 27], we decided to further study this top hit in more depth. From our screen, most of the sgRNAs targeting RBM5 are significantly enriched in the HOXA9^Low^ population (Fig. 1c), suggesting an oncoprotein function in leukemia. Beyond the top hit RBM5, we also randomly picked up other top candidates (DHX15, STAG2, MED30, and GTF2A2) for validation. Consistently, the top significant sgRNAs targeting those candidates reduced the HOXA9 mRNA, accompanied by the cellular growth defects (Additional file 1: Fig. S1b, c), suggesting that our reporter-based screens are powerful approaches to identifying new HOXA9 regulators.

To further test whether RBM5 expression might correlate with AML development, we analyzed the public RNA-seq data from the TCGA clinical cancer sample datasets. RBM5 was shown to be the highest expression level in leukemia across all cancer types (Fig. 1e). Moreover, the RBM5 expression level was significantly higher in AML compared to matched normal tissue (Fig. 1f). We further explored the RBM5 expression in a separate patient cohort from St. Jude Cloud [32] and observed that RBM5 is broadly expressed in different molecular subtypes of AML and significantly higher in CEBPA mutated, NPM1-mutated and KMT2A-r subtypes (Additional file 1: Fig. S1d). To further investigate the gene expression signature of AML patients with high expression of RBM5, we ranked 44 AML patient samples from the TCGA dataset based on RBM5 expression level [33] and defined the top 8 of 44 cases (*TPM* > mean + SD) as RBM5 high and the bottom 7 of 44 patients (*TPM* < mean − SD) as RBM5 low (Additional file 1: Fig. S1e). In total, we identified 518 differentially expressed genes (*FDR* < 0.05, Log_2_(fold change) > 1), including 280 genes that increased and 238 genes that decreased in AML patient samples with higher RBM5 expression (Additional file 1: Fig. S1f, Additional file 3: Table S2). Notably, several essential genes involved in HSC self-renewal, metabolism, and leukemia development were increased, including GATA2, MSI2, MYCN, CD44, GAS2, HOXB4, and HOXB6 (Additional file 1: Fig. S1f). Together, those results suggest that RBM5 is highly expressed in AML, indicating a plausible role in AML oncogenesis.

### *RBM5 is essential for the growth of acute myeloid leukemia cells *in vitro

**Fig. 2:**
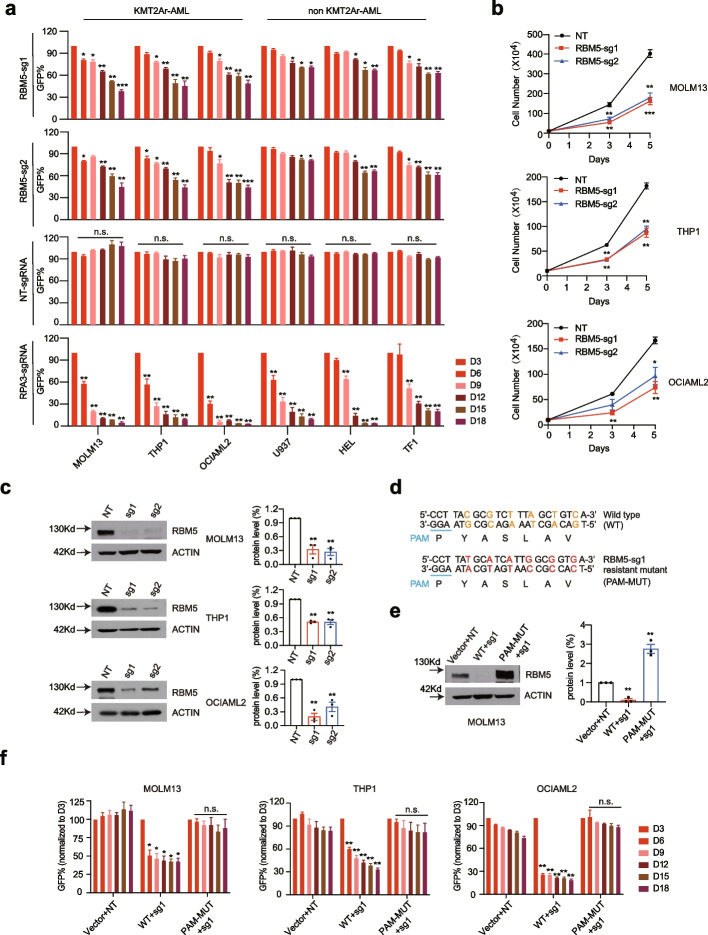
Disruption of RBM5 delays the growth of leukemia cells in vitro. **a** Competitive proliferation assay (CPA) was conducted in Cas9 stably expressed AML cells lines, including MOLM13, THP1, OCIAML2, U937, HEL, and TF1 after transduced with GFP reporter-based lentiviral sgRNAs (NT, RPA3, RBM5-sg1, RBM5-sg2) at about ~ 50% efficiency. The GFP% was quantified at days 3, 6, 9, 12, 15, and 18 by flow cytometry to evaluate the growth disadvantage. The guide RNA targeting the survival essential gene RPA3 was included as a positive control, and the guide RNA targeting the non-target (NT) gene was included as a negative control. Data shown are means ± SEM from three independent experiments. *P < 0.05, **P < 0.01, ***P < 0.001, unpaired Student’s *t*-test. **b** The proliferation ability of AML cells was monitored by cell counting assay in MOLM13, THP1, and OCIAML2 with stably expressed Cas9 after transduced by two respective sgRNAs targeting RBM5 (RBM5-sg1, sg2) and one non-targeting sgRNA. The guide RNA targeting non-target gene (NT) was used as a negative control. Data shown are means ± SEM from three independent experiments. *P < 0.05, **P < 0.01, ***P < 0.001, unpaired Student’s *t*-test. **c** Immunoblotting of RBM5 in RBM5 sgRNAs targeted cells. The bands were scanned and statistically analyzed. Data shown are means ± SEM from three independent experiments. *P < 0.05, **P < 0.01, ***P < 0.001, unpaired Student’s *t*-test. The β-ACTIN was used as a reference. **d** Schematic diagram of the sgRNA-resistant cDNA mutagenesis. The 22 bp DNA sequence and corresponding amino acids close to the sgRNA PAM region (blue) in RBM5 wild-type (WT) and RBM5-sg1-resistant mutant (PAM-MUT) cDNA are shown with the non-sensed mutated nucleotides highlighted in different colors (red and orange). **e** Immunoblotting was conducted by infecting MOLM13 cells overexpressing ectopic Venus empty vector, RBM5-wild-type cDNA (WT), RBM5-sgRNA1-resistant mutant cDNA (PAM-MUT) with lentiviral-sgRNAs against non-target gene (NT), and RBM5 (RBM5-sg1), and the bands were scanned and statistically analyzed. Data shown are means ± SEM from three independent experiments. *P < 0.05, **P < 0.01, ***P < 0.001, unpaired Student’s *t*-test. The β-ACTIN was used as a reference. **f** Competition-based proliferation assays comparing the impact of sgRNAs on MOLM13, THP1, and OCIAML2 cell fitness, in the context of co-transduction with Venus empty vector control, RBM5-wild-type cDNA (WT), RBM5-sgRNA1-resistant mutant cDNA (PAM-MUT) (all monitored by Venus reporter). Data shown are means ± SEM from three independent experiments. *P < 0.05, **P < 0.01, ***P < 0.001, unpaired Student’s *t*-test

Inspired by the CRISPR screen and expression correlation evidence in AML pathogenesis, we reasoned the regulatory function of the RBM5/HOXA9 circuitry in the AML context could be verified by loss-of-function studies. We observed that RBM5 is essential in multiple leukemia cell lines, including both KMT2A-r and non-KMT2A-r-mutated cell lines, from the public DepMap dataset (Additional file 1: Fig. S2a). We then used the CRISPR/Cas9-mediated gene knockout strategy to disrupt RBM5 in multiple AML cell lines (MOLM13, THP1, OCIAML2, U937, HEL, TF1). We first designed two individual sgRNAs against different exons of RBM5. A competitive proliferation assay (CPA) was conducted to exhibit that disruption of RBM5 impaired the cell growth of three AML lines (MOLM13, THP1, OCIAML2) but had a mild effect on the three other cell lines (U937, HEL, TF1) (Fig. 2a). In contrast, a sgRNA targeting the pan-essential gene RPA3 impaired the proliferation of all six cell lines with similar efficiency, while the non-targeting sgRNA did not affect cell proliferation (Fig. 2a). Consistently, cell counting assay recapitulated the same observation that cells showed significantly delayed growth targeted with two sgRNAs against the coding sequence of RBM5 in MOLM13, THP1, and OCIAML2 cell lines but with a mild effect in U937, HEL, and TF1 cell lines (Fig. 2b; Additional file 1: Fig. S2b). To exclude the phenotype variation due to the various knockout efficiency, we confirmed the significant reduction of RBM5 at both mRNA and protein levels by the two individual sgRNAs in all the tested AML cell lines (Fig. 2c; Additional file 1: Fig. S2c, d). It was commonly observed that CRISPR/Cas9-mediated gene knockout technology could induce an off-target effect due to the sequence mismatch [34]. To exclude the off-target impact in our study, we sought to test if RBM5 overexpression could restore the growth defects. Thus, we constructed both the wild-type form of human RBM5 cDNA and the validated sgRNA-resistant mutant form that contains several mismatches but synonymous mutations (Fig. 2d). We confirmed that the sgRNA-resistant form did maintain a similar RBM5 expression when targeted by RBM5 sgRNA (Fig. 2e; Additional file 1: Fig. S2e). Of note, we observed that the growth defect of RBM5 knockout cells could be fully rescued by the exogenous expression of sgRNA-resistant RBM5 cDNA but not the wild type in all three leukemia cell lines (MOLM13, THP1, and OCIAML2) (Fig. 2f). Those results strongly suggest that RBM5 is indeed required to grow leukemia cells.

### *Suppression of RBM5 impairs *in vivo* myeloid leukemia engraftment*

**Fig. 3:**
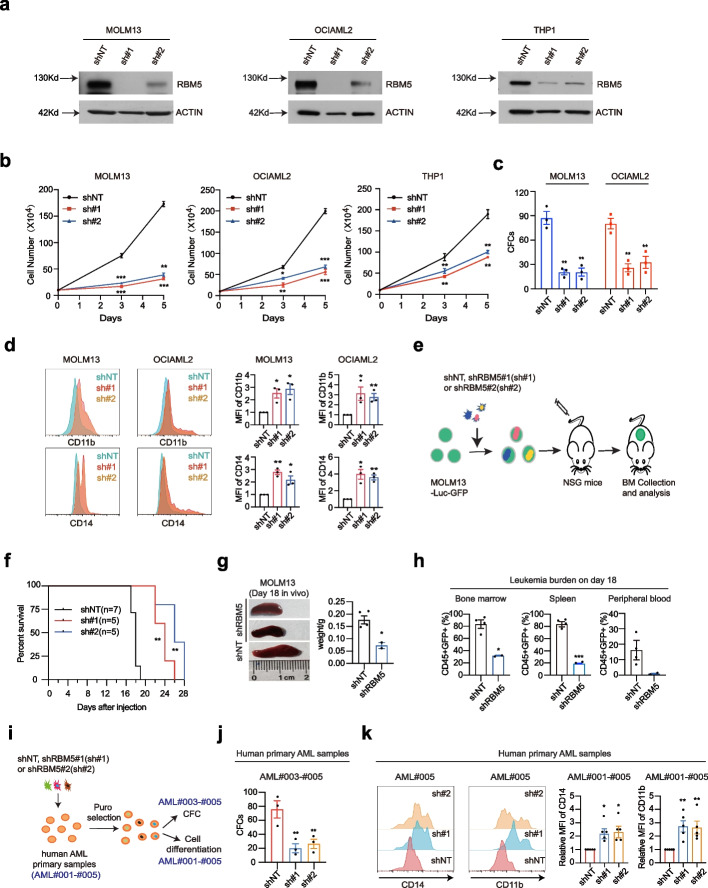
RBM5 knockdown impairs in vivo myeloid leukemia engraftment. **a** Western blot of RBM5 expression in MOLM13, OCIAML2, and THP1 cells after transduced with shNT (non-targeting control), sh#1 (shRBM5#1), and sh#2 (shRBM5#2) 6-day post-viral transduction. The β-ACTIN was used as a reference. **b** Cell counting assay was conducted on the sh#1, sh#2, and shNT targeted MOLM13, OCIAML2, and THP1 to monitor the ability of proliferation of AML cells. shNT was used as a negative control. Data shown are means ± SEM from three independent experiments. *P < 0.05, **P < 0.01, ***P < 0.001, unpaired Student’s *t*-test. **c** Count of colonies formed by 300 MOLM13 and 500 OCIAML2 cells after shNT, sh#1, and sh#2 transduction. Data shown are means ± SEM from three independent experiments. **P < 0.01, unpaired Student’s *t*-test. **d** Surface expression of CD11b and CD14 after lentiviral transduction of shRNA against control shNT and shRNAs targeting RBM5 (sh#1 and sh#2) in MOLM13 and OCIAML2 cells. MFI, mean fluorescence intensity. *P < 0.05, **P < 0.01, unpaired Student’s *t*-test. **e** Schematic of in vivo transplantation of MOLM13-Luc-GFP cells infected with control shNT and shRNAs targeting RBM5 (sh#1 and sh#2). **f** Kaplan–Meier survival curves of recipient mice transplanted with MOLM13 cells transduced with shNT (*n* = 7), sh#1 (*n* = 5), and sh#2 (*n* = 5). The *P*-value was determined by a log-rank Mantel-Cox test. **P < 0.01. **g** The spleens of mice in the shNT (*n* = 4) and shRBM5 (*n* = 2, merged sh#1 and sh#2) groups were photographed and weighed 18-day post-transplantation. *P < 0.05, unpaired Student’s *t*-test. **h** Flow cytometry analysis of the percentage of human CD45^+^ and GFP^+^ leukemia cells in bone marrow, spleen, and peripheral blood of recipient mice in the shNT (*n* = 4) and shRBM5 (*n* = 2) groups sacrificed after 18-day post-transplantation. Statistical analysis (*P*-value) was performed using an unpaired Student’s *t*-test. *P < 0.05, ***P < 0.001. **i** Schematic diagram of the AML primary sample for CFC and cell differentiation assay. **j** Count of colonies formed by human primary AML cells after shNT, sh#1(shRBM5#1), and sh#2(shRBM5#2) transduction. **P < 0.01, unpaired Student’s *t*-test. **k** Representative flow cytometry analysis of myeloid differentiation marker (CD14 and CD11b), in which AML primary samples were treated with lentiviral transduction of shRNA against control gene (shNT) and shRNAs targeting RBM5 (sh#1 and sh#2). MFI, mean fluorescence intensity. *P < 0.05, **P < 0.01, unpaired Student’s *t*-test

To further examine the functional requirement of RBM5, we utilized an alternative loss-of-function approach, RNA interference (RNAi), to target RBM5 mRNA in AML cells as a complementary approach to the DNA targeting strategy as CRISPR/Cas9. Two independent shRNAs (RBM5: sh#1 and sh#2) had been validated to efficiently suppress RBM5 expression in three validated AML cell lines and significantly impaired cell growth and reduced colony numbers (Fig. 3a–c). Moreover, RBM5 suppression also triggered notable myeloid differentiation in AML cells, as demonstrated by the increased expression of myeloid maturation markers CD11b and CD14 in both mRNA and protein levels, together with morphologic features of differentiation (Fig. 3d; Additional file 1: Fig. S3a, b). Consistently, RBM5 suppression also induced cell apoptosis in the targeted cell lines (Additional file 1: Fig. S3c). We obtained similar results for myeloid differentiation and cell apoptosis by CRISPR/Cas9-mediated knockout in AML cells (Additional file 1: Fig. S3d and e). Next, we sought to test the role of RBM5 on AML progression in vivo by xenograft studies. We transduced luciferase-GFP-labeled MOLM13 cells with two individual shRNA targeting RBM5 and a non-targeting control, isolated the successfully transduced cells by puromycin selection, and injected them into NSG mice (Fig. 3e). Notably, RBM5 shRNA-treated cells significantly improved the overall survival of the mice compared to the control cells (Fig. 3f). Strikingly, RBM5 shRNA-treated animals exhibited much less splenomegaly and reduced leukemia cell infiltration in bone marrow, spleen, and peripheral blood compared to the wild-type control (Fig. 3f–h; Additional file 1: Fig. S3f). Together, those results suggest that RBM5 is required for AML progression in vivo.

In addition to cell lines validation, we then conducted the functional validation assay in human AML patient-derived primary cells (Fig. 3i; Additional file 1: Fig. S3g). Importantly, RBM5 knockdown by shRNA in primary AML cells significantly reduced colony numbers (Fig. 3j). Moreover, RBM5 suppression also induced myeloid differentiation in primary AML cells, as demonstrated by the maturation markers CD11b and CD14 (Fig. 3k). To further confirm the dependency of RBM5 in normal hematopoiesis, we validated the effect of RBM5 reduction on CD34^+^ primary hematopoietic stem and progenitor cells from three individual normal donors. Strikingly, suppression of RBM5 slightly reduced myeloid differentiation markers and did not affect the formation of myeloid or mixed-lineage colonies after 14 days of in vitro culture (Additional file 1: Fig. S4a**–**c). Moreover, we obtained similar results after electroporating the Cas9*-RBM5* sgRNA#1 ribonucleoprotein (RNP) complex into peripheral blood CD34^+^ HSPCs from three normal donors. RBM5 deletion did not alter the myeloid differentiation or the formation of myeloid or mixed-lineage colonies (Additional file 1: Fig. S4d**–**f). Overall, our data support that RBM5 maintains leukemia cell survival in both cell lines and primary samples while not required for normal human myeloid proliferation and differentiation. Nevertheless, the effect of RBM5 in normal hematopoiesis by other in vivo models needs to be further evaluated comprehensively.

### RRM and zinc finger domains of RBM5 are essential in AML

**Fig. 4:**
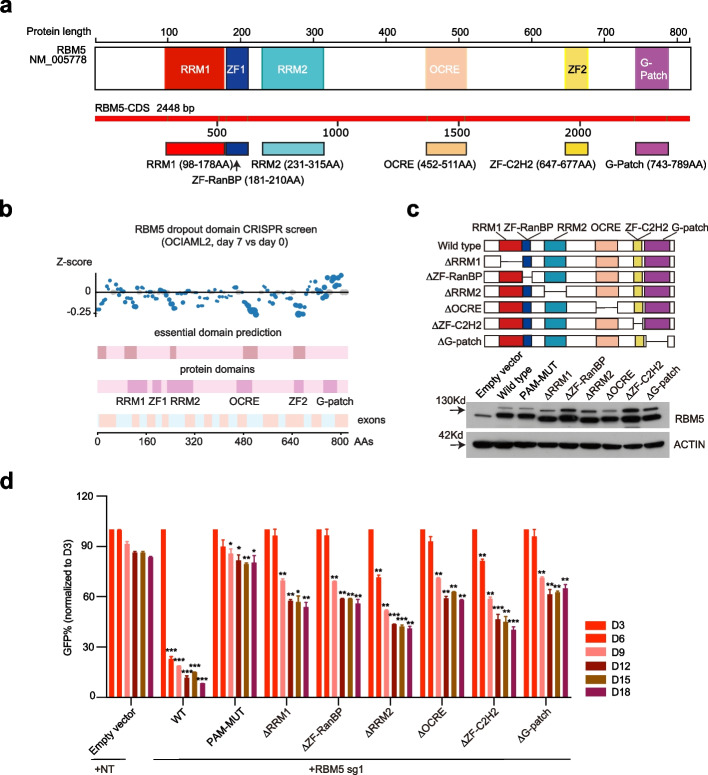
RRM and zinc finger domains of RBM5 are essential in AML. **a** RBM5 protein structure. The illustration shows the major canonical functional domains in the human RBM5 protein. The amino acid distance spanning each functional domain is indicated in the colorful pane below. RNA recognition domain (RRM), zinc-finger domain (ZF), octamer repeat (OCRE) domain, glycine-rich region (G-patch) domain. **b** CRISPR dropout screen by tilling the sgRNAs targeting the RBM5 exons in OCIAML2 cells at day 7. The known protein domains (including RRM1, RRM2, ZF-RanBP, ZF-C2H2, OCRE, and G-patch) were highlighted in purple boxes, while the predicted essential domains were highlighted in brown boxes. The Z-score was calculated based on the average count of all sgRNAs targeting a 10-kb window in the genome. **c** Western blot analysis was conducted in OCIAML2 cells transduced with RBM5 cDNA wild-type (WT) or individual mutant (upper panel) to validate the expression of RBM5 (lower panel). The β-ACTIN was used as a reference. **d** Competition-based proliferation assay in OCIAML2 expressing the indicated individual RBM5 truncated mutant cDNAs, RBM5-wild-type cDNA (WT), RBM5-sgRNA1-resistant mutant cDNA (PAM-MUT), and empty vector (all monitored by Venus reporter), followed by transduction with RBM5 sg1 or non-targeted sgRNA (NT). Data shown are means ± SEM from three independent experiments. *P < 0.05, **P < 0.01, unpaired Student’s *t*-test

As predicted by AlphaFold [35] and relevant structure studies [36], the human RBM5 contains two RNA recognition motif (RRM) (RRM1 and RRM2), two zinc-finger (ZF) domains (ZF-RanBP and ZF-C2H2), octamer repeat (OCRE) domain, and glycine-rich region (G-patch) domain (Fig. 4a; Additional file 1: Fig. S5a). Although the RRM and ZF domains are thought to modulate distinct sets of mRNAs [37, 38], and other domains (e.g., G-Patch) may help to coordinate its activity during RNA splicing, the functional roles of each RNA-binding domain, the cell signaling functions of the ZF domains, or the specificity of those effects remain to be determined in AML [39]. We hypothesized that specific protein domains of RBM5 are responsible for AML pathogenesis. To test this hypothesis, we employed a CRISPR/Cas9 domain screen to identify essential RBM5 protein domains in AML. We designed a tilling 186 sgRNA array with low off-target scores to target each RBM5 coding exon and performed a dropout screen in the Cas9-expressing OCIAML2 cells. These results revealed significant depletion of sgRNAs that targeted RRM1, RRM2, OCRE, and Zf-C2H2, whereas ZF-RanBP exhibited lesser negative selection after 1 week of expansion (Fig. 4b; Additional file 4: Table S3). Moreover, we constructed RBM5 cDNA mutants by truncating six individual known domains and further confirmed the functional role of different domains of RBM5 in leukemia cell growth. Of note, we observed that all six domain mutants failed to rescue the growth defect of RBM5 knock-out cells, especially with the most significant effect in the RRM2 and ZF-C2H2 domains (Fig. 4c, d). These results suggest that RBM5 relies on both RRM and zinc-finger domains and supports a critical RBM5 dependency in AML.

### Identification of RBM5 downstream target genes in AML

**Fig. 5:**
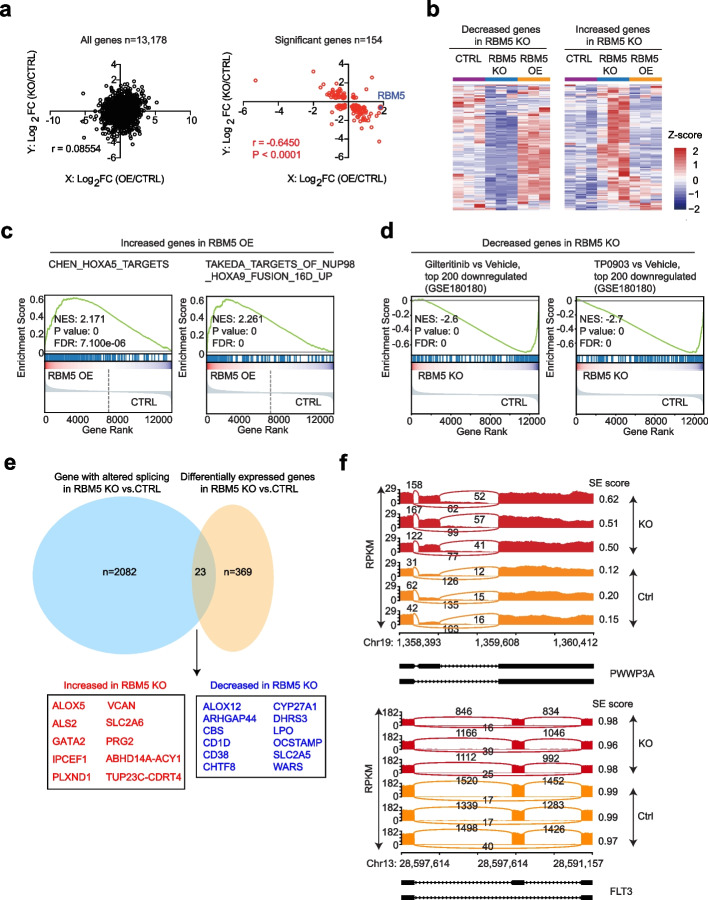
Identification of RBM5 downstream target genes in AML. **a** The transcriptome analysis in three groups of MOLM13 cells, including the CTRL group (control cells with NT sgRNA treated), RBM5 KO group (RBM5 knockout cells with RBM5-sg1 treated), and RBM5 OE group (RBM5 overexpressed cells by lentiviral RBM5-P2A-venus vector). Each group was performed with three biological replicates. Integrated scatter plot analysis for all genes from the RNA-seq dataset by comparing RBM5 OE vs. CTRL and RBM5 KO vs. CTRL in MOLM13 cells (left panel) (*n* = 13,178). The graph on the right panel shows the significant genes (*n* = 154). False discovery rate [FDR] < 0.05. **b** Heat map showing filtered genes whose expression is significantly differentially expressed after RBM5 loss in three groups of MOLM13 cells, including the CTRL, RBM5 KO, and RBM5 OE. **c** Leading edge plot from gene enrichment analysis (GSEA) showing the enrichment of indicated pathway for genes in RBM5 OE compared to control. Enrichment scores, *P*-values, and FDR (false-discovery rate) are calculated as shown in the plot. **d** Leading edge plot from gene enrichment analysis (GSEA) showing the enrichment of indicated pathway for the decreased genes in RBM5 KO cells relative to control. Enrichment scores, *P*-values, and FDR (false-discovery rate) are calculated as shown in the plot. **e** Venn diagram showing the overlap between genes differentially expressed and genes undergoing altered splicing events after RBM5 knockout (from RNA-seq). **f** Sashimi plots the example gene *PWWP3A* and *FLT3* in RBM5 KO and CTRL group in MOLM13 cells

Although we have confirmed that RBM5 is required for the survival of acute myeloid leukemia cells, it is equally important to reveal the downstream regulation mechanism of RBM5. Based on the limited information from the literature [27, 40, 41], RBM5 may positively and negatively regulate apoptosis via the alternative splicing of different genes, such as FAS and CASP2/caspase2. To comprehensively understand the downstream target genes of RBM5 in AML, we performed genome-wide transcriptome analysis by RNA-seq in the leukemia cell line MOLM13 with both RBM5 knockout (RBM5 KO) and RBM5 overexpression (RBM5 OE) compared to wild-type controls. Principal component analysis (PCA) was utilized to compare the whole transcriptome at different status. Interestingly, both the RBM5 OE and the KO groups were separated from the control groups, suggesting that either gain or loss of RBM5 could result in notable transcriptome change (Additional file 1: Fig. S6a). We next performed the differential gene expression analysis in different comparisons, including RBM5 KO versus control and RBM5 OE versus control. Using the consistent and stringent cutoff (*FDR* < 0.05, Log_2_(fold change) > 1), 201 increased genes and 169 decreased genes were identified in RBM5 KO, while 618 increased genes and 210 decreased genes were identified in RBM5 OE relative to control (Additional file 1: Fig. S6b; Additional file 5: Table S4). To further uncover the relationship of the downstream differentially expressed genes (DEGs) between RBM5 OE and RBM5 KO, we performed the Pearson’s correlation analysis for the total genes and the DEGs. Notably, a strong and significant negative correlation for the DEGs but not all genes was observed between RBM5 OE and RBM5 KO (Fig. 5a). The heatmap exhibits a similar correlation pattern (Fig. 5b; Additional file 1: Fig. S6c). Next, we performed Gene set enrichment analysis (GSEA) in RBM5 OE and RBM5 KO groups compared to the negative control. Notably, highly significant enrichment of the HOXA5 targets and the target genes of NUP98-HOXA9 fusion for the increased genes in RBM5 OE, further suggesting a correlation between RBM5 and HOXA9 (Fig. 5c). Moreover, consistent with the survival defect after RBM5 loss, the GSEA revealed significant enrichment of genes involved in FLT3 inhibitor treatment, including gilteritinib and TP0903 responsive targets in the set of decreased genes in RBM5 KO (Fig. 5d). The transcriptome analysis of both RBM5 OE and RBM5 KO provides a direct link with AML pathogenesis.

RBM5 was reported as a component of the spliceosome A complex and regulated the alternative splicing of many mRNAs [27]. We reasoned that RBM5 might directly regulate a set of genes’ alternative splicing in AML. To uncover this, we performed a comprehensive RNA-seq analysis and identified differential splicing events in the RBM5 OE and KO groups relative to the control. Interestingly, the significant differential splicing events in both groups were highly correlated (Additional file 1: Fig. S6d; Additional file 6: Table S5), suggesting that both gain and loss of RBM5 lead to the dysregulation of RNA splicing. Further, we observed that RBM5 is involved in different types of alternative splicing events, including cassette exon (CE), alternative 5′ splice site (A5SS), alternative 3′ splice site (A3SS), mutually exclusive exon (MXE), and retained intron (RI), with the CE being the most affected in RBM5 KO (Additional file 1: Fig. S6e). We next asked whether the genes involved in the different types of splicing events were affected in the steady state of the transcriptional level after RBM5 KO. Of note, most of the genes associated with dysregulated RNA splicing remained unchanged in the transcription level and rarely overlapped with the differential expressed genes after RBM5 loss** (**Fig. 5e; Additional file 1: Fig. S6f). In addition, although a global splicing change profiling was observed, including *PWWP3A*, the splicing events of survival essential genes in AML such as *FLT3,* remained unchanged (Fig. 5f; Additional file 7: Table S5). In addition, we analyzed the splicing events for the HOXA9-related downstream targets from the GSEA dataset. The splicing events of HOXA9 targets after RBM5 loss in AML largely remained unchanged (Additional file 1: Fig. S6g). Thus, our data suggests that, although RNA splicing defect was observed in RBM5 knockout cells, the major downstream target genes essential for AML pathogenesis did not change in the level of RNA splicing.

### HOXA9 is a functional target gene of RBM5 in AML

**Fig. 6:**
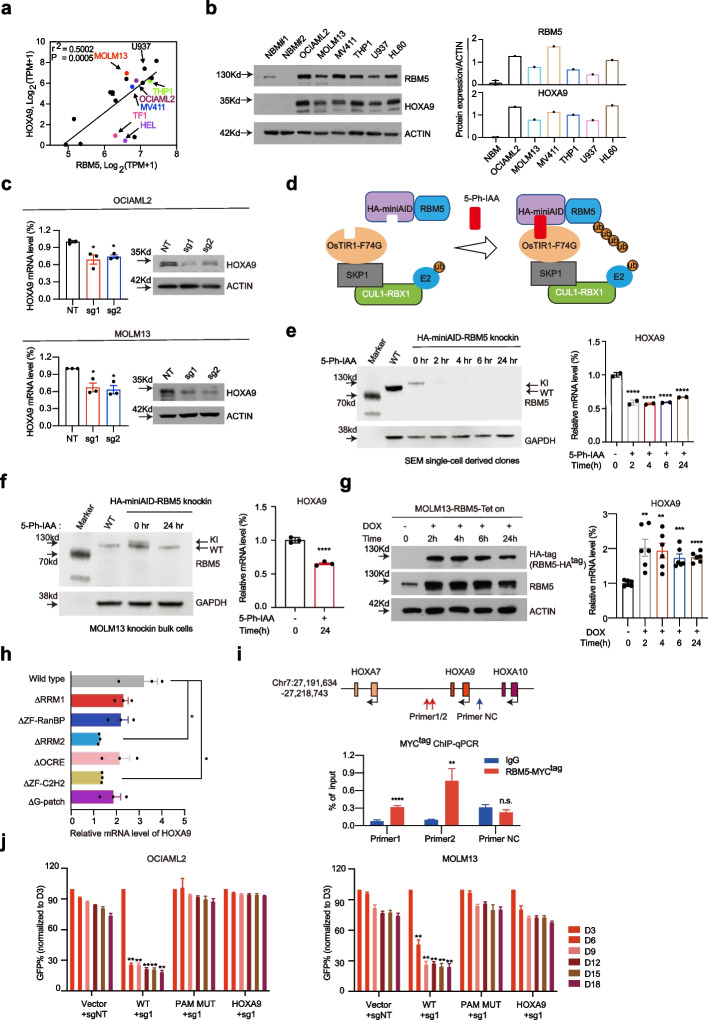
HOXA9 is a functional target gene of RBM5 in AML. **a** Scatter plot analysis comparing *RBM5* mRNA levels and *HOXA9* mRNA levels across acute myeloid leukemia cell lines (data was obtained from the Depmap). *P*-value is calculated by linear regression. **b** Immunoblotting of RBM5 and HOXA9 in various leukemia cell lines and normal bone marrow (NBM) samples (left panel). The bar chart displays the quantitative protein levels of RBM5 and HOXA9 in various cell lines depicted in the left panel (right panel). **c** Real-time-qPCR and immunoblotting analysis was conducted on the RBM5-sg1, RBM5-sg2, and sgNT targeted MOLM13 and OCIAML2 to monitor the expression of *HOXA9*. Data shown are means ± SEM from three independent experiments. *P < 0.05, unpaired Student’s *t*-test. **d** Diagram of auxin-induced degron (AID) system to degrade endogenous RBM5. Endogenous RBM5 N-terminus AID knock-in KMT2A-r leukemia lines (SEM homozygous clones and MOLM13 bulk cells) were followed by constitutively expressing OsTIR1(F74G). With the presence of small molecule 5-Ph-IAA, the OsTIR1(F74G), as a substrate receptor in Skp1, Cullin, and F-box (SCF) complex, could target and mediate specific AID-tagged RBM5 proteins for ubiquitination and degradation. **e** The protein level of endogenous HA-miniAID-RBM5 of SEM homozygous clones can be acutely degraded with 5-Ph-IAA through degron-mediated proteasome degradation after 2-h post-treatment (left panel). HOXA9 mRNA level was significantly reduced after 2 h, 4 h, 6 h, and 24 h of treatment in SEM cells (right panel). **f** After 24 h of treatment with 5-Ph-IAA in HA-miniAID-RBM5 knock-in MOLM13 cells, the protein level of HA-miniAID-RBM5 decreased (left panel), and there was also a significant reduction in HOXA9 mRNA level (right panel). **g** The doxycycline-Tet-On system for ectopic overexpression of RBM5-HA in the MOLM13 cell line, after a 2-h treatment with doxycycline, a significant upregulation in HOXA9 mRNA levels was observed (right panel), accompanied by an increase in RBM5 expression (left panel). **h** Real-time-qPCR analysis was conducted in OCIAML2 cells transduced with RBM5 cDNA wild-type (WT) or six individual truncated mutants to validate the expression of HOXA9. Data shown are means ± SEM from three independent experiments. *P < 0.05, unpaired Student’s *t*-test. **i** ChIP-qPCR with anti-MYC tag antibody near H3K4me3-bound HOXA9 downstream region (Chr7:27,199,969–27,200,853) in OCIAML2-RBM5-MYC^tag^ cells (*n* = 3). The red arrows indicate the primer 1 and primer 2 located position, and the blue arrow indicates the negative control (NC) primer located position, where there is no H3K4me3 signal. Statistical analysis (*P*-value) was performed using an unpaired Student’s *t*-test. All error bars represent mean ± SEM. **j** Rescued competitive proliferation assay was conducted by infecting OCIAML2-Cas9 and MOLM13-Cas9 cells overexpressing ectopic RBM5-wild-type cDNA (WT), RBM5-sgRNA1-resistant mutant cDNA (PAM-MUT), and HOXA9 cDNA (linked to Venus reporter) with lentiviral-sgRNAs against non-target (sgNT) and RBM5 (RBM5-sg1) at about 50% efficiency (all monitored by Venus reporter). Data shown are means ± SEM from three independent experiments. **P < 0.01, unpaired Student’s *t*-test

Given the significant enrichment of HOXA9-related downstream targets from the GSEA analysis (Fig. 5c) and the rationale of the reporter-based screen, we proposed that HOXA9 might serve as a functional and direct target of RBM5 in AML. Indeed, we observed that RBM5 mRNA expression positively correlated with HOXA9 in multiple AML cell lines in the collection of DepMap (Fig. 6a). We sought to validate the RBM5 and HOXA9 expression levels in multiple human AML cell lines and confirmed their regulation correlation (Fig. 6b). Of note, the reduction of RBM5 in multiple cell lines results in a significant decrease of endogenous HOXA9 expression including HOXA9-mCherry reporter cells (Fig. 6c; Additional file 1: Fig. S7a–c). Due to the technique limitations, we also realized that CRISPR-mediated knockout and shRNA-mediated knockdown approaches take time to achieve the loss-of-function readout, leading to the possible secondary effect. To mitigate this potential problem, we sought to establish an acute protein degradation system to target RBM5. We reasoned that the direct regulation of HOXA9 gene transcription would occur in an acute manner. Therefore, we utilized the auxin-induced degron (AID) system and generated two endogenous RBM5 N-terminus AID knock-in KMT2A-r leukemia lines (SEM homozygous clones and MOLM13 bulk cells). Those cell lines were then transduced with lentiviral vectors constitutively expressing OsTIR1(F74G) [42, 43]. With the presence of small molecule 5-Ph-IAA, the OsTIR1(F74G), as a substrate receptor in Skp1, cullin, and F-box (SCF) complex, could target and mediate specific AID-tagged proteins for ubiquitination and degradation (Fig. 6d). Of note, after 2-h posttreatment, the immunoblotting analysis showed that the AID-RBM5 fusion protein was undetectable. Consequently, the HOXA9 mRNA level was significantly reduced in two SEM knock-in clones after 2 h of treatment. It remained low until 24 h (Fig. 6e). Similar effect was also observed after 24 h of 5-Ph-IAA treatment in MOLM13 knock-in bulk populations (Fig. 6f), while conversely the overexpression of RBM5 increased the HOXA9 expression with no change in the mRNA splicing (Additional file 1: Fig. S7d–f). Such effects were also observed by overexpressing an exogenous RBM5-N terminus AID fusion protein (Additional file 1: Fig. S7g). Strikingly, upon the addition of 5-Ph-IAA in the presence of OsTIR1 (F74G), a significant reduction of HOXA9 mRNA was observed after 2 h of treatment and after that (Additional file 1: Fig. S7g). To further test this effect in an acute manner, we utilized an inducible overexpression system with the TRE3G promoter in the MOLM13 cell line. With the treatment of doxycycline for 2 h and after that, we found significant upregulation of HOXA9 mRNA level accompanied by the increase of RBM5 expression (Fig. 6g). Taken together, those results indicate that RBM5 directly activated the HOXA9 gene transcription.

Moreover, by constructing RBM5 cDNA mutants via truncating six individual known domains, we observed that all domain mutants exhibited a milder effect on the HOXA9 genes’ activation than the wild-type setting, especially with the most significant extent in the RRM2 and ZF-C2H2 domains (Fig. 6h). Given the importance of DNA-binding domains, we hypothesized that RBM5 may directly bind to the *HOXA9* gene locus. To test this, we performed an anti-MYC tag antibody ChIP (chromatin immunoprecipitation) to analyze the chromatin occupancy of exogenous RBM5-MYC expressed in OCIAML2 leukemia cells. Importantly, we observed significant enrichment of RBM5-MYC on the downstream region of HOXA9, together with active transcription mark H3K4me3 (Fig. 6i; Additional file 1: Fig. S7h). To further examine the AML survival dependency on RBM5/HOXA9 axis, we tested whether ectopic expression of HOXA9 could rescue the growth defect upon RBM5 loss. Notably, the overexpression of HOXA9 significantly restored the cell expansion in the RBM5 sgRNA-treated leukemia cells, similar to the extent of RBM5 overexpression (Fig. 6j; Additional file 1: Fig. S7i). Thus, these results indicate that HOXA9 is a crucial functional downstream target of RBM5 in AML growth.

### HOXA9 partially restores the gene transcriptional defect in RBM5 null leukemia cells

**Fig. 7:**
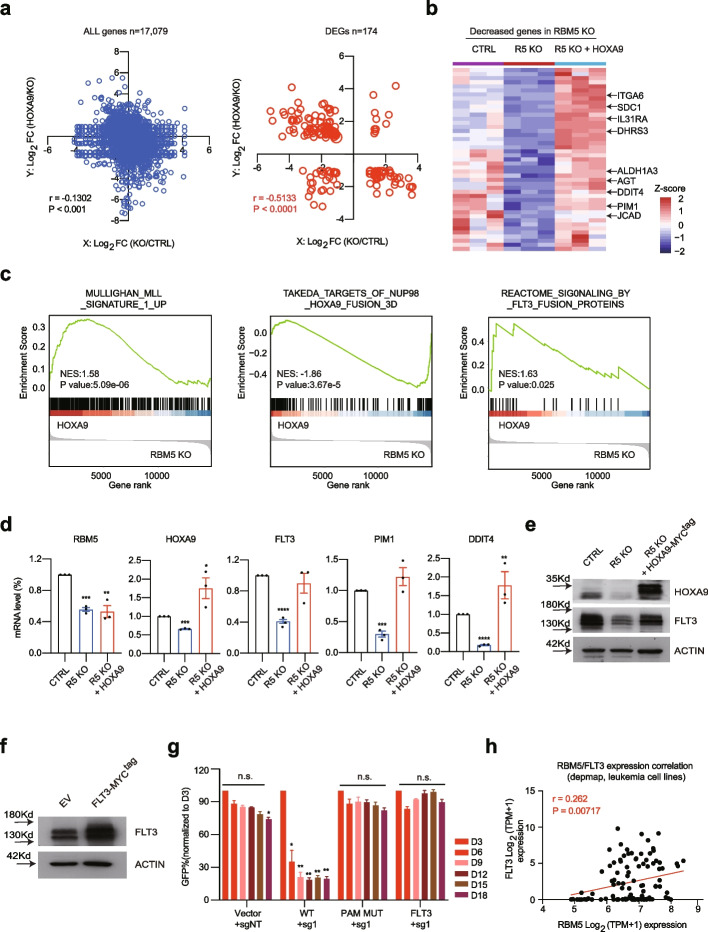
HOXA9 partially restores the gene transcriptional defect in RBM5 null leukemia cells. **a** Integrated scatter plot analysis for all genes (*P* < 0.05) from the RNA-seq dataset by comparing HOXA9 vs. RBM5 KO and RBM5 KO vs. CTRL in MOLM13 cells (left panel) (*n* = 17,079). The graph at the right shows the differentially expressed genes (DEG) in RBM5 KO vs. CTRL (*n* = 174). False discovery rate [FDR] < 0.05. Pearson r and *P*-value are calculated by correlation. **b** Heat map of RNA-seq analysis shows the decreased genes after RBM5 loss respectively in CTRL, RBM5 KO, and RBM5 KO with HOXA9 overexpression groups. **c** Enrichment of target genes involved in MLL signature genes, NUP98-HOXA9 and FLT3 targets for the increased genes in RBM5 KO cells with HOXA9 overexpression relative to control, as shown by GSEA. **d**. Real-time-qPCR analysis was performed on samples of CTRL, RBM5 KO (R5 KO), and RBM5 KO MOLM13 cells with HOXA9 cDNA transduction (R5 KO + HOXA9) to validate the expression of *RBM5*, *HOXA9*, *FLT3*, *PIM1*, and *DDIT4* genes. Data shown are means ± SEM from three independent experiments. ***P < 0.001, ****P < 0.0001, unpaired Student’s *t*-test. **e** Western blot of HOXA9 and FLT3 were performed on samples of CTRL, R5 KO, and RBM5 KO MOLM13 cells with HOXA9 cDNA transduction (R5 KO + HOXA9). The β-ACTIN was used as a reference. **f** Immunoblotting was conducted by infecting OCIAML2 cells overexpressing ectopic Venus empty vector and FLT3 cDNA. The β-ACTIN was used as a reference. **g** Rescued competitive proliferation assay was conducted by infecting OCIAML2-Cas9 cells overexpressing ectopic RBM5-wild-type cDNA (WT), RBM5-sgRNA1-resistant mutant cDNA (PAM-MUT), and FLT3 cDNA (linked to Venus reporter) with lentiviral-sgRNAs against non-target (sgNT) and RBM5 (RBM5-sg1) at about 50% efficiency (all monitored by Venus reporter). Data shown are means ± SEM from three independent experiments. **P < 0.01, unpaired Student’s *t*-test. **h** Scatter plot analysis comparing *RBM5* mRNA levels and *FLT3* mRNA levels across leukemia cell lines (data was obtained from the DepMap). *P*-value is calculated by linear regression

Given the ectopic expression of HOXA9 rescues the growth defect upon RBM5 loss, we reasoned that HOXA9 should restore the expression of some key essential genes that are reduced after RBM5 loss. To further explore the underlying molecular mechanism, we performed transcriptome analysis with and without HOXA9 overexpression (HOXA9 OE) in the RBM5 knockout leukemia cells. To examine the relationship of the downstream differentially expressed genes (DEGs) between HOXA9 OE and RBM5 KO, we performed the Pearson’s correlation analysis for the total genes and the DEGs. Notably, a strong and significant negative correlation for the DEGs relative to all genes was observed between HOXA9 OE and RBM5 KO (Fig. 7a; Additional file 7: Table S6). Interestingly, the focused analysis of DEGs after RBM5 loss demonstrated that HOXA9 could partially restore the expression of downstream targets compared to RBM5 knockout cells (Fig. 7b**)**. Moreover, the rank-based GSEA of differentially expressed mRNAs after HOXA9 overexpression in RBM5 KO cells exhibited significant enrichment for the targets of NUP98-HOXA9, MLL signature genes, and FLT3 targets, consistent with the survival effect with HOXA9 overexpression (Fig. 7c**)**.

FLT3, a single transmembrane receptor among the type II receptor tyrosine kinase family, is well known to play a crucial role in hematopoiesis [44]. Aberrant activation of FLT3 due to mutation or epigenetic deregulation can be oncogenic, contributing to the pathogenesis of myeloid leukemia [45, 46]. Significantly, FLT3 inhibitors such as gilteritinib have been widely explored in AML therapy [47]. The top two enriched GSEA pathways in the decreased genes after RBM5 KO are associated with FLT3 inhibitors, including gilteritinib and TP0903 (Fig. 5d), suggesting that the FLT3 gene might contribute to AML survival after the loss of RBM5. To test this hypothesis, we sought to validate how the FLT3 and its responsive genes were changed due to the loss of RBM5 and after HOXA9 overexpression. Strikingly, the FLT3 and downstream effector genes of FLT3, as exemplified by *PIM1 and DDIT4*, were consistently reduced after RBM5 suppression and could be rescued by HOXA9 overexpression (Fig. 7d). In addition, the protein level of FLT3 was consistently reduced after suppression of RBM5 and restored after HOXA9 overexpression (Fig. 7e). More importantly, the overexpression of FLT3 significantly restored the cell expansion in the RBM5 sgRNA-treated leukemia cells, similar to the extent of both RBM5 and HOXA9 overexpression (Fig. 7f, g). Next, we explored the correlation between RBM5 and FLT3 mRNA levels in a broad panel of AML cell lines and confirmed a significant correlation (Fig. 7h). Taken together, these results strongly suggest that RBM5/HOXA9 is critical for the FLT3 gene expression and may participate in FLT3 responsive signal transduction in AML.

## Discussion

**Fig. 8:**
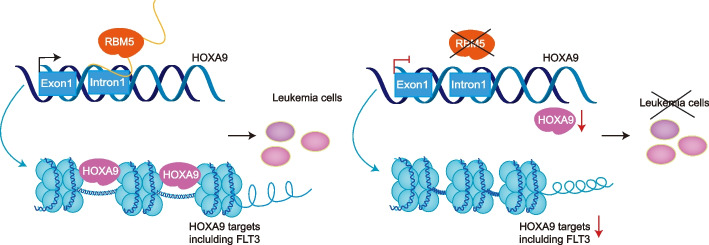
Model for the new regulatory axis RBM5-HOXA9 in AML. RBM5 directly regulates the transcriptional activity of the HOXA9 locus, which maintains proper downstream signaling transduction, including the FLT3-responsive targets in acute myeloid leukemia cells

The RNA-binding protein RBM5 has been implicated as a tumor suppressor in multiple types of solid cancers, such as lung cancer [26]. However, its function as an oncogenic protein or regulatory role in leukemias has not yet been fully understood. Here, using *HOXA9*-reporter-based genome-wide CRISPR/Cas9 screens, we identified that RBM5 is a positive regulator for maintaining HOXA9 expression and acute myeloid leukemia survival. Furthermore, we showed that both the RNA-binding and DNA-binding domains are required for RBM5’s role in leukemia. And importantly, the known driver gene of AML, HOXA9, was verified as a downstream target of RBM5, suggesting a new regulatory axis in leukemogenesis described in the proposed model (Fig. 8).

Previous studies have demonstrated that RNA-binding proteins (RBPs) were frequently mutated in leukemia patients and essential for leukemia growth and differentiation [14–19], such as RBM17 and RBM39 in AML. Notably, identifying dysregulated RBPs in hematological malignancies has led to the rapid development of therapeutic strategies to target several RBPs specifically. For instance, R-2HG exhibits anti-tumor activity by targeting m6A writer FTO [48, 49], and sulfonamides were determined to degrade RBM39 selectively [18]. Although RBM5, RBM17, and RBM39 belong to the RNA-binding motif (RBM) family protein, their regulatory role in tumor progression is largely variable [50]. It is still a debate on how genes transcribed from the RBM5 locus functioned in cancers. Previous work reported that RBM5 is a tumor suppressor gene in lung cancer [26], and the gene locus antisense transcribed noncoding RNA, RBM5-AS1, is in contrast promoting tumorigenesis in breast and colon cancers [28, 29]. Our functional experiments in vitro and in vivo support the oncoprotein role of RBM5 in myeloid leukemia. Consistently, a recent study revealed that the modulation of RNA splicing enhanced response to BCL2 inhibition in AML, including the RBM10/RBM5 complex [51]. Although a previous study has indicated that the compound anthraquinone-2-sulfonic acid (AQ2S) inhibited the Ran-BP2-type ZF in the RBM5 [38], the activity of this small molecular or pharmacological approaches to inhibit RBM5 or in combination with other drugs, such as venetoclax, needs to be verified in the AML context. While importantly, an ideal therapeutical target would be selectively required for the leukemia growth but not the normal hematopoiesis. Our functional experiments by suppressing RBM5 in human normal CD34^+^ HSPCs did not affect the myeloid differentiation and the formation of myeloid or mixed-lineage colonies, suggesting RBM5 is not required for human normal hematopoiesis. Consistently, other studies reported that the non-sense mutation on the RRM domain of RBM5 in mouse models exhibited defects in sperm differentiation but not hematopoiesis [52]. More recent studies revealed that RBM5 is critical in neuronal injury response and participates in mouse models of Huntington’s disease [53, 54]. However, it remains unknown whether disruption of the zinc-finger domains or loss of the entire protein causes hematopoietic defect by in vivo genetic or transplantation models, which requires further comprehensive evaluation. Nevertheless, our study suggests that RBM5, the newly identified protein in AML, could provide an alternative potential therapeutic target.

RBPs regulate cancer development through multiple posttranscriptional processes, including alternative splicing, nucleocytoplasmic transport, translation, and degradation [13]. Moreover, many noncanonical functions of RBPs have been revealed recently [55–57]. For instance, RBPs directly regulate transcription in leukemia cells such as RBMX [14]. RBM25 directly regulates enhancer and promoter looping via interacting with the known chromatin looping factor YY1 [57]. Nevertheless, it is conceivable additional RBM family members exhibit similar functions in transcription regulation. Here, we demonstrated that both the RNA- and DNA-binding domains are essential for leukemia cell growth, suggesting that RBM5 functions as both RNA splicing and transcription regulation. More importantly, although RBM5 controls thousands of splicing events in leukemia cells, the differential genes after RBM5 loss were not due to the splicing defects. It remains unknown the relationship between the mRNA target and DNA target of RBM5 at the genome-wide scale, as well as the underlying mechanism to regulate the two processes. These questions require further comprehensive studies based on more high-throughput techniques, including CLIP-seq and ChIP-seq [57, 58]. In addition, the exact function of each protein domain of RBM5 still needs further in-depth characterization, such as protein structure analysis, due to the technical limitation of truncated mutation [36]. Significantly, the differential genes rather than the splicing events associate genes directly involved in the AML pathogenesis, including the FLT3 and HOXA9 targets. HOXA9, an oncogenic driver in leukemia, is regulated in multiple levels, including transcriptional, posttranscriptional, and translational levels via numerous known regulators [9–11, 17]. The acute inducible degradation system and inducible overexpression system proved that RBM5 directly activated the transcription of HOXA9 in an acute manner. In addition, the ChIP-qPCR demonstrated that RBM5 could directly bind to the *HOXA9* locus. It is known that HOXA9 directly occupies the FLT3 enhancer region and activates its transcription [59]. This is consistent with our evidence that HOXA9 could restore the FLT3 expression in RBM5 null leukemia cells (Fig. 7d and e). Therefore, RBM5 may activate FLT3 through upregulating HOXA9. However, the remaining question in the study is whether RBM5 interacts with other key transcriptional factors for gene transcription in leukemia. Because overexpression of HOXA9 rescued the defects of leukemia growth after RBM5 loss, we speculate that the noncanonical transcriptional regulation of HOXA9 by RBM5 is one of the primary mechanisms in AML.

## Conclusions

Overall, we find that RBM5 is a new regulator in HOXA9 regulation and controls acute myeloid leukemia development. The newly verified RBM5-HOXA9 axis provides a new example of how RBPs control mammalian transcription regulation.

## Methods

### Cell culture

Leukemia lines MOLM13, OCIAML2, THP1, U937, MV411, HL60, SEM, TF1, and HEL were maintained in RPMI-1640 medium (CORNING, 10–040-CV) containing 10% fetal bovine serum (FBS) (HyClone, 03.N16001DC). HEK293T cells were cultured in DMEM (HyClone, SH300022.01) supplemented with 10% FBS and 4.5-mM L-glutamine. The OCIAML2 and SEM cell lines were originally purchased from DSMZ. MOLM13 was originally purchased from ACCEGEN. The THP1, U937, MV411, and HL60 cell lines were originally purchased from the American Type Culture Collection (ATCC). The TF1 and HEL cell lines were obtained from Cell Resource Center, Peking Union Medical College (PCRC). The HEK293T cells (no. SCSP-502) were obtained from the Cell Bank of the Chinese Academy (www.cellbank.org.cn). Before using experiments, cell line identity was verified by STR analysis, and cells were confirmed as mycoplasma negative.

### Primary AML patients, normal bone marrow, and peripheral blood samples

All primary AML patients, normal bone marrow, and peripheral blood samples were obtained from the Hematological Biobank, Jiangsu Biobank of Clinical Resources, and informed consent forms were approved by the Ethics Committee of Soochow University in accordance with the Declaration of Helsinki. Following Ficoll-Paque separation, mononuclear cells were stored in the vapor phase of liquid nitrogen in 10% DMSO, 30% fetal bovine serum (FBS), and 60% Dulbecco’s Modified Eagle’s Medium (DMEM). Primary samples were thawed in warmed 50% FBS, 50% X-VIVO-15 with 100 μg/ml DNAse (AML), or 100% FBS (CB) before using in vitro assays. Primary AML samples were grown in X-VIVO-15 with 20% BIT Serum Substitute (Stem Cell Technologies), 100 ng/mL human SCF (Peprotech, catalog #300–07), 100 ng/mL human FLT3 (Peprotech, catalog #300–19-10UG), 20 ng/mL human TPO (Peprotech, catalog #300–18-10UG), 20 ng/mL human IL6 (Stem Cell Technologies, catalog #78,050), and 10 ng/mL human IL3 (Stem Cell Technologies, 78,042). The CD34^+^ hematopoietic stem and progenitor cells (HSPCs) were mobilized from normal subjects by granulocyte colony-stimulating factor, collected by apheresis, and enriched by immunomagnetic bead selection using the EasySep™ Human CD34-Positive Selection Kit (Stem Cell Technologies, #18,056), in accordance with the manufacturer’s protocol. The CD34^+^ HSPCs were cultured in StemSpan SFEM II (Stem Cell Technologies, 09650) with 20 ng/mL human TPO, 100 ng/mL human SCF, 100 ng/mL human FLT3, 20 ng/mL human IL6, and penicilin-streptonmycin. All cells were incubated at 37 °C in a humidified atmosphere containing 5% CO2.

### CRISPR library construction and screening

Human CRISPR knockout library (H3) was deposited by Drs. Shirley Liu and Myles Brown (Addgene #133,914). The library targets 18,436 human genes with 185,634 guide RNAs, with an average of 6 sgRNAs per gene. The sgRNAs are selected based on computationally predicted and experimentally observed maximum KO efficiency. The HOXA9^P2A−mCherry^ reporter cell line (OCIAML2 and SEM) was infected with lentiviral Cas9 followed by re-infection of pooled sgRNA library at low M.O.I (~ 0.3). Infected cells were selected by blasticidin and puromycin and later sorted for mCherry^High^ and mCherry^Low^ populations between days 10–12. The sgRNA sequences were recovered by genomic PCR analysis and deep sequencing using NovaSeq 6000 for single-end 150-bp read length (Illumina). The differentially represented sgRNAs were calculated by DEseq2 analysis [60] and combined for MAGeCK analysis at the gene level [61].

### RBM5 domain targeting CRISPR library construction and screening

A total of 186 20-bp sgRNAs were designed by FlashFry (version 1.12) to span all RBM5 exons. One-hundred and twenty positive control sgRNAs and 100 negative control sgRNAs were also included. The oligonucleotide pool of the sgRNA library was synthesized by CustomArray. Forward library PCR primer (5′-GGCTTTATATATCTTGTGGAAAGGACGAAACACC-3′) (10 μM) and reverse library PCR primer (5′-CTAGCCTTATTTTAACTTGCTATTTCTAGCTCTAAAAC-3′) (10 μM) were used to amplify the sgRNA oligonucleotides using 2X HiFi CloneAmp PCR mixture (Clontech) under the following PCR conditions: 98 °C 3 min, 98 °C 10 s, 55 °C 10 s, 72 °C 10 s, 72 °C 5 min, 4 °C hold for 12 cycles. The amplified product was run on a SybrGreen stained 2% agarose gel, and bands were excised for gel purification by a Qiagen Gel Purification kit. The amplified sgRNAs (10 ng) were cloned into the LentiGuide-Puro (Addgene, #52,963) backbone cut by BsmB1 (100 ng) using the NEbuilder HiFi DNA assembly master mix (NEB) at 50 °C for 1 h. Eight 50-μL vials of NEB Stable Competent *E. coli* High-Efficiency cells (NEB, C3030H) were thawed on ice, and 2 μL of the assembled reaction was added to each. Cells were incubated on ice for 30 min, heat shocked at 42 °C for 30 s, and then placed on ice. NEB 10-beta/stable outgrowth medium was added to the heat-shocked cells (950 mL per vial) and incubated at 30 °C for 60 min at 250 rpm. Recovered cells were plated at 2.5 mL per square LB + ampicillin dish (245 mm × 245 mm) for four plates and incubated at 30 °C overnight. Bacterial colonies were counted to ensure good library coverage and collected for DNA extraction of the pooled sgRNA library by Qiagen Maxi prep (Qiagen). The OCIAML2 cells stably expressing lentiviral Cas9-blasticidin were infected with the pooled sgRNA library at low MOI (~ 0.3). Infected cells were selected with blasticidin and puromycin for 3 days. On day 7 post-antibiotic selection, cells were collected. The sgRNA sequences were recovered by genomic PCR analysis and indexed (Nextera, FC-131–1096) and sequenced using NovaSeq 6000 for single-end 151 bp read length (Illumina).

### Data analysis of CRISPR screening

The raw FASTQ data obtained after HiSeq sequencing were demultiplexed and mapped to the original reference sgRNA library for data analysis. The read counts for each sgRNA were normalized against the total read counts across all samples. The differentially enriched sgRNAs were defined by comparing normalized counts between sorted cells in the top 10% and those in the bottom 10% of mCherry-expressing bulk populations. Two independent screenings were performed with the HOXA9^P2A−mCherry^ reporter cell line stably expressing Cas9. The sgRNA rank was displayed based on a *P*-value and log_2_ fold change by DESeq2 [60]. The gene ranking analysis for significant genes and related GO (gene ontology) analysis were conducted using the MAGeCK, MAGeCKFlute, and EnrichR [61–63].

### CRISPR-Cas9-mediated gene targeting and vector construction

The Cas9-expression vector, Lenti-Cas9-Blast, was purchased from Addgene (#52,962). Cas9 protein was introduced to human AML cell lines by lentiviral transduction and selected with 10 μg/mL blasticidin (Gibco, A11139-03) to generate Cas9-stable cell lines. The sgRNA sequences were selected from the CRISPR library or using a CRISPR design tool (http://www.crisprscan.org/) and generated as oligonucleotide pairs. After annealing, constructs were cloned into the Lenti-guide-puro-IRES-GFP vector (a sgRNA-expression vector), which encodes the sgRNA. Cells were transduced with sgRNA lentivirus and puromycin (Gibco, A11138-03) selection for 72 h after transduction. The oligonucleotides encoding sgRNAs are listed in Additional file 8: Table S7. For the rescue experiments, the cDNAs of HOXA9, FLT3, and RBM5 were cloned into the Lenti-EFS-P2A-mVenus (GFP) vector using the Gibson Assembly. The CRISPR-resistant synonymous mutant of RBM5 and domain mutant of RBM5 were cloned using PCR mutagenesis. For the exogenous RBM5-mini-AID (auxin-induced degron) system, RBM5-N-terminal-mini-AID was PCR amplified from the in-house-made Lenti-EFS-RBM5-P2A-mVenus (PAM-MUT) vector and inserted into lentiviral expression vector. For the RBM5 inducible overexpression system, the cDNA of RBM5 was cloned into the Lenti-TRE3G-HA^tag^-MND-Zeo backbone by Gibson Assembly. SnapGene software was used to design all primers used for cloning. The PCR amplifications of products for cloning were performed using PrimeSTAR Max Premix (TaKaRa, R045). A Gibson Assembly Cloning Kit (Abclonal, RK21020) was used in accordance with the manufacturer’s instructions.

### Generation of a RBM5-HA-miniAID-P2A-GFP endogenous knock-in leukemia lines

For RBM5-HA-miniAID-P2A-GFP knock-in delivery, 100 ng of the donor plasmid, and 100 ng of sgRNA/Cas9 ribonucleoprotein (RNP) were used for 500,000 SEM and MOLM13 cells. SEM cells were electroporated using the Nucleofector-4D device (Lonza) with the Lonza Cell Line Nucleofector SF solution and program EH100. MOLM13 cells were electroporated using the Nucleofector-4D device (Lonza) with the Lonza Cell Line Nucleofector SF solution and program CM138. Twenty-four hours after electroporation, cells were sorted for the GFP fluorescent marker to enrich the knock-in cell population. After the sorted cells recovered in culture for up to 3 weeks, a second sort was performed to select cells for successful knock-in by sorting for cells expressing the knock-in GFP fluorescent marker. Two weeks later, a third sort was repeated to identify single-cell-derived clones based on the high GFP expression.

### shRNA-mediated gene knockdown

Oligonucleotides encoding short hairpin RNA (shRNA) constructs were designed by the RNAi Consortium of the Broad Institute, obtained from IDT, and cloned into the lentiviral vector pLKO.1-PURO, and luciferase shRNA-encoding pLKO.1-NT (shNT, non-targeting) was used as a control. Cells were transduced with shRNA lentivirus and then selected with 2 μg/ml puromycin for 72 h. The oligonucleotides encoding shRNAs are listed in Additional file 8: Table S7.

### Virus production and transduction

Lentivirus was produced in HEK293T cells by transfecting lentiviral plasmids with helper packaging plasmids (VSVG and psPAX2) using the polyethylenimine (PEI 40000; MAOKANGBIO, 49,553–93-7) transfection reagent. HEK293T cells were plated in 10-cm culture dishes and were transfected when confluency reached ~ 80–90%. For one 10-cm dish of HEK293T cells, 12 μg of plasmid DNA, 4 μg of pVSVG and 8-μg psPAX2, and 96 μL of 1 mg/mL PEI were mixed, incubated at room temperature for 20 min, and then added to the cells. The fresh medium was changed 6–8 h post-transfection. Lentivirus soup was collected at 48- and 72-h post-transfection. The collected virus was filtered through a 0.45-μM non-pyrogenic filter.

### *Primary human AML cells and CD34*^+^*HSPCs transduction*

For primary AML samples, 0.5 million cells were infected with lentivirus using an MOI of 50 in 24-well ultralow attachment plates with 500-µl total growth media, and another 500 µl of growth media was added 16 h after infection, followed by puromycin selection for 24 h. For CD34^+^ HSPCs transduction, lentivirus was added at an MOI of 50, and cells were selected using puromycin selection for 24 h. Cells were grown for another 2 days before further study.

### *RBM5 editing in normal donors derived CD34*^+^*HSPCs*

CD34^+^ HSPCs were electroporated using the Neon™ NxT Electroporation (Neon) with the Neon™ Transfection Kit (Invitrogen by Thermo Fisher Scientific, MPK1096B). The electroporation program is 1600 V, 10 ms, and 3 pulse. For ribonucleoprotein (RNP) complex delivery, 50 µM of RBM5 sgRNA#1 (synthesized by GenScript) and 5 µg of Cas9 protein (Integrated DNA Technologies, 1,081,058) (molar ratio of sgRNA:Cas9 is 1:3) were used for 0.2 million CD34^+^ HSPCs. Nontarget sgRNA (NT) was used as a negative control. After 24 h of electroporation, cells were washed using PBS and transferred to a fresh growth medium. After 48 h of electroporation, the cells will be used for functional experiments and knockout efficiency assessment.

### Myeloid differentiation

To differentiate HSPCs into myeloid lineage cells, a total of 1 × 10^4^ CD34^+^ cells (in a volume of 1 mL) were seeded into each well of a 12-well plate (1 mL/well) and cultured in SFEM II supplemented with StemSpan Myeloid Expansion Supplement (STEMCELL Technologies, 02693). Every 2 days, 500 μL of fresh medium was added. Cells were collected by centrifugation (350 × g, 5 min) and resuspended in fresh medium every 7 days. Cells were analyzed by flow cytometry on days 7 and 14 for expression of myeloid lineage markers, using FITC-conjugated anti-CD14 (BioLegend, 325,603) and APC-conjugated anti-CD11b (BioLegend, 301,310) antibodies.

### Colony-forming unit assay

The clonogenic potential was assessed by seeding the indicated number of cells in methylcellulose media. For RBM5-knockdown leukemic cell lines, to make 100-mL complete methylcellulose media, 40-mL methylcellulose base media (Stem Cell Technologies, MethoCult H4230) was supplemented with IMDM (HyClone, SH30228.01), 10% FBS, 1% penicillin–streptomycin, and other desired supplements as described previously in the cell culture section. The complete media was then used to resuspend the cells. Sixteen hours (for primary AML) or 24 h or 72 h (for CD34^+^ HSPCs) post lentiviral infection, freshly selected using puromycin for 24 h (healthy shRNA-expressing), cells were plated in complete methylcellulose media (STEMCELL Technologies, MethoCult H4230) (primary AML) or Human Methylcellulose Complete Media (STEMCELL Technologies, 04034) (CD34^+^ HSPCs) and grown in 37 °C, 5% CO_2_ incubator for 10–14 days before imaging and counting. For primary AML, ~ 7000–30,000 cells per milliliter were plated. For CD34^+^ HSPCs, 750–1000 cells per/ml were plated.

### Cell proliferation experiments

About 1 × 10^5^ cells per well (in a total volume of 2 mL) were seeded in each well of a 6-well plate and cultured in RPMI-1640 medium containing 10% fetal bovine serum (FBS). Every 3 days, 1 mL of fresh medium was added. Cell number was counted on day 3 and day 5, respectively, and the proliferation curve was drafted by Prism software.

### Competition-based cell proliferation assay

Cas9-expressing cell lines were transduced with lentiviral pXPR-guide-puro-IRES-GFP linked with a GFP reporter. The percentage of the GFP-positive cell population was measured at day 3 as the initial time point. The GFP% was then measured every 3 days over a time course. The relative change in the GFP% percentage at each time point was then normalized to the initial time point GFP%. This relative change was used to assess the impact of individual sgRNAs on cellular proliferation, which reflects cells with a genetic knockout being outcompeted by non-transduced cells in the cell culture. Flow cytometry was performed on BD LSRFortessa (BD Biosciences) and analyzed with FlowJo software (Tree Star).

### Flow cytometry and cell sorting

For flow cytometric analysis of myeloid differentiation markers CD11b and CD14, cells were washed with PBS and then stained with FITC-CD11b antibody (BioLegend, 325,603) or APC-CD14 antibody (Biolegend, 301,310) at 4 °C temperature for 20 min and then washed twice with cold PBS for flow cytometric analysis. For Annexin-V cell apoptosis (Beyotime, C1062M) analysis, cells were stained following standard protocol. Flow cytometry was performed on BD LSRFortessa (BD Biosciences) and analyzed with FlowJo software (Tree Star). Fluorescence-activated cell sorting (FACS) of AML cells was performed by BD FACSAria (BD Biosciences) according to the manufacturer’s instructions.

### Animal xenograft studies

NOD/SCID/gamma (NSG) mice used in this study were 6-week females purchased from GemPharmatech and maintained in the mouse facility at the School of Medicine at the University of Soochow. All mouse procedure protocols utilized in this study were in accordance with protocols approved by the Institutional Animal Care and Use Committee (IACUC) of Soochow University. For experiments validating the in vivo requirement of RBM5 in leukemia cells, MOLM13-luciferase-GFP cells were lentivirally transduced with pLKO.1.5-PURO vectors targeting RBM5 (shRBM5#1 and shRBM5#2) or negative control (shNT). Cells were transduced with shRNA lentivirus and then selected with 2 μg/ml puromycin for 72 h before a proliferation suppression phenotype manifests, GFP^+^ (shRNA infected) populations were collected, and 0.5 million cells were injected through the tail vein of NSG mice. On day 18 post-transplantation, 4 mice of the shNT group were moribund, and 2 mice of the shRBM5-KD group were sacrificed. To quantify leukemia burden, bone marrow, spleen, and peripheral blood were flushed and collected. Red blood cells were lysed with lysis buffer (Beyotime) on ice for 5 min, and the remaining cells were stained with human CD45 (1:50). The percentage of GFP^+^ and CD45^+^positive cells were analyzed on BD LSRFortessa (BD Biosciences). GFP^+^ cells collected from bone marrow were sorted with a BD FACSAria for Western blotting. Kaplan–Meier survival curves and log-rank tests were performed using Prism software (GraphPad).

### Quantitative PCR (qPCR) analysis for gene expression

Total RNA was extracted from AML cells using the FastPure Cell/Tissue Total RNA Isolation Kit (Vazyme, RC112-01). cDNA was generated using PrimeScript™ RT Master Mix (Takara, RR036Q) from 1-μg RNA and diluted 1:200 for qPCR analysis. qPCR was performed using 1-μL diluted cDNA with biological and technical replicates using SYBR Green Master Mix (Vazyme, Q511-03) with QuantStudio 6 real-time PCR system, and results were normalized to the expression of ACTB. Primer sequences utilized for qPCR are in Additional file 8: Table S7.

### Immunoblotting

Cells were washed with PBS and lysed in Cell Lysis Buffer for Western and IP (Beyotime, #P0013) with a protease inhibitor cocktail. Lysates were heated to 95° in SDS sample buffer, separated by SDS-PAGE, and transferred to a nitrocellulose membrane. Membranes were blocked in 5% nonfat milk in PBS with 0.1% Tween-20, probed with indicated primary antibodies, and followed by incubation with HRP-coupled secondary antibody for 1 h at room temperature. Blots were visualized using enhanced chemiluminescence detection reagents and exposed to X-ray film. Representative plots of three biological replicates were shown. All antibodies used in this study are listed in Additional file 8: Table S7.

### RNA-seq analysis

RNA samples of three biological replicates were extracted from cultured cells, using TRIzol (Ambion by Life technologies, 343,911), following the manufacturer’s instructions. RNA was then sent out for library preparation and next-generation sequencing to a commercial company, Novogene (CA, USA). Raw counts of gene transcripts were derived from raw fastq files using the alignment-independent quantification tool, Salmon (https://combine- lab.github.io/salmon/) with standard settings. The raw count matrix was then imported into RStudio and utilized as input for DESeq2 analysis following the vignette of the package for normalization, differential gene expression analysis, and unbiased clustering analysis, including principal component analysis. The output of DESeq2 was used as the input for pre-ranked-based GSEA to enrich functional pathways and gene signatures (https://www.gsea-msigdb.org/gsea/index.jsp).

### ChIP-qPCR

Five million cells were used for the ChIP assay of each target. Cells were washed with PBS, crosslinked with 1% formaldehyde for 5 min at room temperature, and then quenched with 125-mM glycine for 5 min. Wash cells twice with ice-cold PBS. The isolated nuclei were resuspended in 250-µl nuclei lysis/sonication buffer and sonicated with XINCHEN VXV130 sonicator with the following parameters: duty cycle 22% and time: 20 s on and 20 s off for 9–12 cycles. After centrifugation at 4° with 13,500 rpm for 10 min, soluble chromatin was used to perform immunoprecipitation, while 5% of the sample was kept as input DNA. Immunoprecipitation was performed with 5 ~ 10 μg indicated Pierce Anti-MYC Magnetic Beads (Thermo Scientific, 88,842), H3K4me3 (PTM BIO, PTM613), or IgG (Abclonal, AC011) overnight at 4° with rotation and then washed sequentially using low salt, high salt, LiCl buffer, and TE buffer. Bound DNA was then eluted, reverse-crosslinked, and incubated with RNase A and proteinase K. DNA samples were purified using a Universal DNA Purification Kit (Tiangen, DP214-02). Primers for ChIP-qPCR analysis were listed in Additional file 8: Table S7.

### Differential splicing analysis

rMATS v4.0.1 (turbo)76 software was used to perform differential splicing analysis between MOLM13 control (CTRL), RBM5 knockout (RBM5 KO), and RBM5 overexpression (RBM5 OE) cells 3 days after transduction. The human hg19 annotation was adopted as the background reference (GENCODE GRCh37.v19.annotation.gtf). Alternative splicing events of five kinds (CE, A5SS, A3SS, MXE, and RI) were determined by an *FDR* < 0.05 as well as inclusion level differences larger than 0.1 between control and RBM5 knockout cells.

### Gene Expression Profiling Interactive Analysis (GEPIA)

Expression of RBM5 across different types of cancer and normal tissues was analyzed using an online tool, Gene Expression Profiling Interactive Analysis (GEPIA) (http://gepia.cancer-pku.cn/).

### Cancer dependency map portal data analysis

The DepMap portal (https://depmap.org/portal/), the CRISPR (Avana) Public 20Q2 dataset, and the Combined RNAi (Broad, Novartis, Marcotte) dataset were used for the analysis. No samples were excluded from the dataset in this analysis. Following the DepMap instruction, the dependency scores of annotated genes were downloaded and then plotted as dot plots. Pearson correlation was then calculated for all the plots using Prism.

### Software and statistical analysis

Prism software and R were used for data processing, statistical analysis, and result visualization (http://www.graphpad.com). The R language and environment for graphics (https://www.r-project.org) were used in this study for the bioinformatics analysis of the CRISPR screen and RNA-seq data. The R packages used for all analyses described in this manuscript were from the bioconductor and CRAN. On graphs, bars represent the standard error of the mean (SEM), as legends indicate. *P* < 0.05 was considered statistically significant for all figures, *Indicates *P* < 0.05, **P < 0.01, and ***P < 0.001.

### Supplementary Information

**Additional file 1. Figure 1S.** Genome-wide CRISPR/Cas9 screening identifies RNA splicing factor RBM5 as a novel regulator for HOXA9 expression in acute leukemia. **Figure 2S.** Disruption of RBM5 delays the growth of leukemia cells in vitro. **Figure 3S.** RBM5 knockdown impairs in vivo myeloid leukemia engraftment. **Figure 4S.** RBM5 suppression does not affect human normal hematopoiesis. **Figure 5S.** Protein structure prediction of RBM5. **Figure 6S.** Identification of RBM5 downstream target genes in AML. **Figure 7S.** HOXA9 is a functional target gene of RBM5 in AML.

**Additional file 2: Table S1.** Positive regulators of HOXA9 from genome-wide CRISPR/Cas9 screen in OCIAML2 and SEM cells.

**Additional file 3: Table S2.** The differentially expressed genes (DEGs) in RBM5 high group vs. RBM5 low groups from public RNA-seq dataset for 44 AML patients.

**Additional file 4: Table S3.** RBM5 domain CRISPR/Cas9 screen after 7 days expansion in OCIAML2 cells.

**Additional file 5: Table S4.** The DEGs in RBM5 KO relative to control and RBM5 OE relative to control.

**Additional file 6: Table S5.** The associated genes with differential splicing events in RBM5KO and RBM5 OE relative to control.

**Additional file 7: Table S6.** The DEGs in in RBM5 KO cells with HOXA9 overexpression relative to RBM5 KO control.

**Additional file 8: Table S7.** Oligonucleotides, knock-in fragment, primers, and antibodies used in this study.

**Additional file 9.** Uncropped images for the blots in Fig. 2, 3 and 4, Fig. 6 and 7 and supplementary Fig. 2, 3, 4, 7.

**Additional file 10.** Review history.

## Data Availability

RNA-seq data generated in this study are deposited in GEO under the accession number GSE225633 [64]. Publicly available datasets (Kramer et al., Proteomic and Phosphoproteomic Landscapes of Acute Myeloid Leukemia, Blood, 2022) were referenced [33]. Code repositories collected at 10.6084/m9.figshare.c.6186670 included all RNA-seq data collected from this study [65].

## Declarations

### Peer review information

Wenjing She was the primary editor of this article and managed its editorial process and peer review in collaboration with the rest of the editorial team.
