# Supplementary material for: RNA-binding protein RBM5 plays an essential role in acute myeloid leukemia by activating the oncogenic protein HOXA9
Source: Genome Biol. 2024 Jan 12;25:16. doi: 10.1186/s13059-023-03149-8 (PMC10785552; doi:10.1186/s13059-023-03149-8)
Supplement: Supplementary file 1 — Additional file 1. Figure 1S. Genome-wide CRISPR/Cas9 screening identifies RNA splicing factor RBM5 as a novel regulator for HOXA9 expression in acute leukemia. Figure 2S. Disruption of RBM5 delays the growth of leukemia cells in vitro. Figure 3S. RBM5 knockdown impairs in vivo myeloid leukemia engraftment. Figure 4S. RBM5 suppression does not affect human normal hematopoiesis. Figure 5S. Protein structure prediction of RBM5. Figure 6S. Identification of RBM5 downstream target genes in AML. Figure 7S. HOXA9 is a functional target gene of RBM5 in AML. [file 13059_2023_3149_MOESM1_ESM.docx]

**Supplementary Figures and figure legends**

**
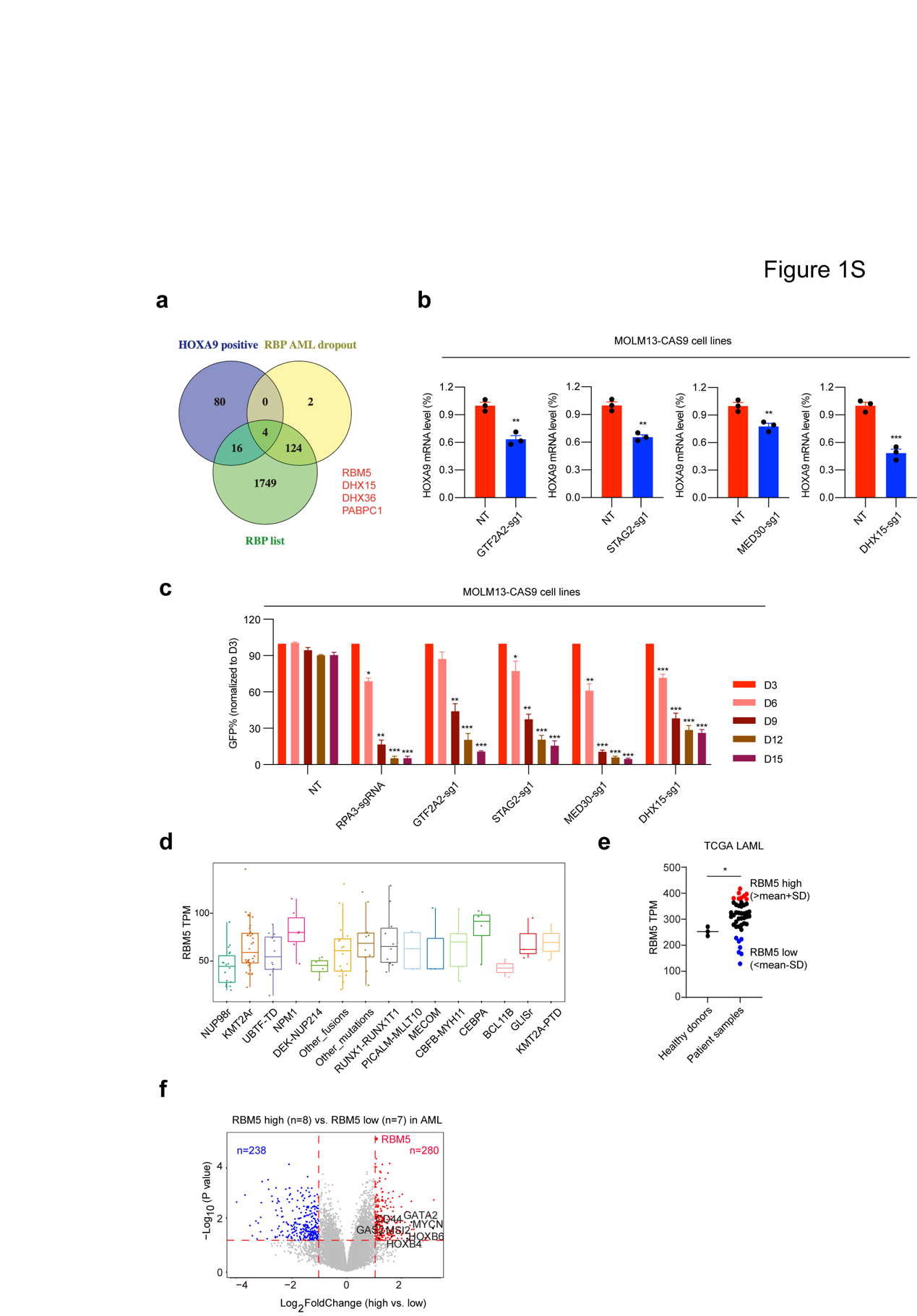
**

**Figure 1S. Genome-wide CRISPR/Cas9 screening identifies RNA splicing factor RBM5 as a novel regulator for HOXA9 expression in acute leukemia**

**a.** Venn diagram showing the overlapped genes including RBM5, DHX15, DHX36 and PABPC1 among RBPs (RNA binding proteins) gene lists, RBP AML dropout screen candidates from Depmap, and RBPs as HOXA9 potential positive regulators in whole genome library screening results. **b.** Real-time-qPCR analysis was conducted on the sgGTF2A2, sgSTAG2, sgMED30 or sgDHX15 targeted MOLM13-Cas9 cell to monitor the mRNA of HOXA9. Non-target (NT) was used as a negative control. Data shown are means ± SEM from three independent experiments. **P < 0.01, *** P < 0.001, unpaired Student’s t-test. **c**. Competitive proliferation assay was conducted in Cas9 stably expressed MOLM13 cells after transduced with GFP reporter-based lentiviral sgRNAs (NT, RPA3, GTF2A2-sg1, STAG2-sg1, MED30-sg1 and DHX15-sg1) at about ~50% efficiency. The GFP% was quantified on days 3, 6, 9, 12, and 15 by flow cytometry to evaluate the growth effect. The guide RNA targeting the survival essential gene RPA3 was included as a positive control, and the guide RNA targeting the non-target (NT) gene was included as a negative control. Data shown are means ± SEM from three independent experiments. * P < 0.05, ** P < 0.01, *** P < 0.001, unpaired Student’s t-test. **d.** Box plots show the mRNA expression of RBM5 in different AML subtypes. The original data is from the St. Jude Cloud. TPM: transcripts per million. **e.** The RBM5 mRNA levels in 44 AML patient samples and three healthy donors from TCGA. Two groups were classified as RBM5 high (n=8) and RBM5 low (n=7) based on the cutoff TPM > mean + SD and TPM < mean - SD, respectively. **f**. Volcano plot showing the differentially expressed genes between the RBM5 high group versus the RBM5 low group. Genes with P < 0.05 and |Log[fold change(high/low)]| > 1 were highlighted.

**
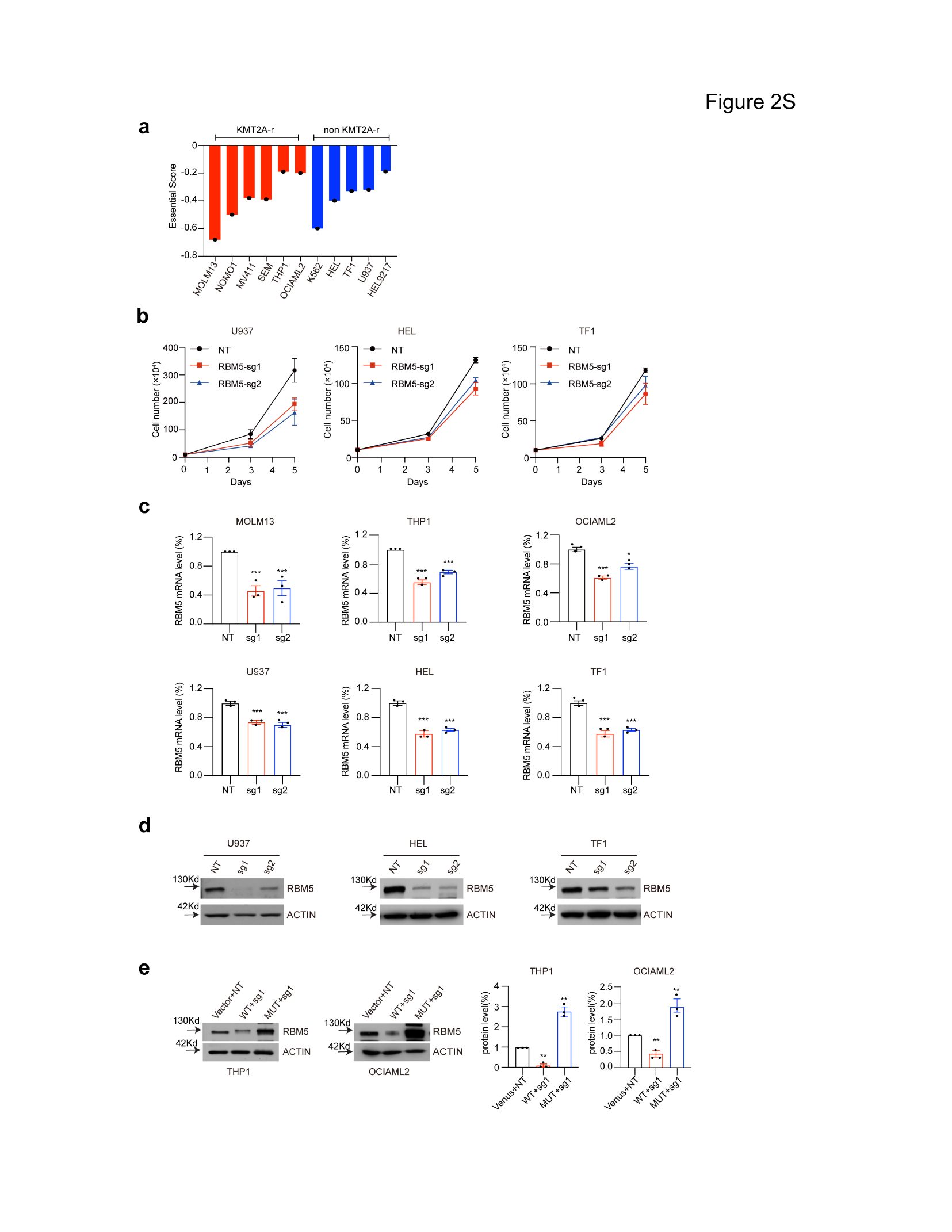
**

**Figure 2S. Disruption of RBM5 delays the growth of leukemia cells *in vitro***

**a**. Gene essential score of RBM5 in both KMT2A-r and non-KMT2A-r cell lines from Depmap. **b**. Cell counting assay was conducted on the RBM5-sg1, RBM5-sg2, and sgNT targeted U937-Cas9, HEL-Cas9 and TF1-Cas9 to monitor the ability of proliferation of AML cells. Guide RNA targeting non-target gene (NT) was included as a negative control. **c.** Real-time-qPCR analysis was conducted on the RBM5-sg1, RBM5-sg2, and NT-targeted leukemia cell lines to monitor the reduction of RBM5. Data shown are means ± SEM from three independent experiments. **P < 0.01, *** P < 0.001, unpaired Student’s t-test. **d.** Immunoblotting of RBM5 in RBM5 sgRNAs targeted cells. And the bands were scanned and statistically analyzed. Data shown are means ± SEM from three independent experiments. * P < 0.05, ** P < 0.01, *** P < 0.001, **** P < 0.0001, unpaired Student’s t-test. **e.** Immunoblotting was conducted by infecting THP1 and OCIAML2 cells overexpressing ectopic empty vector, RBM5-wild type cDNA (WT), RBM5-sgRNA1 resistant mutant cDNA (PAM-MUT), with lentiviral- sgRNAs against non-target gene (NT) and RBM5 (RBM5-sg1), and the bands were scanned and statistically analyzed. Data shown are means ± SEM from three independent experiments. ** P < 0.01, unpaired Student’s t-test.


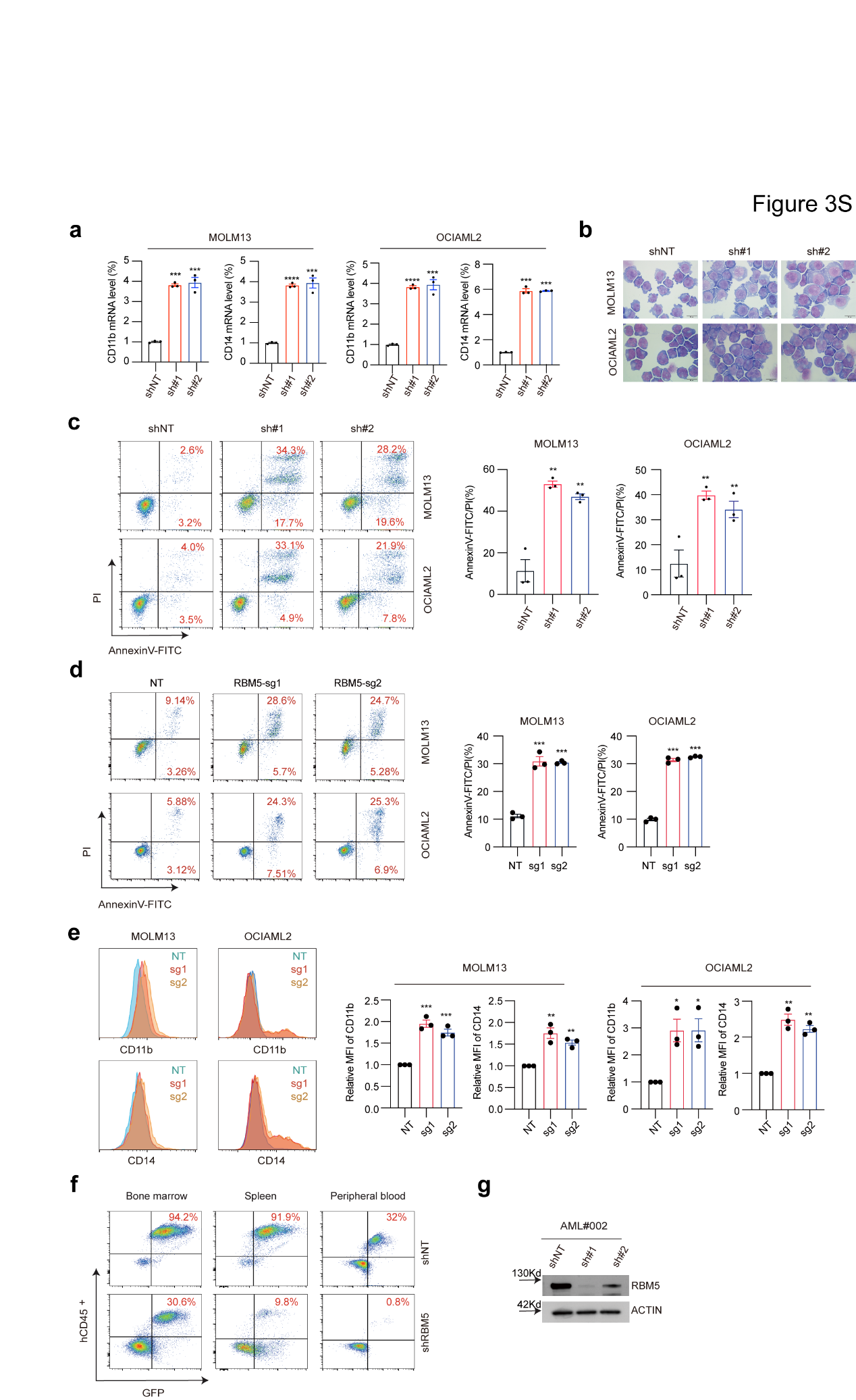


**Figure 3S. RBM5 knockdown impairs *in vivo* myeloid leukemia engraftment**

**a.** mRNA expression of CD11b and CD14 after six days of shNT, shRBM5#1(sh#1), and shRBM5#2(sh#2) viral transduction in MOLM13 and OCIAML2 cells. Data shown are means ± SEM from three independent experiments. *** P < 0.001, unpaired Student’s t-test. **b.** Gimesa staining was performed on cell morphology after lentiviral transduction of shRNA against control shNT and shRNAs targeting RBM5 (sh#1 and sh#2) in MOLM13 and OCIAML2 cells. **c.** Representative flow cytometry analysis of PI (indicating permeable dead cells) and Annexin-V staining (a pre-apoptotic cell marker) following lentiviral transduction of MOLM13 and OCIAML2 cells (day 6). Quantification of apoptotic cells, an average of 3 biological replicates. ** P < 0.01, unpaired Student’s t-test was applied to calculate the P value. **d**. Representative flow cytometry analysis was conducted to assess propidium iodide (PI), and Annexin-V staining, following lentiviral transduction of MOLM13 and OCIAML2 cells on day 6. Quantification of apoptotic cells, an average of 3 biological replicates. ** P < 0.01, unpaired Student’s t-test was applied to calculate the P value. **e**. Surface expression of CD11b and CD14 after lentiviral transduction of sgRNA against control NT and sgRNAs targeting RBM5 (sg1 and sg2) in MOLM13 and OCIAML2 cells. MFI, mean fluorescence intensity. * P < 0.05, ** P < 0.01, unpaired Student’s t-test. **f**. Flow cytometry analysis of the percentage of human CD45^+^ and GFP^+^ leukemia cells in bone marrow, spleen, and peripheral blood of recipient mice in the shNT (n=4) and shRBM5 (n=2) groups sacrificed after 18 days post-transplantation. **g**. Immunoblotting of RBM5 in RBM5 shRNAs (sh#1 and sh#2) targeted primary AML#002 sample.


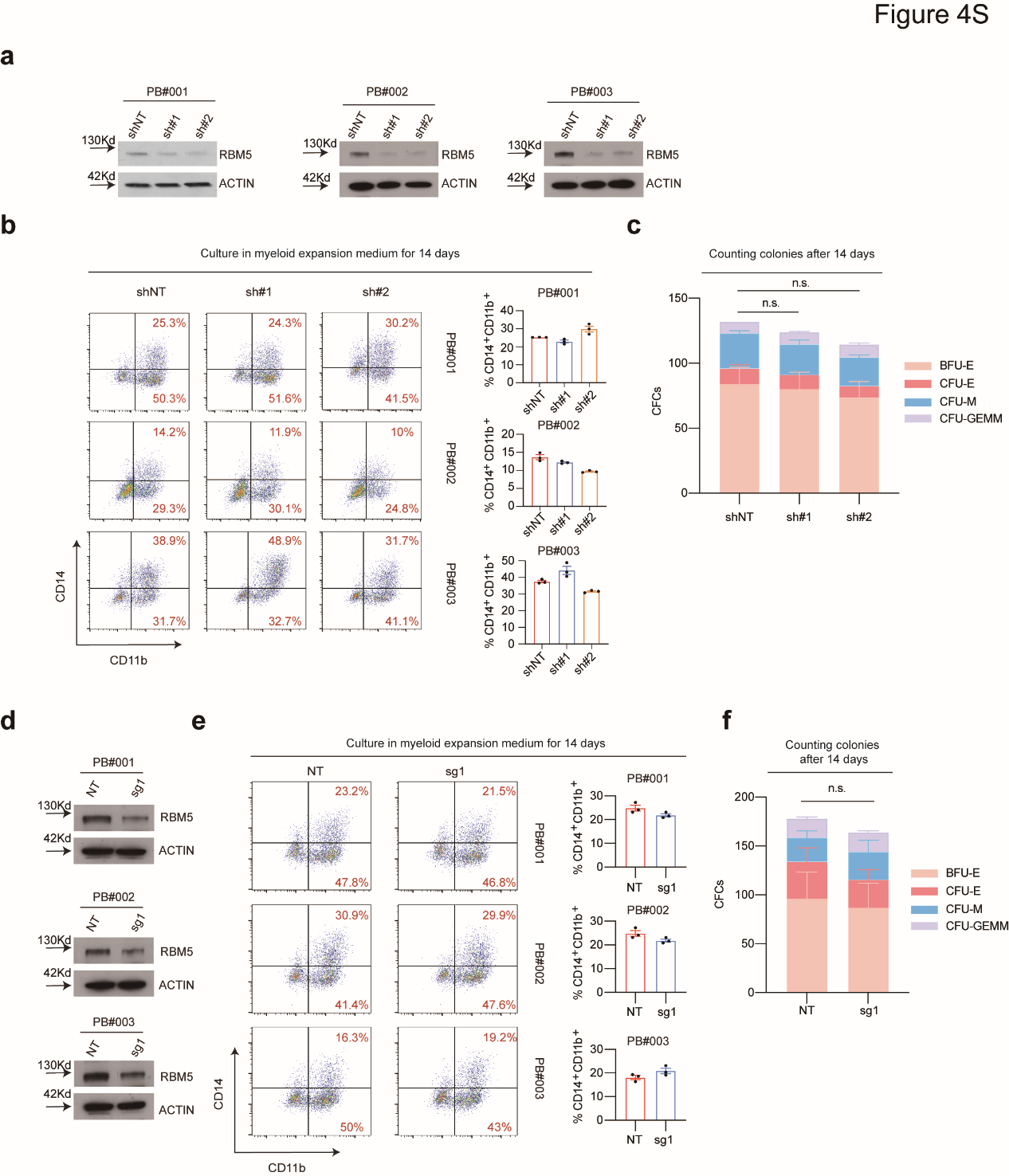
**Figure 4S. RBM5 suppression does not affect human normal hematopoiesis**

**a**. Immunoblotting of RBM5 in normal PB (#001-#003) HSPCs with and without RBM5 knockdown. **b**. Flow cytometry analysis of the percentage of myeloid markers CD11b and CD14 in day 14 myeloid expansion cultures. The bar graph displays the percentage of double-positive cells for CD11b and CD14, with three parallel replicates from three normal donors. **c**. Count of colonies in CFU assay in day 14 cultures from transduced CD34^+^ HSPCs with and without RBM5 knockdown, with three parallel replicates from three normal donors. **d**. Immunoblotting was performed to analyze RBM5 expression in normal PB (#001-003) HSPCs both with and without RBM5 knockout. **e**. Flow cytometry analysis was conducted to evaluate the percentage of myeloid markers CD11b and CD14 in day 14 myeloid expansion cultures. The accompanying bar graph illustrates the percentage of CD11b and CD14 double-positive cells, with data derived from three parallel replicates from three normal donors. **f**. Counting colonies in the CFU assay was carried out on day 14 cultures derived from transduced CD34^+^ HSPCs, comparing those with and without RBM5 knockout. The experiment included three parallel replicates sourced from three different normal donors.

**Figure 5S. Protein structure prediction of RBM5**

1. Schematic of the 3D structure of the RBM5 protein from the AlphaGo prediction.


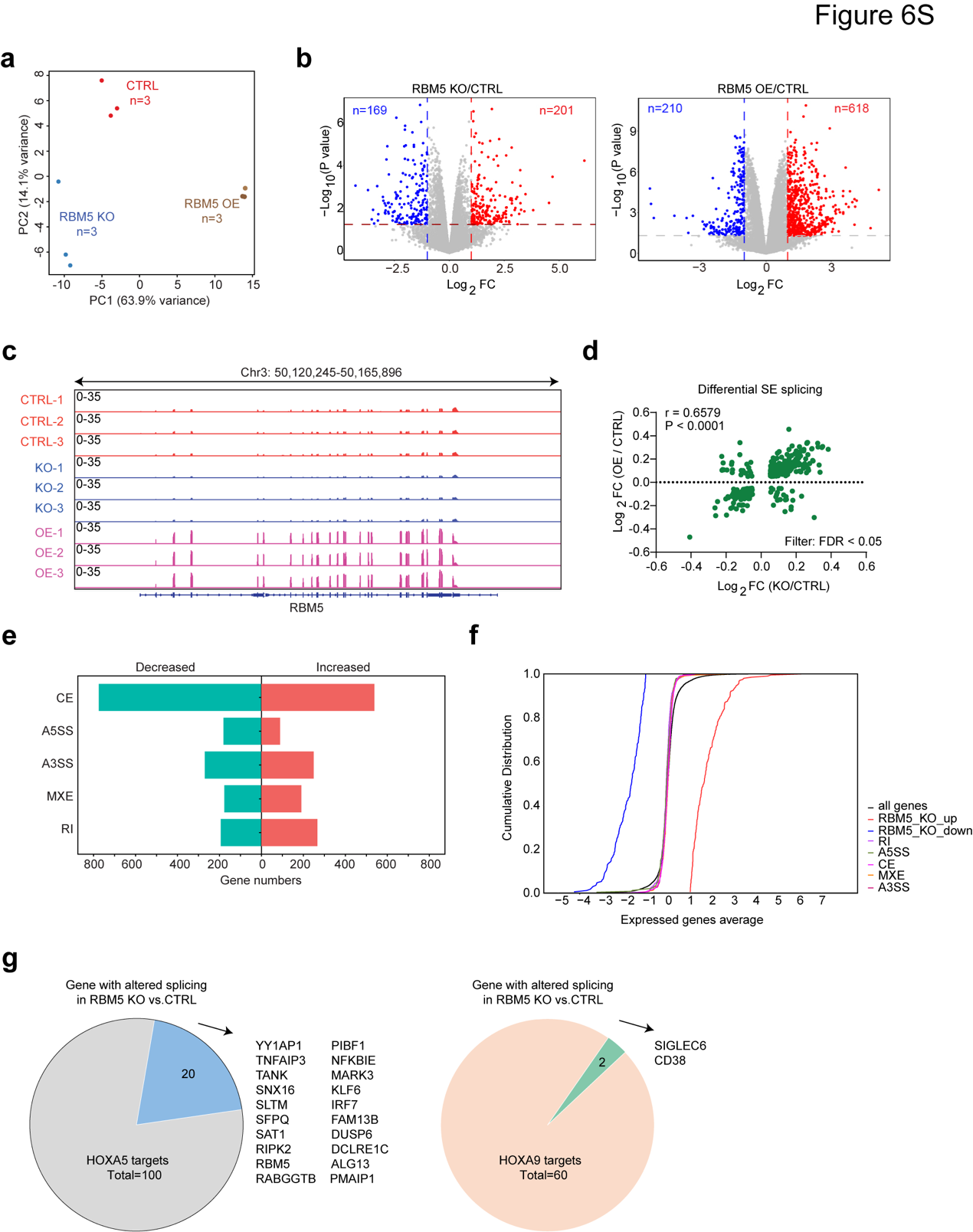


**Figure 6S. Identification of RBM5 downstream target genes in AML**

**a.** PCA score plot for the transcriptome analysis in three groups of MOLM13 cells, including the CTRL group (Control cells with NT sgRNA treated), RBM5 KO group (RBM5 knockout cells with RBM5-sg1 treated), and RBM5 OE group (RBM5 overexpressed cells by lentiviral RBM5-P2A-Venus vector). Each group was performed with three biological replicates. **b.** Volcano plot at left showing the differentially expressed genes between RBM5 KO versus CTRL (control) in MOLM13 cells eight days post viral transduction. The graph at right shows differentially expressed genes between RBM5 OE versus CTRL. Genes with P < 0.05 and |Log2fold change| > 1 were highlighted (blue: genes significantly decreased, red: genes increased considerably). **c.** Genome tracks showing the change of RBM5 expression in CTRL, RBM5 KO, and RBM5 OE groups. **d.** Integrated scatter plot analysis comparing different splicing events (SE) following RBM5 OE/CTRL and RBM5 KO/CTRL in MOLM13 cells. False discovery rate [FDR] < 0.05. **e.** Number of differentially spliced genes in MOLM13 cells treated with RBM5 KO versus control. Cassette exon (CE). A5SS: Alternative 5’ splice sites. A3SS: Alternative 3’ splice sites. MXE: Mutually exclusive exons. RI: Retained intron. **f.** Cumulative distribution function (CDF) of the averaged RBM5 KO to CTRL ratio for differentially expressed genes and five types of differential splicing events associated genes. **g.** Pie charts showing the distribution of the HOXA9-related downstream target genes and their undergoing altered splicing events after RBM5 knockout (from RNA-seq dataset).


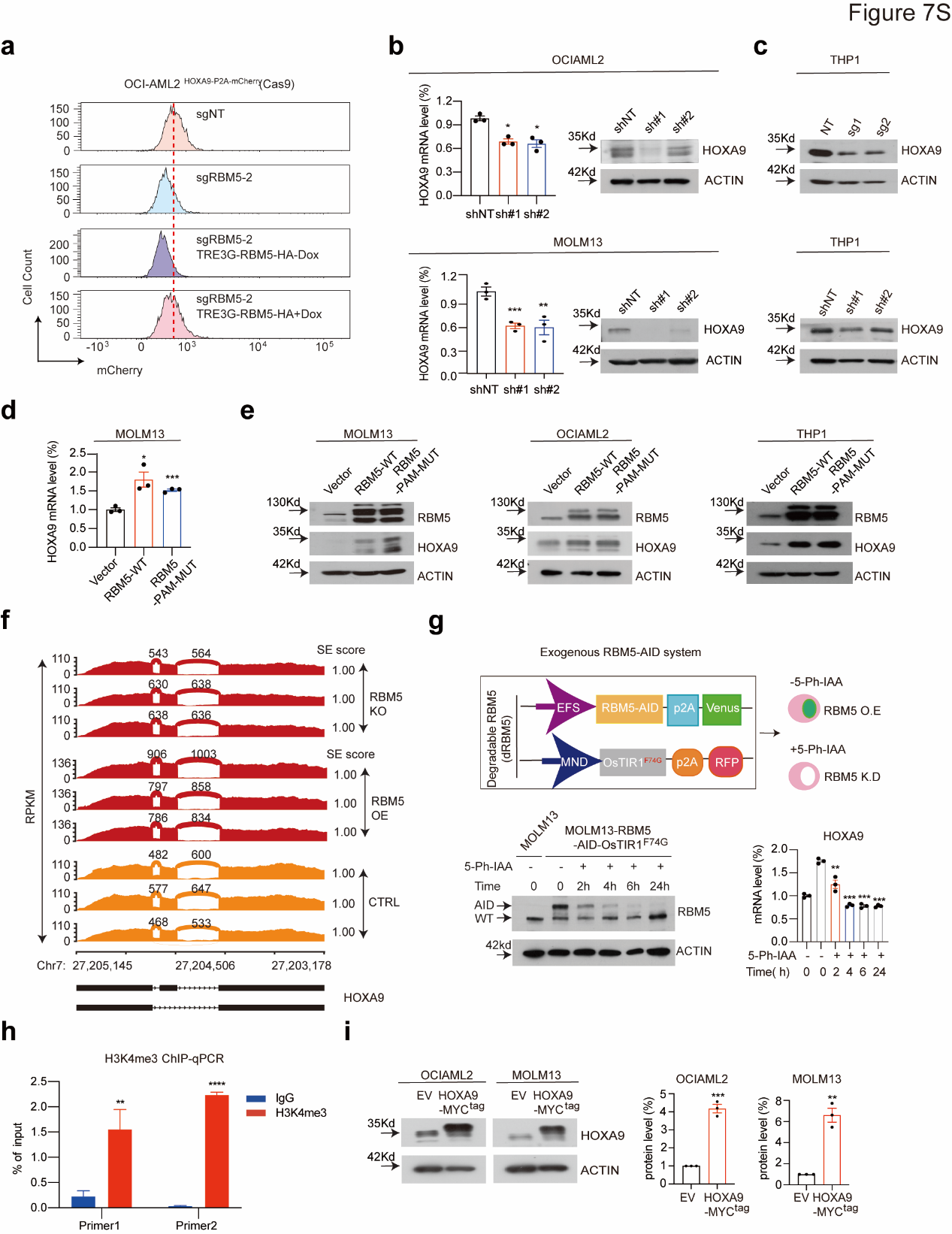


**Figure 7S. HOXA9 is a functional target gene of RBM5 in AML**

**a.** Flow cytometry analysis of the OCIAML2-HOXA9^P2A-mCherry^ cells targeted with non-target sgRNA (sgNT), RBM5-sgRNA-2 (sgRBM5), sgRBM5+TRE3G-RBM5-HA-DoX (RBM5-KO) and sgRBM5+TRE3G-RBM5-HA+DoX (RBM5-OE). **b**. Real-time-qPCR and Immunoblotting analysis was conducted on the shRBM5#1(sh#1), shRBM5#2(sh#2), and shNT targeted OCIAML2 and MOLM13 to monitor the expression of *HOXA9*. Data shown are means ± SEM from three independent experiments. * P < 0.05, ** P < 0.01, *** P < 0.001, unpaired Student’s t-test. **c.** Immunoblotting of HOXA9 in RBM5 sgRNAs and shRNAs targeted THP1. The β-ACTIN was used as a reference. **d.** Real-time-qPCR analysis was conducted by infecting MOLM13 cells overexpressing ectopic Venus empty vector, RBM5-WT, and RBM5-PAM-MUT. Data shown are means ± SEM from three independent experiments. * P < 0.05, *** P < 0.001, unpaired Student’s t-test. **e.** Immunoblotting was conducted by infecting MOLM13-Cas9, OCIAML2-Cas9 and THP1-Cas9 cells overexpressing ectopic Venus empty vector, RBM5-WT, and RBM5-PAM-MUT.  **f.** Sashimi plots the gene HOXA9 in RBM5 OE/CTRL and RBM5 KO/ CTRL group in MOLM13 cells. **g**. Schematic depicting the establishment of an inducible ectopic RBM5-AID degradation system in MOLM-13 cells. The overexpression of RBM5-AID can be acutely degraded through degron-mediated proteasome degradation with 5-Ph-IAA treatment. (Upper panel). The OsTIR1/mini-AID-mediated degradation efficiently decreased the RBM5-AID protein level after 2 hours of treatment, accompanied by a significant reduction of HOXA9 mRNA (Below panel). **h**. ChIP-qPCR with a H3K4me3 antibody for *HOXA9* locus in OCIAML2-RBM5-MYC^tag^ cells (n = 3). Statistical analysis (P value) was performed using an unpaired Student’s t-test. All error bars represent mean ± SEM. **i.** Immunoblotting was conducted by infecting OCIAML2 and MOLM13 cells overexpressing ectopic Venus empty vector and HOXA9 cDNA. **P < 0.01, ***P < 0.001, unpaired Student’s t-test.
