# Supplementary material for: RNA-binding protein RBM5 plays an essential role in acute myeloid leukemia by activating the oncogenic protein HOXA9
Source: Genome Biol. 2024 Jan 12;25:16. doi: 10.1186/s13059-023-03149-8 (PMC10785552; doi:10.1186/s13059-023-03149-8)
Supplement: Supplementary file 9 — Additional file 9. Uncropped images for the blots in Fig. 2, 3 and 4, Fig. 6 and 7 and supplementary Fig. 2, 3, 4, 7. [file 13059_2023_3149_MOESM9_ESM.docx]

**Fig2.c**

RBM5 (the upper red panel) and ACTIN (the below red panel) blot (MOLM13):


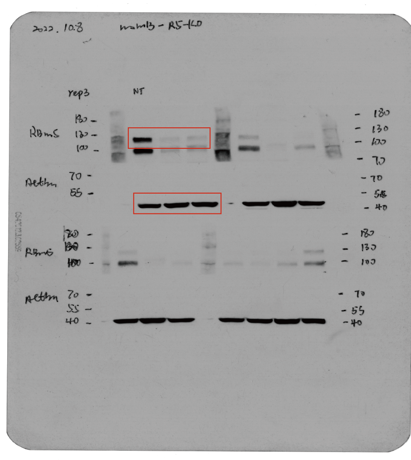


RBM5 (the upper red panel) and ACTIN (the below red panel) blot (THP1):

**
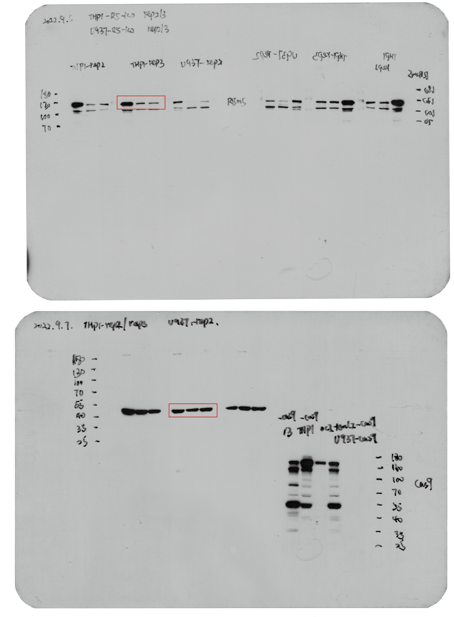
**

RBM5 (the left red panel) and ACTIN (the right red panel) blot (OCIAML2):

**
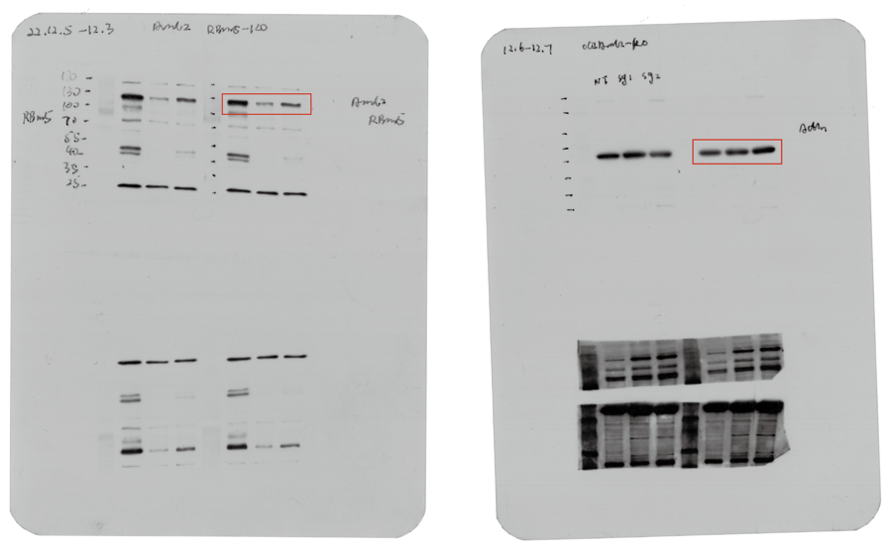
**

**Fig2.e**

RBM5 (the left red panel) and ACTIN (the right red panel) Blot

**
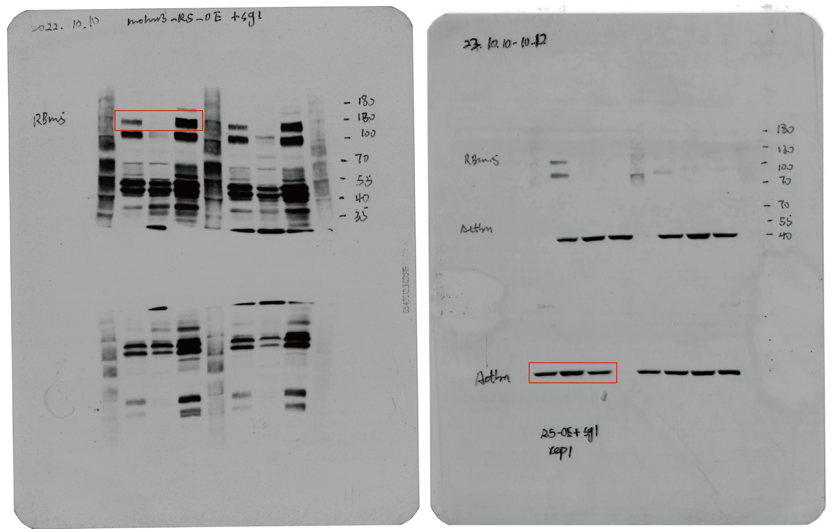
**

**Fig3.a**

RBM5 (the left red panel) and ACTIN (the right red panel) Blot (MOLM13):

**
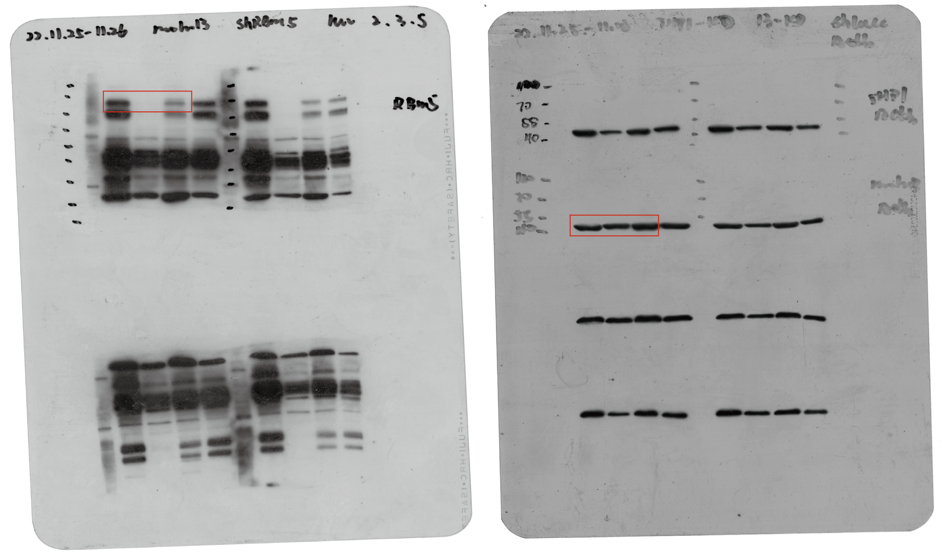
**

RBM5 (the left red panel) and ACTIN (the right red panel) Blot (OCIAML2):


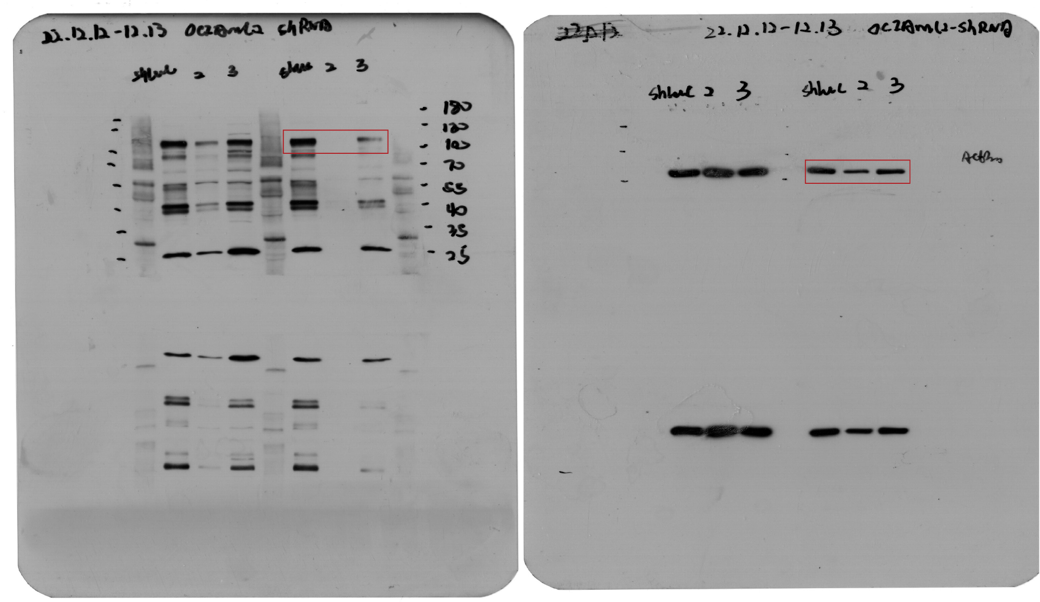


RBM5 (the left red panel) and ACTIN (the right red panel) Blot (THP1):

**
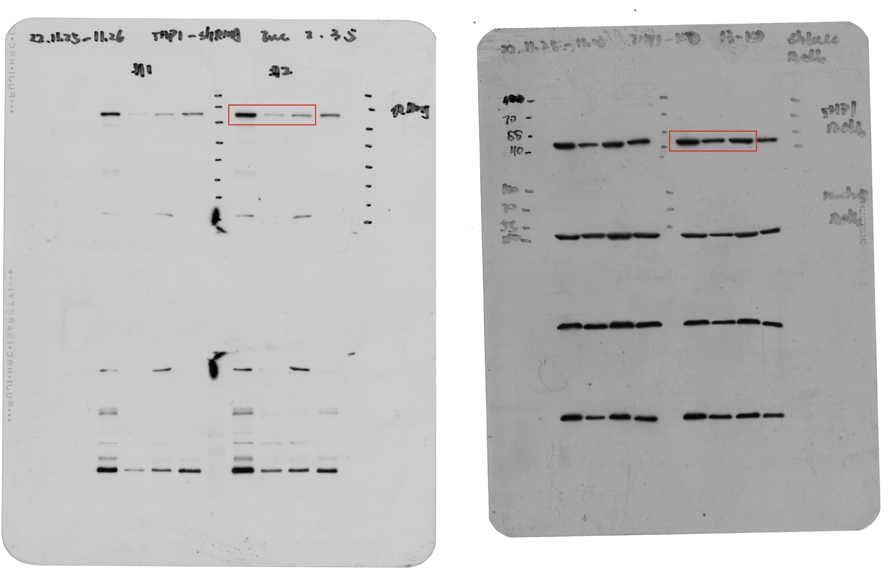
**

**Fig4.c**

RBM5 (the upper red panel)and ACTIN (the below red panel) Blot :

**
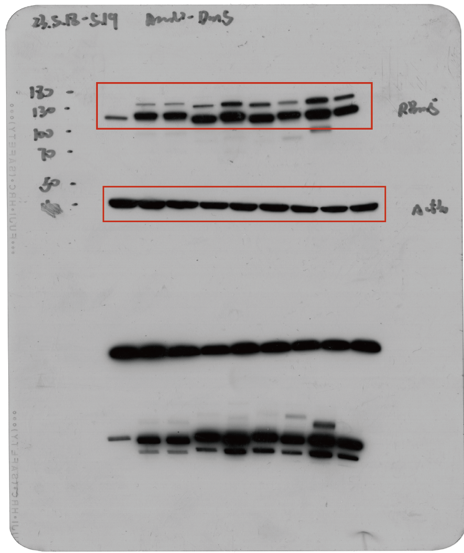
**

**Fig6.b**

RBM5 (the left red panel),ACTIN (the middle red panel) and HOXA9 (the right red panel) Blot

**
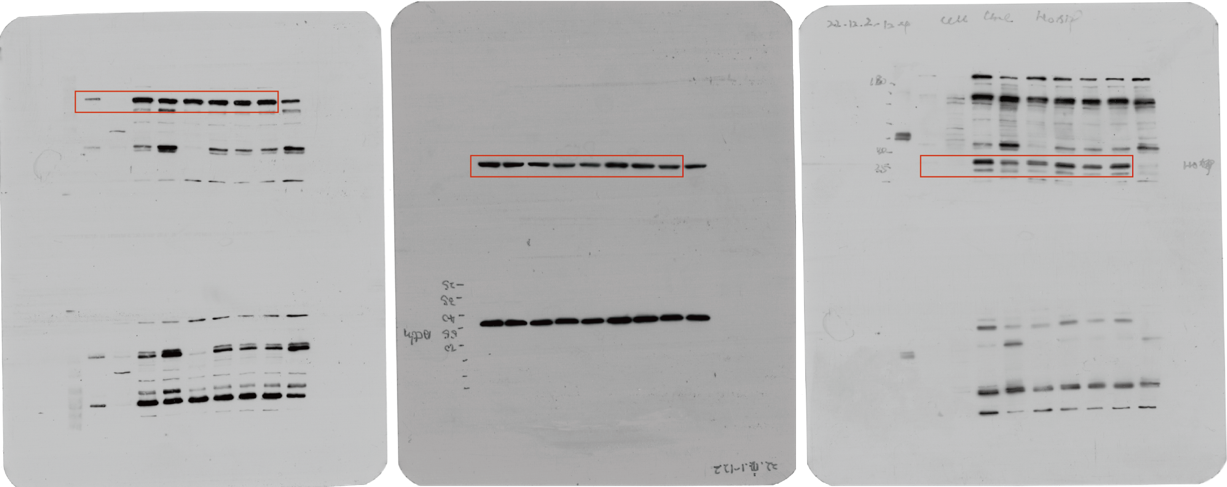
**

**Fig6.c**

HOXA9 (the left red panel) and ACTIN (the right red panel) Blot (OCIAML2)

**
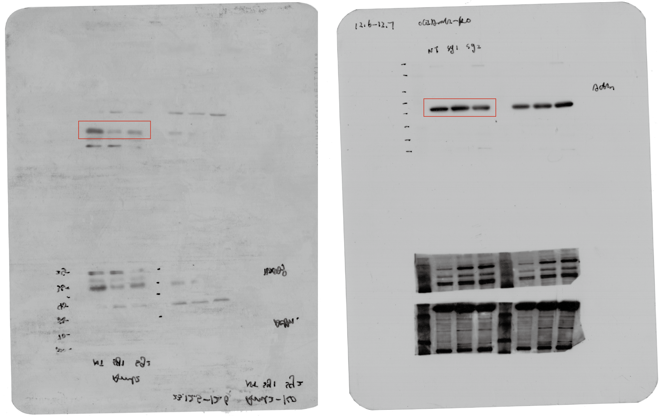
**

HOXA9 (the left red panel) and ACTIN (the right red panel) Blot (MOLM13)

**
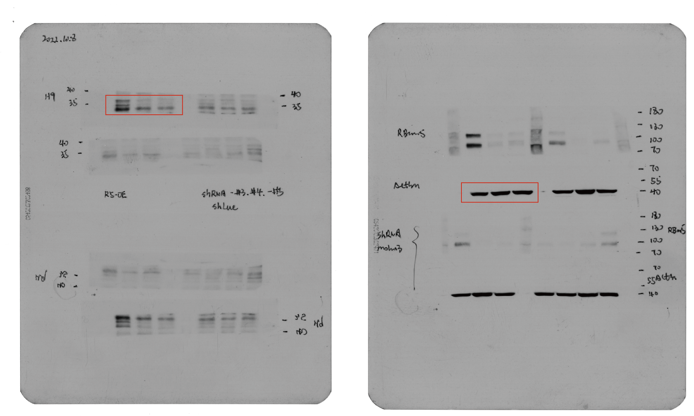
**

**Fig6.e**

RBM5 (the left red panel) and GAPDH (the right red panel) Blot

**
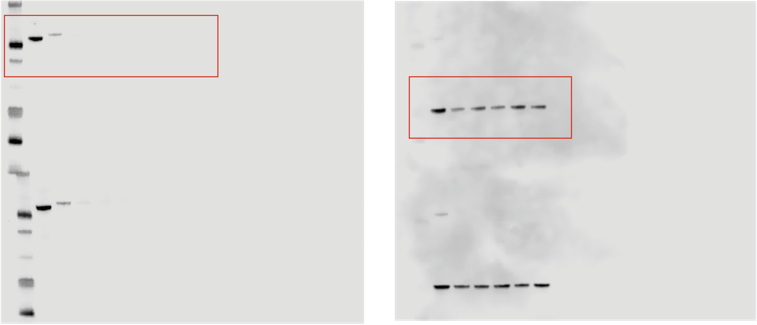
**

**Fig6.f**

RBM5 (the left red panel) and GAPDH ((the right red panel)) Blot

**
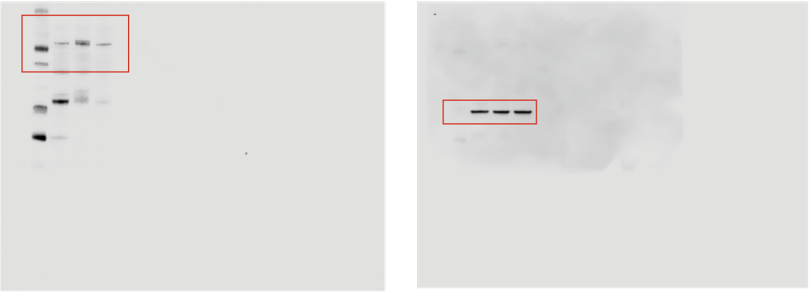
**

**Fig6.g**

HA-tag (the upper red panel), RBM5 (the middle red panel) and ACTIN (the below red panel) Blot

**
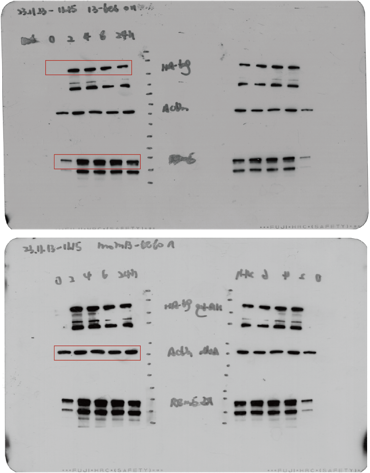
**

**Fig7.e**

HOXA9 (the left red panel), FLT3 (the right red panel)and ACTIN (the below red panel) Blot

**
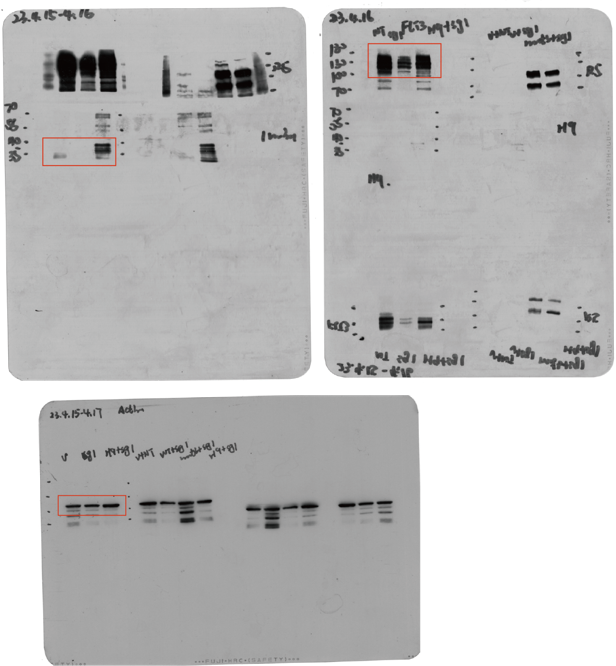
**

**Fig7.f**

ACTIN (the left red panel) and FLT3 (the right red panel) Blot

**
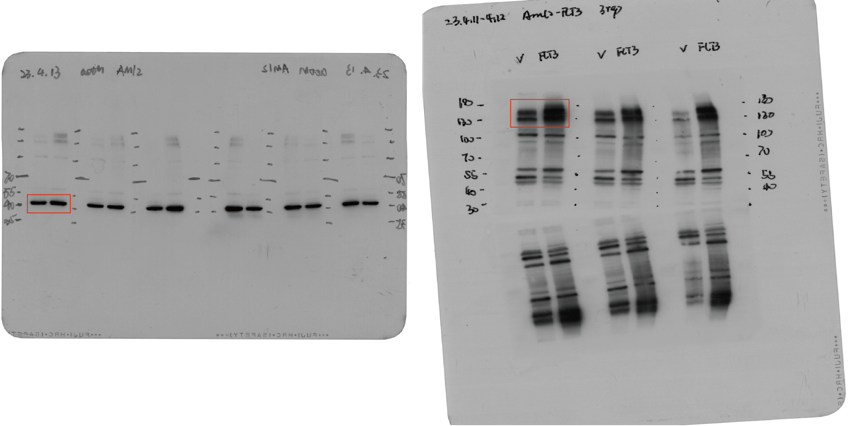
**

**Figure 2S.d**

RBM5 (the Left red panel) and ACTIN (the right red panel) Blot (U937):

**
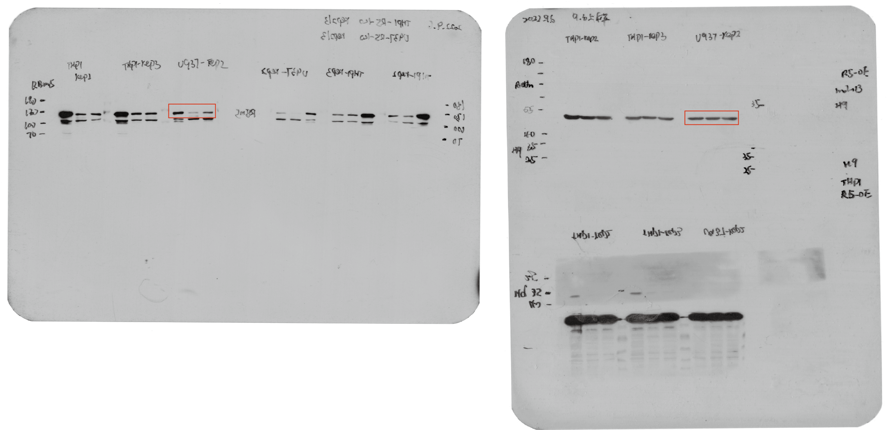
**

ACTIN (the Left red panel) and RBM5 (the right red panel) Blot (HEL and TF1):

**
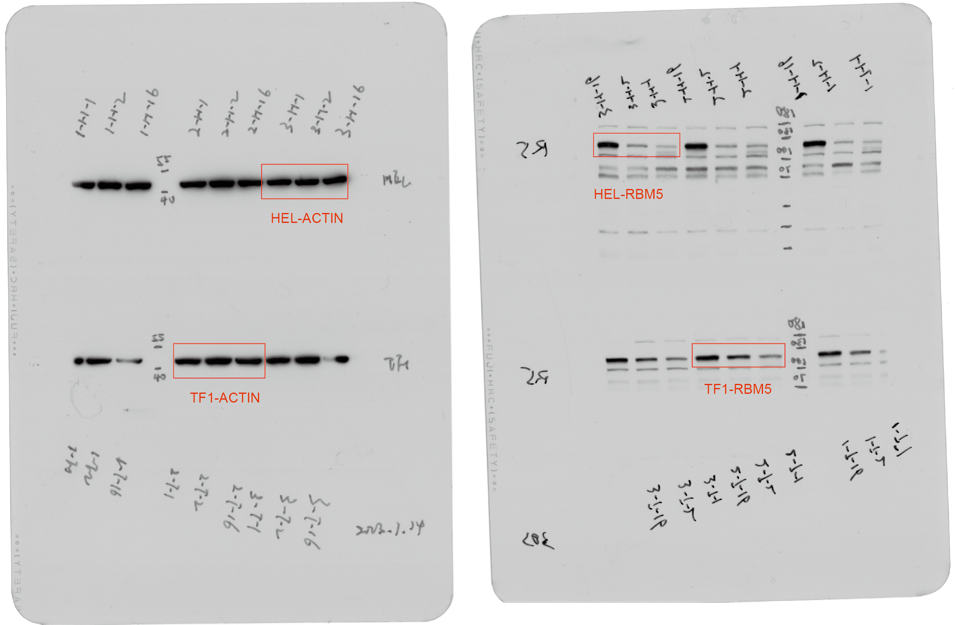
**

**Figure 2S.e**

RBM5 (the Left red panel) and ACTIN (the right red panel) Blot (THP1):

**
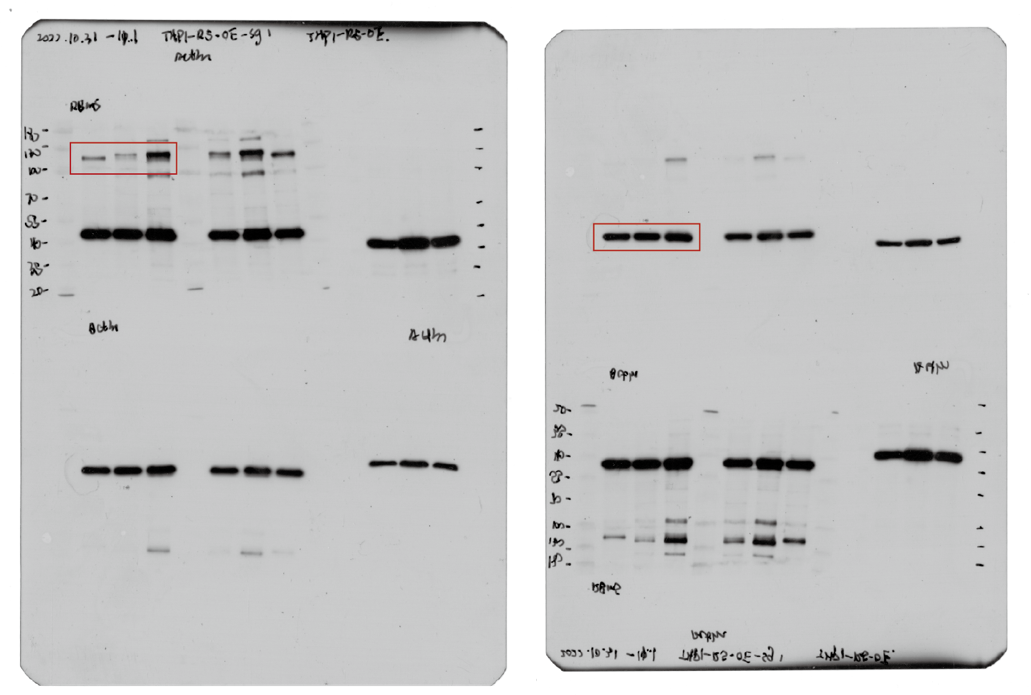
**

ACTIN (the Left red panel) and RBM5 (the right red panel) Blot (OCIAML2):

**
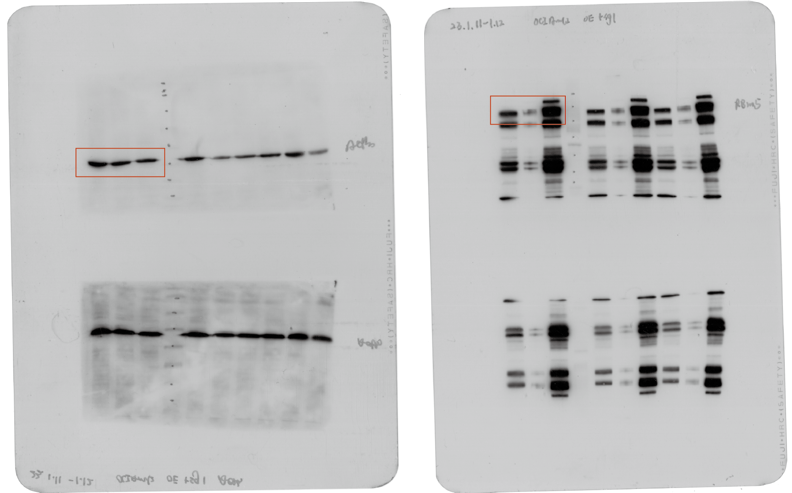
**

**Figure 3S.g**

RBM5 (the upper red panel) and ACTIN (the below red panel) Blot

**
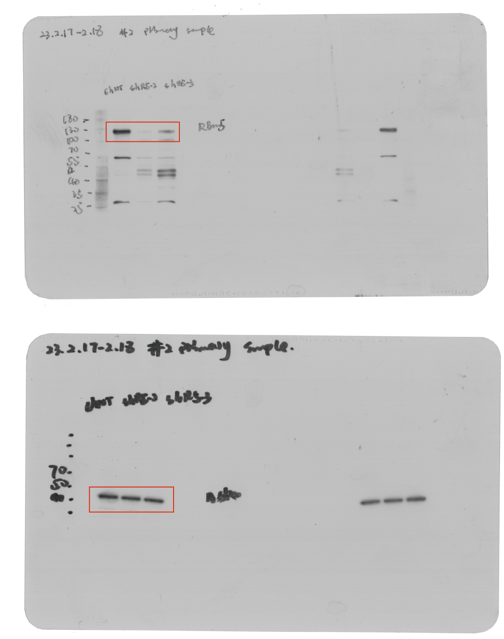
**

**Figure 4S.a**

RBM5 and ACTIN Blot

**
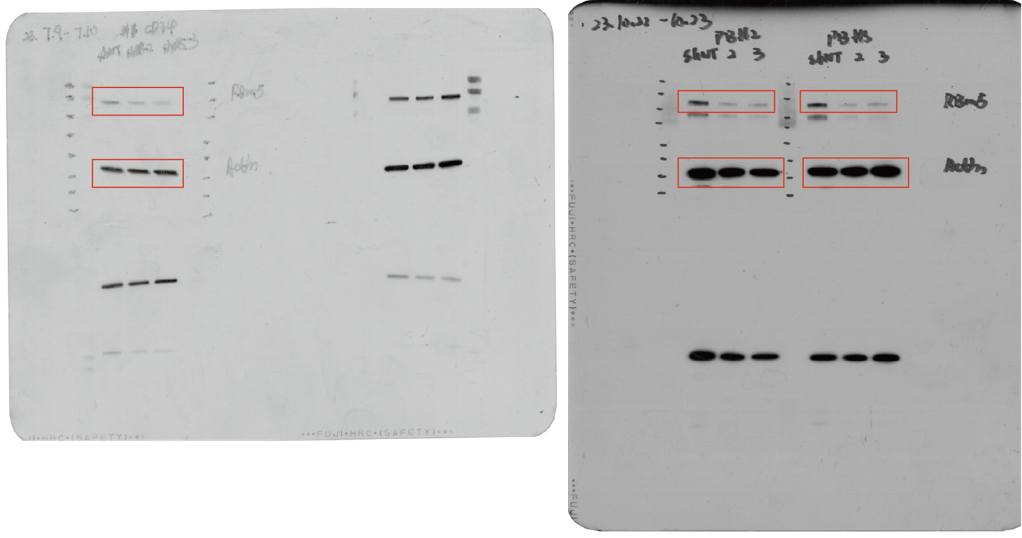
**

**Figure 4S.d**

RBM5 (the upper red panel) and ACTIN (the below red panel) Blot

**
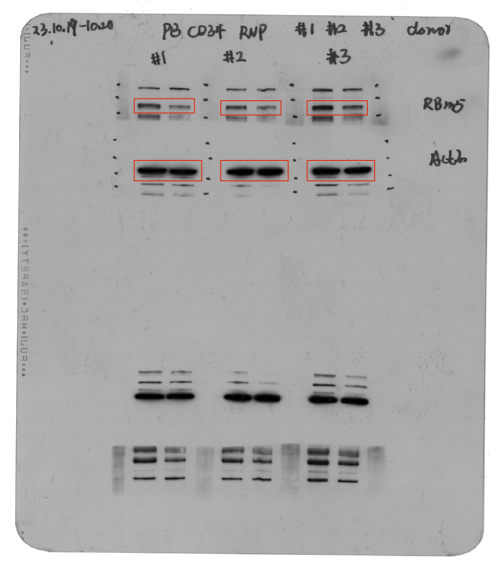
**

**Figure 7S.b**

HOXA9(the upper red panel) and ACTIN (the below red panel) blot (OCIAML2)

**
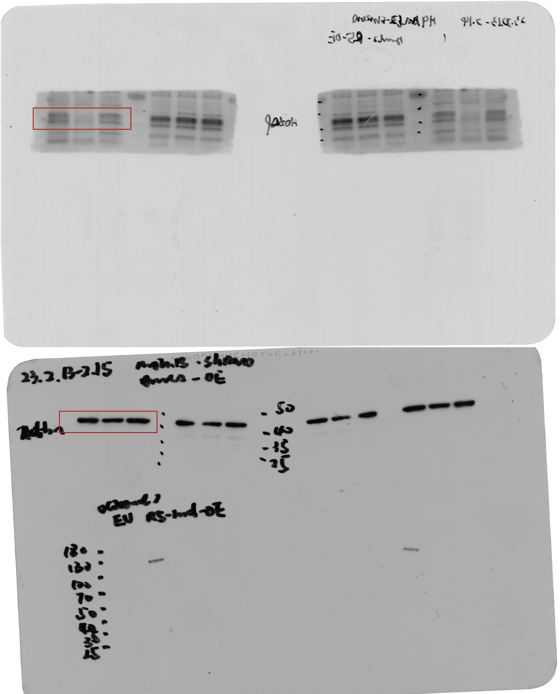
**

HOXA9(the upper red panel) and ACTIN (the below red panel) blot (MOLM13)

**
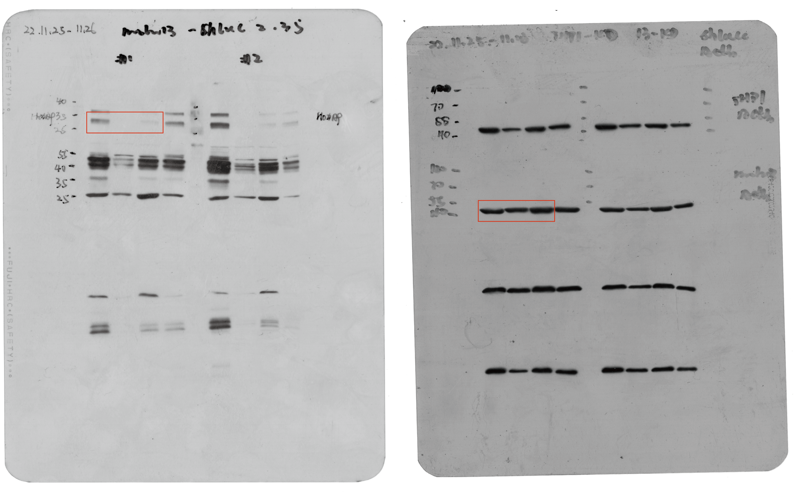
**

**Figure 7S.c**

HOXA9(the upper red panel) and ACTIN (the below red panel) blot (THP1)

**
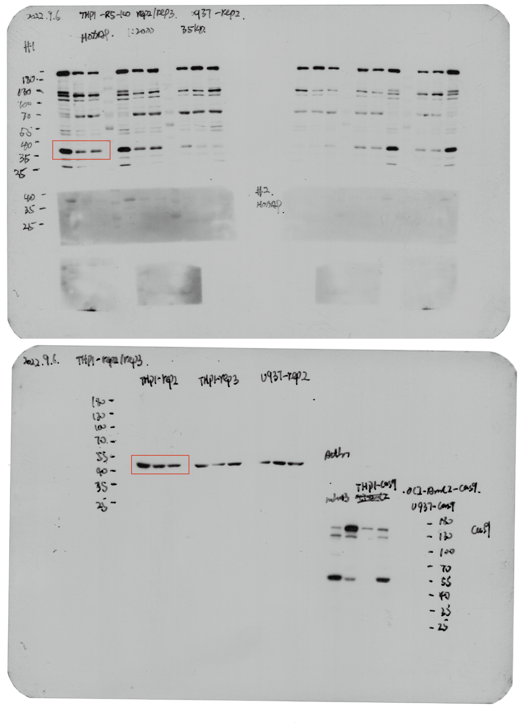
**

HOXA9(the upper red panel) and ACTIN (the below red panel) blot (THP1)

**
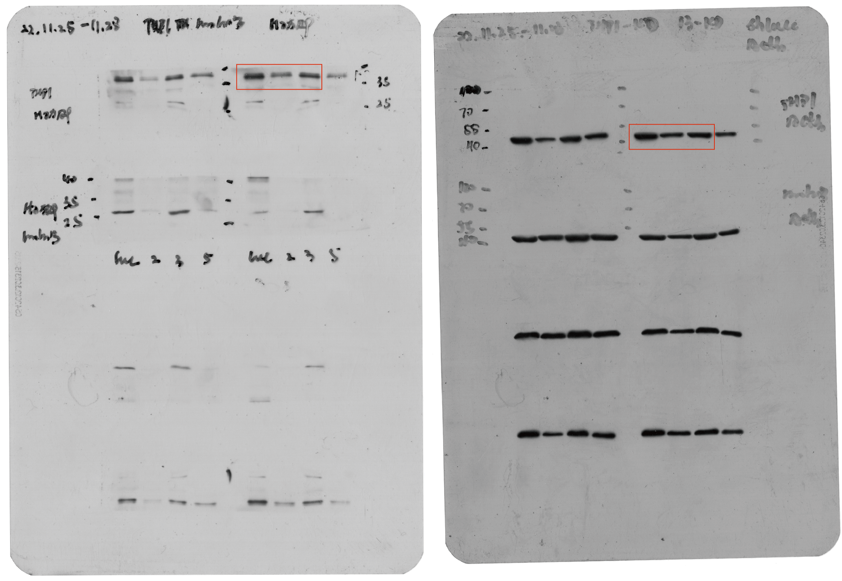
**

**Figure 7S.e**

RBM5 (the upper red panel) ,HOXA9 (the middle red panel) and ACTIN (the right red panel) Blot (MOLM13)

**
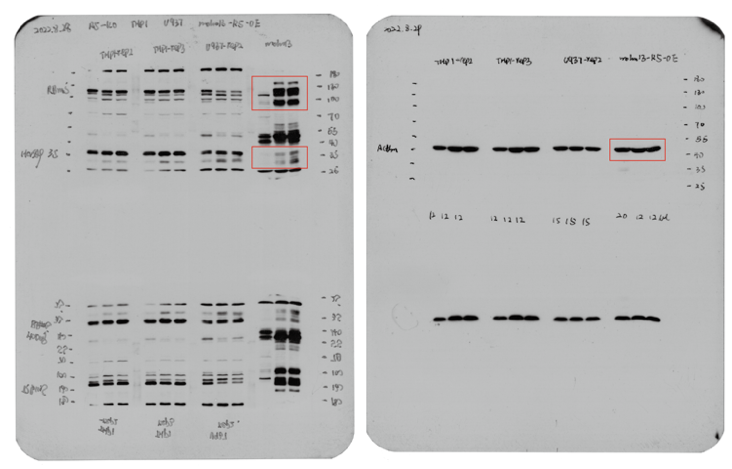
**

HOXA9 (the left red panel) ,RBM5 (the right red panel) and ACTIN (the below red panel) Blot (OCIAML2)

**
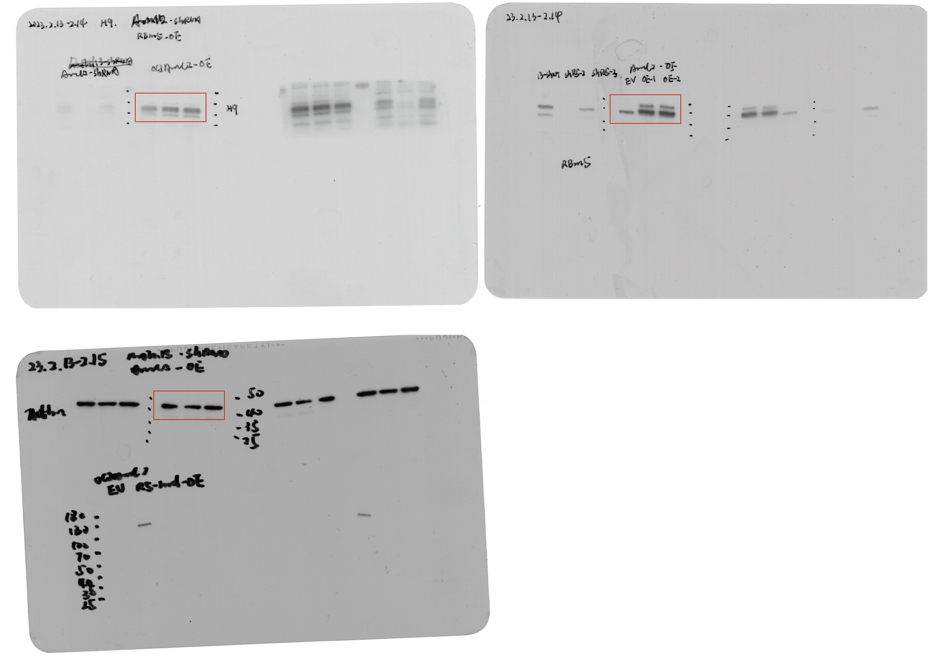
**

RBM5 (the upper red panel), ACTIN (the middle red panel) and HOXA9 (the below red panel) Blot (THP1)

**
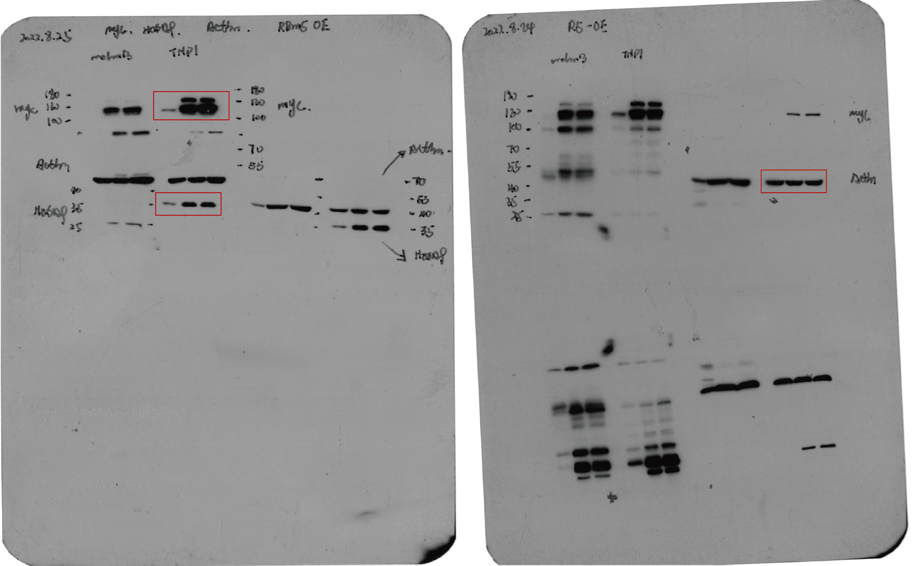
**

**Figure 7S.g**

RBM5 (the upper red panel) and ACTIN (the below red panel) Blot

**
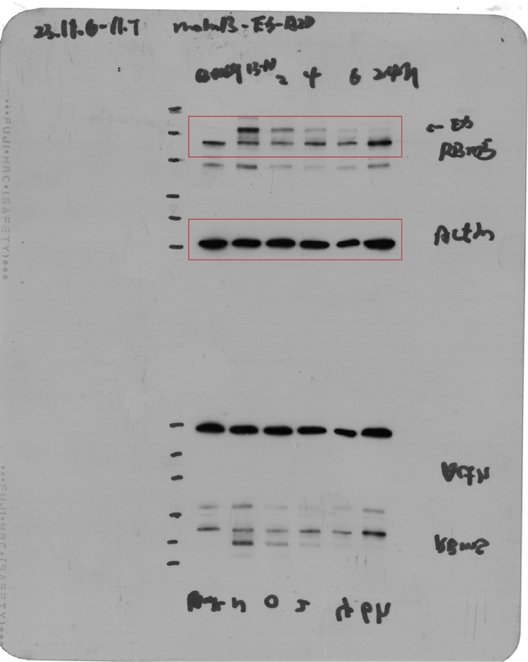
**

**Figure 7S.i**

HOXA9 (the left red panel) and ACTIN (the right red panel) blot

**
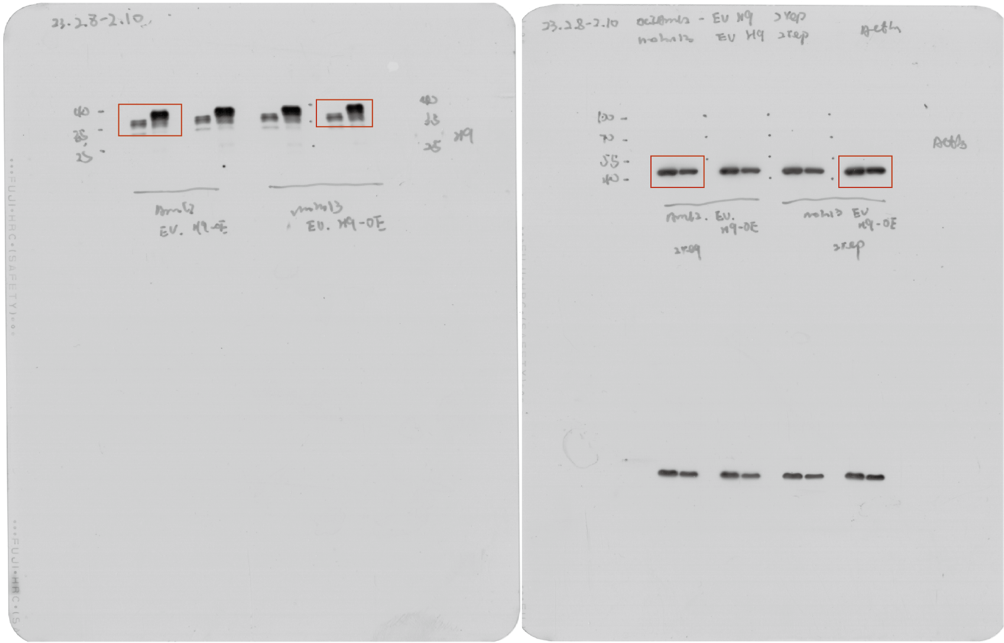
**
