# Supplementary material for: RNA-binding protein RBM5 plays an essential role in acute myeloid leukemia by activating the oncogenic protein HOXA9
Source: Genome Biol. 2024 Jan 12;25:16. doi: 10.1186/s13059-023-03149-8 (PMC10785552; doi:10.1186/s13059-023-03149-8)
Supplement: Supplementary file 10 — Additional file 10. Review history. [file 13059_2023_3149_MOESM10_ESM.docx]

Review History

**First round of review**

**Reviewer 1**

**Were you able to assess all statistics in the manuscript, including the appropriateness of statistical tests used?**

Yes, and I have assessed the statistics in my report.

**Comments to author:**

In this manuscript, Zhang et al., performed a CRISPR reporter screen to identify upstream regulators of HOXA9 in AML. Using this approach, the authors identified and focused on the splicing factor, RBM5 to show that it is required for AML survival in vitro and in vivo. To further elucidate the mechanistic basis of RBM5, the authors performed RNA-sequencing using gain- and loss-of-function approaches and found that perturbation of RBM5 led to changes in alternative splicing. The authors found that RBM5/HOXA9 expression correlated with FLT3 expression. Although this work is interesting, the mechanistic insight on how RBM5 is regulating HOXA9 is not clear. Moreover, there is a lack of understanding how RBM5 and HOXA9 is regulating FLT3. In addition, more experiments are required to better demonstrate RBM5 is dispensable for normal hematopoiesis to rationalize it as a potential AML target. These major and some minor points below need to be addressed before this paper is considered for publication.

Major points:

In Figure S3g, was the CFU only done at one time point (also the figure legends do not mention at what timepoint). The authors should also perform serial replating with normal hematopoietic cells to assess the effects of long-term RBM5 knockdown. Moreover, the authors performed many experiments in vivo using leukemia engraftment studies, can the authors also perform such in vivo experiments to knockdown RBM5 in normal hematopoiesis. It is important that RBM5 do not affect long-term HSCs.

The authors performed an HOXA9 reporter-based screen but did not show any validation of the top candidates. Can the authors confirm that loss of their top candidates lower HOXA9 expression using their reporter cell line and by HOXA9 mRNA expression?

The authors stated that RBM5 is selectively essential for AML cells but did not examine the effects of RBM5 ablation in other cancer cell lines. In the DepMap database, it seems lung adenocarcinoma and skin cancer are also required upon RBM5 deletion. Therefore, can the authors evaluate the effects of RBM5 in other cancer cell lines or remove the term "selectively".

The authors performed the reporter screen and most of the initial experiments with CRISPR/Cas9 but later changed to shRNA. What was the rationale for switching to different systems? Can the authors confirm the some of the findings from their shRNA experiments using CRISPR (e.g., RBM5 KO and HOXA9 expression, RBM5 KO induces AML differentiation)?

In Figure 5, the authors perform an extensive transcriptomic and splicing analysis of RBM5 perturbation but only concluded that there were no splicing changes associated with AML survival genes. Moreover, no splicing changes observed in HOXA9. Are any of the HOXA5 or HOXA9 targets mis-spliced upon RBM5 loss?This is currently the major area that is lacking which is how RBM5 regulating HOXA9. I highly recommend that the authors elucidate how RBM5 driving HOXA9 expression since this is the major takeaway from this paper.

Related to above, is RBM5 directly interacting with HOXA9 mRNA? This could help explain whether its regulation is direct or indirect (via another downstream gene).

Minor points:

HOXA9 expression has been shown to correlate with poor prognosis in AML, does RBM5 expression also correlate with poor survival?

For their AML verses normal comparison, what normal tissues were used from GTEx? Are these total bone marrow, HSC-enriched or PBMCs? A better description is needed for this analysis.

For Figures 4, 6, 7 and supplemental figures, it would be better to change RBM5 MUT to RBM5 sgRNA-resistant or use PAM-MUT to not confuse future reads that the MUT stands for a mutation generated from the cDNA constructs.

There is a misspelling of PWWPA3 on line 282.

**Reviewer 2**

**Were you able to assess all statistics in the manuscript, including the appropriateness of statistical tests used?**

No, I do not feel adequately qualified to assess the statistics.

**Comments to author:**

High level HOXA9 is important for stem cell expansion and is associated with certain subtypes of AML (MLL rearranged, Nup98-HOXA9, NPMc). Therefore the mechanisms of how HOXA9 is regulated, including by upstream transcription factors is important. This study uses a genome wide CRISPR/Cas9 screen of a HOXA9 reporter leukemia cell line along with a variety of correlative and loss of function and gain of function approaches to establish an important role for the RNA-binding protein RPM5 in for upregulating HOXA9 expression. Of note, RBM5 is highly expressed in AML cell lines and tends to be correlated with HOXA9 expression.

The work is thorough, looking at both cell lines and primary leukemia cells, in vitro and in and the data clearly support a role for RPM5 in upregulating HOXA9 expression, and also this is important for leukemia cell proliferation. This paper should be of interest to those studying mechanisms of leukemogenesis as well as non-canonical roles for splicing factors.

Comments:

1. What remains an open question is whether the mechanism of HOXA9 upregulation is direct regulation of transcription as shown in Figure 8, or indirect through some other mechanism. This is the main conclusion of the manuscript. The authors make an argument that it is not a change in RNA splicing that is involved, and also that the RBM5 DNA binding domain is involved, but it seems possible that a change in splicing in some other transcription factor than RPM5 might be involved. This issue could be effectively addressed by ChIP for RBM5 which would establish whether RBM5 actually binds to the HOXA9 locus.

2. Figure 4b-4d- It looks like all the mutated domains result in decreased HOXA9 expression. The authors should acknowledge the possibility that this is lost of function because of an allosteric effect.

3. Fig. 3K- if the authors have morphology (Wright-Giemsa stained cells) on the cells in Fig 3d and 3k this should be included.

4. Page 9, line 344- regarding the expression of FLT3 is regulated by RBM5/HOXA9, the authors should be aware that Flt3 has been shown to be a directly upregulated target of Hoxa9, at least in murine cells (Huang et al. Blood 2012 Jan 12; 119(2): 388-398). It would be beneficial to cite this.

5. In a number of places the manuscript needs some editing for word choice and syntax. For example, it is difficult to determine what is meant about "lame characterization" line 196 on page 6.

**Authors Response**

**Point-by-point responses to the reviewers’ comments:**

Reviewer #1:

In this manuscript, Zhang et al., performed a CRISPR reporter screen to identify upstream regulators of HOXA9 in AML. Using this approach, the authors identified and focused on the splicing factor, RBM5 to show that it is required for AML survival in vitro and in vivo. To further elucidate the mechanistic basis of RBM5, the authors performed RNA-sequencing using gain- and loss-of-function approaches and found that perturbation of RBM5 led to changes in alternative splicing. The authors found that RBM5/HOXA9 expression correlated with FLT3 expression. Although this work is interesting, the mechanistic insight on how RBM5 is regulating HOXA9 is not clear. Moreover, there is a lack of understanding how RBM5 and HOXA9 is regulating FLT3. In addition, more experiments are required to better demonstrate RBM5 is dispensable for normal hematopoiesis to rationalize it as a potential AML target. These major and some minor points below need to be addressed before this paper is considered for publication.

*Response: Thank you for your suggestions. To elucidate how RBM5 drives HOXA9 expression, we utilized two major strategies to understand whether RBM5 controls HOXA9 transcription directly or indirectly (See next page： new Fig. 6 and Additional file 1: Fig. S7). We added this part of the results in the revised manuscript (lines 322-347, second paragraph of page 9; lines 353-357, first paragraph of page 10).*

*Firstly, we reasoned that the direct regulation of HOXA9 gene transcription would occur in an acute manner. Therefore, we utilized the auxin-induced degron (AID) system and generated two endogenous*

*RBM5 N-terminus AID knock-in KMT2A-r leukemia lines (SEM homozygous clones and MOLM13 bulk cells). Those cell lines were then transduced by lentiviral vectors constitutively expressing OsTIR1(F74G) [1,2]. With the presence of small molecule 5-Ph-IAA, the OsTIR1(F74G), as a substrate receptor in Skp1, Cullin, F-box (SCF) complex, could target and mediate specific AID-tagged proteins for ubiquitination and degradation (in the revised Fig. 6d). Of note, after 2 hours post-treatment, the immunoblotting analysis*

*showed that the AID-RBM5 fusion protein was undetectable. Consequently, the HOXA9 mRNA level was significantly reduced in two SEM knock-in clones after 2 hours of treatment and remained low until 24 hours (in the revised Fig. 6e). Similar effect was also observed after 24 hours of 5-Ph-IAA treatment in MOLM13 knock-in bulk populations (in the revised Fig. 6f). While conversely, we utilized an inducible overexpression system with the TRE3G promoter in the MOLM13 cell line. With the treatment of doxycycline for 2 hours and after that, we found significant upregulation of HOXA9 mRNA level accompanied by the increase of RBM5 expression (in the revised Fig. 6g). Similar effects were observed by overexpressing an exogenous RBM5-N terminus AID fusion protein (in the revised Additional file 1: Fig. S7g). Strikingly, upon the addition of 5-Ph-IAA in the presence of OsTIR1 (F74G), a significant reduction of HOXA9 mRNA was observed after 2 hours of treatment and after that (in the revised Additional file 1: Fig. S7g). Taken together, those results indicate that RBM5 directly activated the HOXA9 transcription.*

*Secondly, given that RBM5 regulates the transcriptional level of HOXA9 through both RNA and DNA binding domains, we hypothesized that RBM5 may directly bind to the HOXA9 gene locus. To test this, we performed an anti-MYC tag antibody ChIP (Chromatin Immunoprecipitation) to analyze the chromatin occupancy of exogenous RBM5-MYC expressed in OCIAML2 leukemia cells. Importantly, we observed significant enrichment of RBM5-MYC on the downstream region of HOXA9, together with active transcription mark H3K4me3 (in the revised Fig. 6i; Additional file 1: Fig. S7h). These results suggest that RBM5 occupied the HOXA9 gene locus and directly activated its transcription.*

*New Fig. 6. HOXA9 is a functional target gene of RBM5 in AML. a. Scatter plot analysis comparing RBM5 mRNA levels and HOXA9 mRNA levels across acute myeloid leukemia cell lines (data was obtained from the Depmap). P value is calculated by linear regression. b. Immunoblotting of RBM5 and HOXA9 in various leukemia cell lines and normal bone marrow (NBM) samples (left panel). The bar chart displays the quantitative protein levels of RBM5 and HOXA9 in various cell lines depicted in the left panel (right panel). c. Real-time-qPCR and Immunoblotting analysis was conducted on the RBM5-sg1, RBM5- sg2, and sgNT targeted MOLM13 and OCIAML2 to monitor the expression of HOXA9. Data shown are means ± SEM from three independent experiments. * P < 0.05, unpaired Student’s t-test. d. Diagram of auxin-induced degron (AID) system to degrade endogenous RBM5. Endogenous RBM5 N-terminus AID knock-in KMT2A-r leukemia lines (SEM homozygous clones and MOLM13 bulk cells) were followed by constitutively expressing OsTIR1(F74G). With the presence of small molecule 5-Ph-IAA, the OsTIR1(F74G), as a substrate receptor in Skp1, Cullin, F-box (SCF) complex, could target and mediate specific AID-tagged RBM5 proteins for ubiquitination and degradation. e. The protein level of endogenous RBM5-AID of SEM homozygous clones can be acutely degraded with 5-Ph-IAA through degron-mediated proteasome degradation after 2 hours post-treatment (Left panel). HOXA9 mRNA level was significantly reduced after 2 hours, 4 hours, 6 hours, and 24 hours of treatment in SEM cells (Right panel). f. After 24 hours of treatment with 5-Ph-IAA in HA-miniAID-RBM5 knock-in cells, the protein level of Knockin-RBM5 decreased (Left panel), and there was also a significant reduction in HOXA9 mRNA level (Right panel). g. The doxycycline-Tet-On system for ectopic overexpression of RBM5-HA in the MOLM13 cell line, after a 2-hour treatment with doxycycline, a significant upregulation in HOXA9 mRNA levels was observed (Right panel), accompanied by an increase in RBM5 expression (Left panel). h. Real-time-qPCR analysis was conducted in OCIAML2 cells transduced with RBM5 cDNA wildtype (WT) or six individual truncated mutants to validate the expression of HOXA9. Data shown are means ± SEM from three independent experiments. *P < 0.05, unpaired Student’s t-test. i. ChIP-qPCR with anti-MYC tag antibody near H3K4me3-bound HOXA9 downstream region (Chr7:27,199,969-27,200,853) in OCIAML2-RBM5-MYCtag cells (n=3). The red arrows indicate the primer 1 and primer 2 located position, and the blue arrow indicates the negative control (NC) primer located position, where there is no H3K4me3 signal. Statistical analysis (P value) was performed using an unpaired Student’s t-test. All error bars represent mean ± SEM. j. Rescued competitive proliferation assay was conducted by infecting OCIAML2-Cas9 and MOLM13-Cas9 cells overexpressing ectopic RBM5-wild type cDNA (WT), RBM5-sgRNA1 resistant mutant cDNA (PAM-MUT), and HOXA9 cDNA (linked to Venus reporter) with lentiviral-sgRNAs against non-target (sgNT) and RBM5 (RBM5-sg1) at about 50% efficiency (all monitored by Venus reporter). Data shown are means ± SEM from three independent experiments. ** P < 0.01, unpaired Student’s ttest.*

*Additional file 1: Fig. S7. HOXA9 is a functional target gene of RBM5 in AML. a. Flow cytometry analysis of the OCIAML2-HOXA9P2A-mCherry cells targeted with Nontarget-sgRNA (sgNT), RBM5-sgRNA-2 (sgRBM5), sgRBM5+TRE3G-RBM5-HA-DoX (RBM5-KO) and sgRBM5+TRE3G-RBM5-HA+DoX (RBM5- OE). b. Real-time-qPCR and Immunoblotting analysis was conducted on the shRBM5#1(sh#1), shRBM5#2(sh#2), and shNT targeted OCIAML2 and MOLM13 to monitor the expression of HOXA9. Data shown are means ± SEM from three independent experiments. * P < 0.05, ** P < 0.01, *** P < 0.001, unpaired Student’s t test. c. Immunoblotting of HOXA9 in RBM5 sgRNAs and shRNAs targeted THP1. The β-ACTIN was used as a reference. d. Real-time-qPCR analysis was conducted by infecting MOLM13 cells overexpressing ectopic Venus empty vector, RBM5-WT, and RBM5-PAM-MUT. Data shown are means ± SEM from three independent experiments. * P < 0.05, *** P < 0.001, unpaired Student’s t-test. e. Immunoblotting was conducted by infecting MOLM13-Cas9, OCIAML2-Cas9 and THP1-Cas9 cells overexpressing ectopic Venus empty vector, RBM5-WT, and RBM5-PAM-MUT. f. Sashimi plots the gene HOXA9 in RBM5 OE/CTRL and RBM5 KO/ CTRL group in MOLM13 cells. g. Schematic depicting the establishment of an inducible ectopic RBM5-AID degradation system in MOLM-13 cells. The overexpression of RBM5-AID can be acutely degraded through degron-mediated proteasome degradation with 5-Ph-IAA treatment. (Upper panel). The OsTIR1/mini-AID-mediated degradation efficiently decreased the RBM5-AID protein level after 2 hours of treatment, accompanied by a significant reduction of HOXA9 mRNA (Below panel). h. ChIP-qPCR with a H3K4me3 antibody for HOXA9 locus in OCIAML2-RBM5-MYCtag cells (n = 3). Statistical analysis (P value) was performed using an unpaired Student’s t-test. All error bars represent mean ± SEM. i. Immunoblotting was conducted by infecting OCIAML2 and MOLM13 cells overexpressing ectopic Venus empty vector and HOXA9 cDNA. **P < 0.01, ***P < 0.001, unpaired Student’s t-test.*

*However, we could not rule out whether RBM5 occupancy on the HOXA9 locus depends on other transcription factors. To understand whether the RBM5 regulates HOXA9 in an “indirect manner,” such as by changing the splicing events of a particular upstream transcription factor, we performed RNA splicing events analysis on the top candidate TFs that regulate HOXA9 from previous publication [3]. Of note, we found most of the tested TFs did not exhibit splicing changes, while only a significant splicing event was observed in USF2 after RBM5 loss in AML cell lines (See below: Response Figure R1.A). Given the importance of USF2 in regulating HOXA9 expression, we validated whether the mRNA level of USF2 changed after RBM5 reduction. Of note, both the mRNA and protein levels of USF2 were not significantly changed after RBM5 loss (See below: Response Figure R1.B). Therefore, these results suggest that RBM5 might not regulate HOXA9 expression by altering the RNA splicing of particular transcription factors.*

*Regarding how RBM5 and HOXA9 regulate FLT3, we have demonstrated that FLT3 mRNA and protein level was reduced after RBM5 knockout (in the original Fig. 7d and 7e). It is known that HOXA9 directly occupies the FLT3 enhancer region and activates its transcription[4]. This is consistent with our evidence that HOXA9 could restore the FLT3 expression in RBM5 null leukemia cells (in the original Fig. 7d and 7e). Moreover, to test whether RBM5 directly regulates FLT3 transcription, we added the ChIP-qPCR validation on the FLT3 gene locus. The results indicate that RBM5 did not directly bind on the promoter of FLT3 (See below: Response Figure R1.C). Taken together, our data suggests that RBM5 may activate FLT3 through upregulating HOXA9.*

*Regarding the point on whether RBM5 is dispensable for normal hematopoiesis, we now added more validation in normal primary CD34+ hematopoietic stem and progenitor cells by both RNAi-mediated knockdown and CRISPR/Cas9-mediated knockout strategies as described in major point 1 in below.*

*Response Figure R1. USF2 and FLT3 analysis. A. Sashimi plots the gene USF2 in the RBM5 KO/ CTRL group in MOLM13 cells. B. Real-time-qPCR and Immunoblotting analysis was conducted on the sgRBM5, and sgNT targeted OCIAML2 to monitor the expression of USF2. Data shown are means ± SEM from three independent experiments. C. The left panel represents a schematic diagram of the FLT3 gene locus. The arrows indicate its promoter region. The right panel shows ChIP-qPCR with an MYCtag antibody near the FLT3 gene promoter in OCIAML2-RBM5-MYCtag cells (n=3). Statistical analysis (P value) was performed using an unpaired Student’s t-test. All error bars represent mean ± SEM.*

Major points:

In Figure S3g, was the CFU only done at one time point (also the figure legends do not mention at what timepoint). The authors should also perform serial replating with normal hematopoietic cells to assess the effects of long-term RBM5 knockdown. Moreover, the authors performed many experiments in vivo using leukemia engraftment studies, can the authors also perform such in vivo experiments to knockdown RBM5 in normal hematopoiesis. It is important that RBM5 do not affect long-term HSCs.

*Response: Thank you for the reviewer’s comments. We added the time point “day 14” in the figure and figure legends. In addition, we added more validation experiments by both shRNA-mediated knockdown and CRISPR/Cas9-mediated knockout strategies. We merged all the normal hematopoiesis validation data in a separate supplemental figure (See next page: Additional file 1: Fig. S4).*

*Regarding the suggestions for serial replating with normal hematopoietic cells to assess the effects of long-term RBM5 knockdown, we have repeated the CFU assay in normal CD34+ HSPCs from one additional normal donor, together with a previous donor. After 14 days of in vitro culture, we observed similar results from two independent donors. We have updated the figure by merging three independent normal donors (See below: Additional file 1: Fig. S4a-c). To further perform serial replating, we collected the bulk cells from all the colonies from two donors (PB#002 and PB#003) and measured the percentage of CD34+ cells in each group. Of note, the remaining CD34+ cells were comparable between the control and RBM5 knockdown groups, suggesting the HSPCs compartment was unaffected (See below: Response Figure R2.A). We further set up a second round of CFU assay. After another 14 days of culture, the number of colonies in the RBM5 knockdown group was similar to that in the control group, suggesting that the suppression of RBM5 did not affect the long-term survival of hematopoietic cells (See below: Response Figure R2.B). Moreover, we also utilized the CRISPR/Cas9 ribonucleoprotein (RNP) strategy to knockout RBM5 in normal CD34+ HSPCs from three independent donors and performed the CFU assay. The RBM5 deletion by CRISPR/Cas9 did not alter the suspension myeloid differentiation or the formation of myeloid or mixed-lineage colonies (See below: Additional file 1: Fig. S4d-f).*

*We agreed with the reviewer that the ideal assay for measuring normal hematopoiesis would be in vivo transplantation or genetic model and discussed the limitations of in vitro culture experiments in the revised manuscript (lines 228-231, first paragraph of page 7; lines 432-440, last paragraph of page 11). It was reported that the non-sense mutation on the RRM domain of RBM5 in mouse models exhibited defects in sperm differentiation but not hematopoiesis [5]. More recent studies revealed that RBM5 is critical in neuronal injury response and participates in mouse models of Huntington’s disease [6,7]. However, it remains unknown whether disruption of the zinc finger domains or loss of the whole protein causes hematopoietic defect by in vivo genetic or transplantation models, which requires further comprehensive evaluation. We argue that it is beyond the scope of this research but needs further comprehensive study in the future.*

*Additional file 1: Fig. S4. RBM5 suppression does not affect human normal hematopoiesis. a. Immunoblotting of RBM5 in normal PB (#001-#003) HSPCs with and without RBM5 knockdown. b. Flow cytometry analysis of the percentage of myeloid markers CD11b and CD14 in day 14 myeloid expansion cultures. The bar graph displays the percentage of double-positive cells for CD11b and CD14, with three parallel replicates from three normal donors. c. Count of colonies in CFU assay in day 14 cultures from transduced CD34+ HSPCs with and without RBM5 knockdown, with three parallel replicates from three normal donors. d. Immunoblotting was performed to analyze RBM5 expression in normal PB (#001-003) HSPCs with and without RBM5 knockout. e. Flow cytometry analysis was conducted to evaluate the percentage of myeloid markers CD11b and CD14 in day 14 myeloid expansion cultures. The accompanying bar graph illustrates the percentage of CD11b and CD14 double-positive cells, with data derived from three parallel replicates from three normal donors. f. Counting colonies in the CFU assay was carried out on day 14 cultures derived from transduced CD34+ HSPCs, comparing those with and without RBM5 knockout. The experiment included three parallel replicates sourced from three different normal donors.*

*Response Figure R2. A. Representative flow cytometry analysis to measure the percentage of CD34+ cells in each group cells from two donors (PB#002 and PB#003), MFI: mean fluorescence intensity. B. Count colonies in CFU assay in serial plating after the second round of day 14 cultures from the equal number of bulk cells with and without RBM5 knockdown from two normal donors.*

The authors performed an HOXA9 reporter-based screen but did not show any validation of the top candidates. Can the authors confirm that loss of their top candidates lower HOXA9 expression using their reporter cell line and by HOXA9 mRNA expression?

*Response: Thank you for the reviewer’s comments. First, we added the validation data of RBM5 on the OCIAML2 HOXA9-mCherry reporter lines. Notably, the fluorescence signal was reduced after RBM5 sgRNA treatment and could be restored by inducible RBM5 overexpression (See below: Additional file 1: Fig. S7a).*

*Beyond the top hit RBM5, we randomly picked up other top candidates (DHX15, STAG2, MED30, and GTF2A2) for validation. We now performed more validation data for genes that are among the top 100 candidates from our screen dataset, including MED30 (Rank17), DHX15 (Rank41), GTF2A2 (Rank69), 9 and STAG2 (Rank79). Importantly, individual sgRNAs targeting those candidates significantly decreased the HOXA9 mRNA level compared to the non-target sgRNA control in a separate KMT2A-r cell line MOLM13. Moreover, we also confirmed their cellular growth defect by competitive proliferation assay in MOLM13. The newly added data was included (See below: Additional file 1: Fig. S1b and S1c).*

*Additional file 1: Fig. S7a. Flow cytometry analysis of the OCIAML2-HOXA9P2A-mCherry cells targeted with Nontarget-sgRNA (sgNT), RBM5-sgRNA-2 (sgRBM5), sgRBM5+TRE3G-RBM5-HA-DoX (RBM5- KO) and sgRBM5+TRE3G-RBM5-HA+DoX (RBM5-OE).*

*Additional file 1: Fig. S1 b. Real-time-qPCR analysis was conducted on the sgGTF2A2, sgSTAG2, sgMED30, or sgDHX15 targeted MOLM13-Cas9 cell to monitor the mRNA of HOXA9. Non-target (NT) was used as a negative control. Data shown are means ± SEM from three independent experiments. **P < 0.01, *** P < 0.001, unpaired Student’s t-test. c. Competitive proliferation assay was conducted in Cas9 stably expressed MOLM13 cells after transduced with GFP reporter-based lentiviral sgRNAs (NT, RPA3, GTF2A2-sg1, STAG2-sg1, MED30-sg1 and DHX15-sg1) at about ~50% efficiency. The GFP% was quantified on days 3, 6, 9, 12, and 15 by flow cytometry to evaluate the growth effect. The guide RNA targeting the survival essential gene RPA3 was included as a positive control, and the guide RNA targeting the non-target (NT) gene was included as a negative control. Data shown are means ± SEM from three independent experiments. * P < 0.05, ** P < 0.01, *** P < 0.001, unpaired Student’s t-test.*

The authors stated that RBM5 is selectively essential for AML cells but did not examine the effects of RBM5 ablation in other cancer cell lines. In the DepMap database, it seems lung adenocarcinoma and skin cancer are also required upon RBM5 deletion. Therefore, can the authors evaluate the effects of RBM5 in other cancer cell lines or remove the term "selectively".

*Response: Thank you for the reviewer’s comments. According to the suggestions, we first analyzed the survival score of RBM5 in both lung adenocarcinoma and skin cancer cell lines from the DepMap database. We confirmed that RBM5 was indeed required for the survival of some of the cell lines (See below: Response Figure R3.A). In addition, we added the analysis for the expression correlation between the RBM5 and HOXA9 among the lung cancer cell lines and skin cancer lines. Of note, there is no significant correlation between RBM5 and HOXA9 mRNA in lung and skin cancer cell lines (See below: Response Figure R3.B). Therefore, RBM5 may participate in lung and skin cancer development through a different mechanism, which needs further comprehensive study. As the reviewer pointed out, our analysis suggests that RBM5 is required for AML cell context and may participate in other solid tumors’ contexts. Therefore, we removed the term “selectively” in the revised manuscript.*

*Response Figure R3. Analysis for RBM5 dependency in lung cancer and skin cancer cell lines. A. Gene essential score of RBM5 in both lung cancer cell lines (left panel) and skin cancer cell lines (right panel) from Depmap. B. Scatter plot analysis comparing RBM5 mRNA levels and HOXA9 mRNA levels across lung cancer and skin cancer cell lines (data was obtained from the Depmap), respectively. P value is calculated by linear regression.*

The authors performed the reporter screen and most of the initial experiments with CRISPR/Cas9 but later changed to shRNA. What was the rationale for switching to different systems? Can the authors confirm the some of the findings from their shRNA experiments using CRISPR (e.g., RBM5 KO and HOXA9 expression, RBM5 KO induces AML differentiation)?

*Response: Thank you for the reviewer’s comments. First, both CRISPR/Cas9 mediated knockout and RNA interference (RNAi) were loss-function strategies to confirm the effect of RBM5 in AML. Due to the potential double-strand break effects other than the on-target effect of the genes[8], shRNA provides a complementary approach to studying the loss-of-function effect. Moreover, the vector size is much smaller in RNAi compared to the CRISPR/Cas9. Therefore, it would be easier to validate the function in primary AML patient samples or normal primary cells, given the limited number of primary cells and lower transduction efficiency.*

*According to the reviewer’s suggestions, we have added cell apoptosis and differentiation analysis in the RBM5 KO cells (See below: Additional file 1: Fig. S3d and S3e). The HOXA9 expression validation data was included in the original Fig. 6c and is now unchanged.*

*Additional file 1: Fig. S3 d. Representative flow cytometry analysis was conducted to assess propidium iodide (PI) and Annexin-V staining following lentiviral transduction of MOLM13 and OCIAML2 cells on day 6. Quantification of apoptotic cells, an average of 3 biological replicates. ** P < 0.01, unpaired Student’s t-test was applied to calculate the P value. e. Surface expression of CD11b and CD14 after lentiviral transduction of sgRNA against control NT and sgRNAs targeting RBM5 (sg1 and sg2) in MOLM13 and OCIAML2 cells. MFI, mean fluorescence intensity. * P < 0.05, ** P < 0.01, unpaired Student’s t-test.*

In Figure 5, the authors perform an extensive transcriptomic and splicing analysis of RBM5 perturbation but only concluded that there were no splicing changes associated with AML survival genes. Moreover, no splicing changes observed in HOXA9. Are any of the HOXA5 or HOXA9 targets mis-spliced upon RBM5 loss?This is currently the major area that is lacking which is how RBM5 regulating HOXA9. I highly recommend that the authors elucidate how RBM5 driving HOXA9 expression since this is the major takeaway from this paper.

*Response: Thank you for the reviewer’s comments. We now added the splicing analysis of the HOXA5 or HOXA9 targets to answer the question. Of note, we observed that splicing events of those targets after RBM5 loss in AML were unchanged (See below: Additional file 1: Fig. S6g).*

*To elucidate how RBM5 drives HOXA9 expression, we utilized two major strategies (inducible loss of function and gain of function system, ChIP-qPCR assay) to understand whether RBM5 controls HOXA9’s transcription directly or indirectly, as described in detail at the beginning (in the revised Fig. 6 and Additional file 1: Fig. S7).*

*Additional file 1: Fig. S6g. Pie charts showing the distribution of the HOXA9-related downstream target genes and their undergoing altered splicing events after RBM5 knockout (from RNA-seq dataset).*

Related to above, is RBM5 directly interacting with HOXA9 mRNA? This could help explain whether its regulation is direct or indirect (via another downstream gene).

*Response: Thank you for the reviewer’s comments. To better understand whether RBM5 controls HOXA9’s transcription directly or indirectly, we utilized two major strategies (inducible loss of function and gain of function system, ChIP-qPCR assay) and proved that RBM5 directly occupied HOXA9 gene locus and activated its transcription in an acute manner (in the revised Fig. 6). Moreover, from the splicing analysis, we had ruled out that the splicing of HOXA9 mRNA did not change after RBM5 loss or overexpression (in the revised Additional file 1: Fig. S7f). We agreed that it is more convincible to rule out the mRNA regulation by CLIP or RIP assay and distinguish the mRNA target from the DNA target, which requires further comprehensive studies based on more high-throughput techniques such as CLIP-seq and ChIPseq. We could not provide this evidence at the current stage and discussed this limitation in the revised manuscript (lines 456-461, second paragraph of page 12).*

Minor points:

HOXA9 expression has been shown to correlate with poor prognosis in AML, does RBM5 expression also correlate with poor survival?

*Response: Thank you for the reviewer’s comments. We analyzed the correlation between RBM5 expression and survival rate using patient datasets from the TCGA cohort and TARGET Cohort. In the TCGA AML cohort, mainly with adult patients, RBM5 expression did not significantly correlate with survival (See below: Response Figure 4A). However, the RBM5 lower expression group was positively and significantly associated with longer survival time from the TARGET cohort with young patients, suggesting RBM5 expression might be a potential prognostic marker in the young AML cohort (See below: Response Figure 4B).*

*Response Figure R4. Correlation analysis between RBM5 expression and AML patients’ survival. A. RBM5 expression and survival rate from the TCGA cohort. B. RBM5 expression and survival rate from the TARGET cohort. The patient size was shown as n in each group.*

For their AML verses normal comparison, what normal tissues were used from GTEx? Are these total bone marrow, HSC-enriched or PBMCs? A better description is needed for this analysis.

*Response: Thank you for the reviewer’s comments. We confirmed that the normal tissues from GTEx represented “bone marrow.” The detailed information could be found at this link http://gepia2.cancer-pku.cn/#dataset. We have now clarified this point in the revised Figure legends.*

For Figures 4, 6, 7 and supplemental figures, it would be better to change RBM5 MUT to RBM5 sgRNA-resistant or use PAM-MUT to not confuse future reads that the MUT stands for a mutation generated from the cDNA constructs.

*Response: Thank you for the reviewer’s comments. We have changed all the “RBM5 MUT” to the “PAM-MUT” in the revised draft (in the revised Fig. 2d, Fig. 2e, Fig. 2f, Fig. 4d, Fig. 6j, and Fig. 7g; Additional file 1: Fig. S2e, Fig. S7d, Fig. S7e).*

There is a misspelling of PWWPA3 on line 282.

*Response: Thank you for the reviewer’s comments. We apologized for the typos and changed this in the revised draft paragraph (line 303, second paragraph of page 8). Moreover, we carefully checked our text and corrected all the grammar mistakes and typos.*

**Reviewer 2**

**Were you able to assess all statistics in the manuscript, including the appropriateness of statistical tests used?**

No, I do not feel adequately qualified to assess the statistics.

**Comments to author:**

High level HOXA9 is important for stem cell expansion and is associated with certain subtypes of AML (MLL rearranged, Nup98-HOXA9, NPMc). Therefore the mechanisms of how HOXA9 is regulated, including by upstream transcription factors is important. This study uses a genome wide CRISPR/Cas9 screen of a HOXA9 reporter leukemia cell line along with a variety of correlative and loss of function and gain of function approaches to establish an important role for the RNA-binding protein RPM5 in for upregulating HOXA9 expression. Of note, RBM5 is highly expressed in AML cell lines and tends to be correlated with HOXA9 expression.

The work is thorough, looking at both cell lines and primary leukemia cells, in vitro and in and the data clearly support a role for RPM5 in upregulating HOXA9 expression, and also this is important for leukemia cell proliferation. This paper should be of interest to those studying mechanisms of leukemogenesis as well as non-canonical roles for splicing factors.

*Response: Thank you for these kind comments.*

Comments:

1. What remains an open question is whether the mechanism of HOXA9 upregulation is direct regulation of transcription as shown in Figure 8, or indirect through some other mechanism. This is the main conclusion of the manuscript. The authors make an argument that it is not a change in RNA splicing that is involved, and also that the RBM5 DNA binding domain is involved, but it seems possible that a change in splicing in some other transcription factor than RPM5 might be involved. This issue could be effectively addressed by ChIP for RBM5 which would establish whether RBM5 actually binds to the HOXA9 locus.

*Response: Thank you for the reviewer’s comments and suggestions. To elucidate how RBM5 drives HOXA9 expression, we utilized two major strategies to understand whether RBM5 controls HOXA9 transcription directly or indirectly (See next page： new Fig. 6 and Additional file 1: Fig. S7). We added this part of the results in the revised manuscript (lines 322-347, second paragraph of page 9; lines 353-357, first paragraph of page 10).*

*Firstly, we reasoned that the direct regulation of HOXA9 gene transcription would occur in an acute manner. Therefore, we utilized the auxin-induced degron (AID) system and generated two endogenous RBM5 N-terminus AID knock-in KMT2A-r leukemia lines (SEM homozygous clones and MOLM13 bulk cells). Those cell lines were then transduced by lentiviral vectors constitutively expressing OsTIR1(F74G) [1,2]. With the presence of small molecule 5-Ph-IAA, the OsTIR1(F74G), as a substrate receptor in Skp1, Cullin, F-box (SCF) complex, could target and mediate specific AID-tagged proteins for ubiquitination and degradation (in the revised Fig. 6d). Of note, after 2 hours post-treatment, the immunoblotting analysis showed that the AID-RBM5 fusion protein was undetectable. Consequently, the HOXA9 mRNA level was significantly reduced in two SEM knock-in clones after 2 hours of treatment and remained low until 24 hours (in the revised Fig. 6e). Similar effect was also observed after 24 hours of 5-Ph-IAA treatment in MOLM13 knock-in bulk populations (in the revised Fig. 6f). While conversely, we utilized an inducible overexpression system with the TRE3G promoter in the MOLM13 cell line. With the treatment of doxycycline for 2 hours and after that, we found significant upregulation of HOXA9 mRNA level accompanied by the increase of RBM5 expression (in the revised Fig. 6g). Similar effects were observed by overexpressing an exogenous RBM5-N terminus AID fusion protein (in the revised Additional file 1: Fig. S7g). Strikingly, upon the addition of 5-Ph-IAA in the presence of OsTIR1 (F74G), a significant* reduction of HOXA9 mRNA was observed after 2 hours of treatment and after that (in the revised Additional file 1: Fig. S7g). Taken together, those results indicate that RBM5 directly activated the HOXA9 transcription.

*Secondly, given that RBM5 regulates the transcriptional level of HOXA9 through both RNA and DNA binding domains, we hypothesized that RBM5 may directly bind to the HOXA9 gene locus. To test this, we performed an anti-MYC tag antibody ChIP (Chromatin Immunoprecipitation) to analyze the chromatin occupancy of exogenous RBM5-MYC expressed in OCIAML2 leukemia cells. Importantly, we observed significant enrichment of RBM5-MYC on the downstream region of HOXA9, together with active transcription mark H3K4me3 (in the revised Fig. 6i; Additional file 1: Fig. S7h). These results suggest that RBM5 occupied the HOXA9 gene locus and directly activated its transcription.*

*New Fig. 6. HOXA9 is a functional target gene of RBM5 in AML. a. Scatter plot analysis comparing RBM5 mRNA levels and HOXA9 mRNA levels across acute myeloid leukemia cell lines (data was obtained from the Depmap). P value is calculated by linear regression. b. Immunoblotting of RBM5 and HOXA9 in various leukemia cell lines and normal bone marrow (NBM) samples (left panel). The bar chart displays the quantitative protein levels of RBM5 and HOXA9 in various cell lines depicted in the left panel (right panel). c. Real-time-qPCR and Immunoblotting analysis was conducted on the RBM5-sg1, RBM5- sg2, and sgNT targeted MOLM13 and OCIAML2 to monitor the expression of HOXA9. Data shown are means ± SEM from three independent experiments. * P < 0.05, unpaired Student’s t-test. d. Diagram of auxin-induced degron (AID) system to degrade endogenous RBM5. Endogenous RBM5 N-terminus AID knock-in KMT2A-r leukemia lines (SEM homozygous clones and MOLM13 bulk cells) were followed by constitutively expressing OsTIR1(F74G). With the presence of small molecule 5-Ph-IAA, the OsTIR1(F74G), as a substrate receptor in Skp1, Cullin, F-box (SCF) complex, could target and mediate specific AID-tagged RBM5 proteins for ubiquitination and degradation. e. The protein level of endogenous RBM5-AID of SEM homozygous clones can be acutely degraded with 5-Ph-IAA through degron-mediated proteasome degradation after 2 hours post-treatment (Left panel). HOXA9 mRNA level was significantly reduced after 2 hours, 4 hours, 6 hours, and 24 hours of treatment in SEM cells (Right panel). f. After 24 hours of treatment with 5-Ph-IAA in HA-miniAID-RBM5 knock-in cells, the protein level of Knockin-RBM5 decreased (Left panel), and there was also a significant reduction in HOXA9 mRNA level (Right panel). g. The doxycycline-Tet-On system for ectopic overexpression of RBM5-HA in the MOLM13 cell line, after a 2-hour treatment with doxycycline, a significant upregulation in HOXA9 mRNA levels was observed (Right panel), accompanied by an increase in RBM5 expression (Left panel). h. Real-time-qPCR analysis was conducted in OCIAML2 cells transduced with RBM5 cDNA wildtype (WT) or six individual truncated mutants to validate the expression of HOXA9. Data shown are means ± SEM from three independent experiments. *P < 0.05, unpaired Student’s t-test. i. ChIP-qPCR with anti-MYC tag antibody near H3K4me3-bound HOXA9 downstream region (Chr7:27,199,969-27,200,853) in OCIAML2-RBM5-MYCtag cells (n=3). The red arrows indicate the primer 1 and primer 2 located position, and the blue arrow indicates the negative control (NC) primer located position, where there is no H3K4me3 signal. Statistical analysis (P value) was performed using an unpaired Student’s t-test. All error bars represent mean ± SEM. j. Rescued competitive proliferation assay was conducted by infecting OCIAML2-Cas9 and MOLM13-Cas9 cells overexpressing ectopic RBM5-wild type cDNA (WT), RBM5-sgRNA1 resistant mutant cDNA (PAM-MUT), and HOXA9 cDNA (linked to Venus reporter) with lentiviral-sgRNAs against non-target (sgNT) and RBM5 (RBM5-sg1) at about 50% efficiency (all monitored by Venus reporter). Data shown are means ± SEM from three independent experiments. ** P < 0.01, unpaired Student’s t-test.*

*Additional file 1: Fig. S7. HOXA9 is a functional target gene of RBM5 in AML. a. Flow cytometry analysis of the OCIAML2-HOXA9P2A-mCherry cells targeted with Nontarget-sgRNA (sgNT), RBM5-sgRNA-2 (sgRBM5), sgRBM5+TRE3G-RBM5-HA-DoX (RBM5-KO) and sgRBM5+TRE3G-RBM5-HA+DoX (RBM5- OE). b. Real-time-qPCR and Immunoblotting analysis was conducted on the shRBM5#1(sh#1), shRBM5#2(sh#2), and shNT targeted OCIAML2 and MOLM13 to monitor the expression of HOXA9. Data shown are means ± SEM from three independent experiments. * P < 0.05, ** P < 0.01, *** P < 0.001, unpaired Student’s t test. c. Immunoblotting of HOXA9 in RBM5 sgRNAs and shRNAs targeted THP1. The β-ACTIN was used as a reference. d. Real-time-qPCR analysis was conducted by infecting MOLM13 cells overexpressing ectopic Venus empty vector, RBM5-WT, and RBM5-PAM-MUT. Data shown are means ± SEM from three independent experiments. * P < 0.05, *** P < 0.001, unpaired Student’s t-test. e. Immunoblotting was conducted by infecting MOLM13-Cas9, OCIAML2-Cas9 and THP1-Cas9 cells overexpressing ectopic Venus empty vector, RBM5-WT, and RBM5-PAM-MUT. f. Sashimi plots the gene HOXA9 in RBM5 OE/CTRL and RBM5 KO/ CTRL group in MOLM13 cells. g. Schematic depicting the establishment of an inducible ectopic RBM5-AID degradation system in MOLM-13 cells. The overexpression of RBM5-AID can be acutely degraded through degron-mediated proteasome degradation with 5-Ph-IAA treatment. (Upper panel). The OsTIR1/mini-AID-mediated degradation efficiently decreased the RBM5-AID protein level after 2 hours of treatment, accompanied by a significant reduction of HOXA9 mRNA (Below panel). h. ChIP-qPCR with a H3K4me3 antibody for HOXA9 locus in OCIAML2-RBM5-MYCtag cells (n = 3). Statistical analysis (P value) was performed using an unpaired Student’s t-test. All error bars represent mean ± SEM. i. Immunoblotting was conducted by infecting OCIAML2 and MOLM13 cells overexpressing ectopic Venus empty vector and HOXA9 cDNA. **P < 0.01, ***P < 0.001, unpaired Student’s t-test.*

2. Figure 4b-4d- It looks like all the mutated domains result in decreased HOXA9 expression. The authors should acknowledge the possibility that this is lost of function because of an allosteric effect.

*Response: Thank you for the reviewer’s comments. We admitted this limitation that different truncated domain mutants might lose their function by an allosteric effect even with similar expression levels. We discussed this limitation by adding the related reference in the revised manuscript (lines 459-461, second paragraph of page 12). Moreover, this strategy was complementary to the protein domain screen assay. Those results suggest that RBM5 relies on both RRM and zinc-finger domains.*

3. Fig. 3K- if the authors have morphology (Wright-Giemsa stained cells) on the cells in Fig 3d and 3k this should be included.

*Response: Thank you for the reviewer’s comments. We added morphology data on the cells in Fig 3d (See below: Additional file 1: Fig. S3b). However, for the cells in Fig. 3k, we have limited cell numbers for WrightGiemsa staining from the original samples, and there were no samples left for the morphology assay. Therefore, we could not repeat the entire experiment to perform morphological analysis from the same donor sample but will do so if the reviewer and editor deem it necessary to use a separate donor sample.*

*Additional file 1: Fig. S3b. Gimesa staining was performed on cell morphology after lentiviral transduction of shRNA against control shNT and shRNAs targeting RBM5 (sh#1 and sh#2) in MOLM13 and OCIAML2 cells.*

4. Page 9, line 344- regarding the expression of FLT3 is regulated by RBM5/HOXA9, the authors should be aware that Flt3 has been shown to be a directly upregulated target of Hoxa9, at least in murine cells (Huang et al. Blood 2012 Jan 12; 119(2): 388-398). It would be beneficial to cite this.

*Response: Thank you for the reviewer’s comments.*

*Regarding how RBM5 and HOXA9 regulate FLT3, we have demonstrated that FLT3 mRNA and protein level was reduced after RBM5 knockout (in the original Fig. 7d and 7e). As the reviewer pointed out, it is known that HOXA9 directly occupies the FLT3 locus and activates its transcription[4]. This is consistent with our evidence that HOXA9 could restore the FLT3 expression in RBM5 null leukemia cells (in the original Fig. 7d and 7e). To elucidate how RBM5 drives HOXA9 expression, we utilized two major strategies (inducible loss of function and gain of function system, ChIP-qPCR assay) to support that RBM5 directly activated the transcription of HOXA9 (in the revised Fig. 6 and Additional file 1: Fig. S7). Taken together, our data suggests that RBM5 may activate FLT3 through upregulating HOXA9. We discussed this point by adding the related reference (line 465-475, second paragraph of page 12). Moreover, we have modified our proposed model (in the revised Fig. 8), highlighting the potential RBM5/HOXA9/FLT3 regulatory axis.*

5. In a number of places the manuscript needs some editing for word choice and syntax. For example, it is difficult to determine what is meant about "lame characterization" line 196 on page 6.

*Response: Thank you for the reviewer’s comments. We now changed the text “all mice receiving control cells exhibited lame characterization 18 days post-injection” to “RBM5 shRNAs-treated cells significantly improved the overall survival of the mice compared to the control cells” in the revised draft (line 207-209, second paragraph of page 6). Moreover, we carefully checked our text and corrected all the grammar mistakes and typos.*

*References: 1. Hyle J, Djekidel MN, Williams J, Wright S, Shao Y, Xu B, et al. Auxin-inducible degron 2 system deciphers functions of CTCF domains in transcriptional regulation. Genome Biol. 2023;24:1–30. 2. Yesbolatova A, Saito Y, Kitamoto N, Makino-Itou H, Ajima R, Nakano R, et al. The auxin-inducible degron 2 technology provides sharp degradation control in yeast, mammalian cells, and mice. Nat Commun. 2020;11:5701.*

*3. Zhang H, Zhang Y, Zhou X, Wright S, Hyle J, Zhao L, et al. Functional interrogation of HOXA9 regulome in MLLr leukemia via reporter-based CRISPR/Cas9 screen. Elife. 2020;9:1–30.*

*4. Huang Y, Sitwala K, Bronstein J, Sanders D, Dandekar M, Collins C, et al. Identification and characterization of Hoxa9 binding sites in hematopoietic cells. Blood. 2012;119:388–98.*

*5. O’Bryan MK, Clark BJ, McLaughlin EA, D’Sylva RJ, O’Donnell L, Wilce JA, et al. RBM5 Is a Male Germ Cell Splicing Factor and Is Required for Spermatid Differentiation and Male Fertility. Barsh GS, editor. PLoS Genet. 2013;9:e1003628.*

*6. Mullari M, Fossat N, Skotte NH, Asenjo-Martinez A, Humphreys DT, Bukh J, et al. Characterising the RNA-binding protein atlas of the mammalian brain uncovers RBM5 misregulation in mouse models of Huntington’s disease. Nat Commun. 2023;14:4348.*

*7. Snyder K, Gorse K, Kochanek PM, Jackson TC. Neuronal RBM5 modulates cell signaling responses to traumatic and hypoxic-ischemic injury in a sex-dependent manner. Cell Death Discov. 2023;9:379.*

*8. Zhang X-H, Tee LY, Wang X-G, Huang Q-S, Yang S-H. Off-target Effects in CRISPR/Cas9-mediated Genome Engineering. Mol Ther - Nucleic Acids. 2015;4:e264.*

**Second round of review**

**Reviewer 1**

The authors have addressed all my concerns.

I looked over in detail the author’s response to Reviewer 2 and in my opinion they have addressed all the Reviewer’s concerns very well.
